# ﻿Forty-two years of scientific research on eight legs—celebrating the 60^th^ birthday of Dr Yuri M. Marusik

**DOI:** 10.3897/zookeys.1100.85374

**Published:** 2022-05-13

**Authors:** Jianshuang Zhang, Kirill G. Mikhailov, Seppo Koponen, Feiyang Long, Hao Yu, Shuqiang Li

**Affiliations:** 1 The State Key Laboratory of Southwest Karst Mountain Biodiversity Conservation of Forestry Administration, School of Life Sciences, Guizhou Normal University, Guiyang, China; 2 The Key Laboratory of Plant Physiology and Development in Guizhou Province, School of Life Sciences, Guizhou Normal University, Guiyang, China; 3 Zoological Museum, Moscow Lomonosov State University, Moscow, Russia; 4 Zoological Museum, University of Turku, Turku, Finland; 5 School of Biological Sciences, Guizhou Education University, Guiyang, China; 6 Institute of Zoology, Chinese Academy of Sciences, Beijing, China

**Keywords:** Arachnology, Araneae, bibliography, catalogue, nomenclature, patronyms, taxonomy

## Abstract

The biography of Dr Yuri Mikhailovich Marusik is presented, and his scientific life illuminated on the occasion of his 60^th^ birthday. Yuri is a renowned specialist of spiders. He has described 718 new species, 57 new genera, and two new subfamilies of order Araneae. Twenty-five species and one genus have been dedicated to him. Facts and impressions are given as well as a bibliography of his 545 publications.

## ﻿

We are fortunate to know Yuri not only as an excellent professional arachnologist and enthusiastic colleague but also as a good friend. Yuri turns 60 years on May 13^th^, and we would like to thank him for all of his contributions and wish him many more years of achievements. Here we present some facts, impressions and memories from his life. A biography of Yuri and a complete list of his publications, patronyms in honour of him, and new Araneae published by him are presented here.

## ﻿Family background

Yuri Mikhailovich Marusik was born on May 13^th^, 1962 in Sarny, a small town in West Ukraine, in a family of a school teacher (father Mikhail Adamovich) and a nurse (mother Tamara Andreevna). He has one brother, Andriy, who is 13 years younger. Following in Yuri’s footsteps, Andriy became a biologist and now works as an associate professor at Moffitt Cancer Center in Tampa, Florida where his lab is studying the evolutionary and ecological underpinnings of resistance to cancer therapies. Before Yuri went to school, the family lived in Hrytsiv, a village in the Shepetovka District of the Khmelnytskyi Region, Ukraine. During his school years, the Marusik family moved to Rovno (now Rivne). Yuri’s interest in zoology arose from reading books about animals and spending summer school breaks in Hrytsiv. Yuri speaks Russian, Ukrainian, English and Polish.

In Hrytsiv he learned how to herd cows, bail hay, fish with a rod and catch crayfishes with his hands, and harvest apples, potatoes and other crops. While studying zoology, Yuri learned photography, including macrophotography.

## ﻿Education

Yuri continued his studies at Leningrad (now St. Petersburg) State University in 1979 where he specialized in entomology under the prominent Professor Victor P. Tyshchenko, a specialist in insect physiology. Tyshchenko produced the first detailed identification key to spiders from the European part of the USSR, the only such book in Russian language. In the 1980s, Leningrad was the centre of arachnological research in the USSR due to the presence of the aforementioned Victor P. Tyshchenko, as well as Vladimir I. Ovtsharenko from the Zoological Institute which harboured the largest collection of animals in the country and Alexei A. Zyuzin, an expert in Lycosidae. Many visitors stayed with Yuri and he learned a lot from them, establishing personal relationships with most Soviet arachnologists. While studying at the university, Yuri decided to specialize on the taxonomy of orb-web spiders.

After the graduation in 1984, Yuri was accepted into the Ph.D. program in Magadan, Northeastern Siberia, working in the lab of Daniil I. Berman. This laboratory conducted complex studies of the interrelationships of invertebrates and poikilothermic vertebrates, their spatial distribution, cold resistance and macro- and microclimates. Yuri became the first Soviet arachnologist to settle in Northeastern Siberia, making him the easternmost arachnologist in the Northern Hemisphere.

When Yuri began to study spiders from East Siberia, he recognized that it was not possible to identify most of the species, even with the help of his senior colleagues Vladimir I. Ovtsharenko and Alexei A. Zyuzin. It was difficult to acquire literature in the Soviet Union in the 1980s, and impossible to photocopy articles and books due to censorship.

Yuri’s Ph.D. dissertation (1988) was devoted to fauna, population structure and spatial distribution of spiders in the upper reaches of the Kolyma River, Northeastern Siberia. Nearly 350 species were found in the area, with 170 of them new to science. Several of these species were previously known only from North America. The most diverse spider group in Northeastern Siberia is Linyphiidae. At this time, the taxonomy of Linyphiidae and particularly those in Siberia was poorly developed. Kirill Y. Eskov and Andrei V. Tanasevitch, both from Moscow, made great advances to the study of this family.

## ﻿Contributions to academia

**Figure 1.**
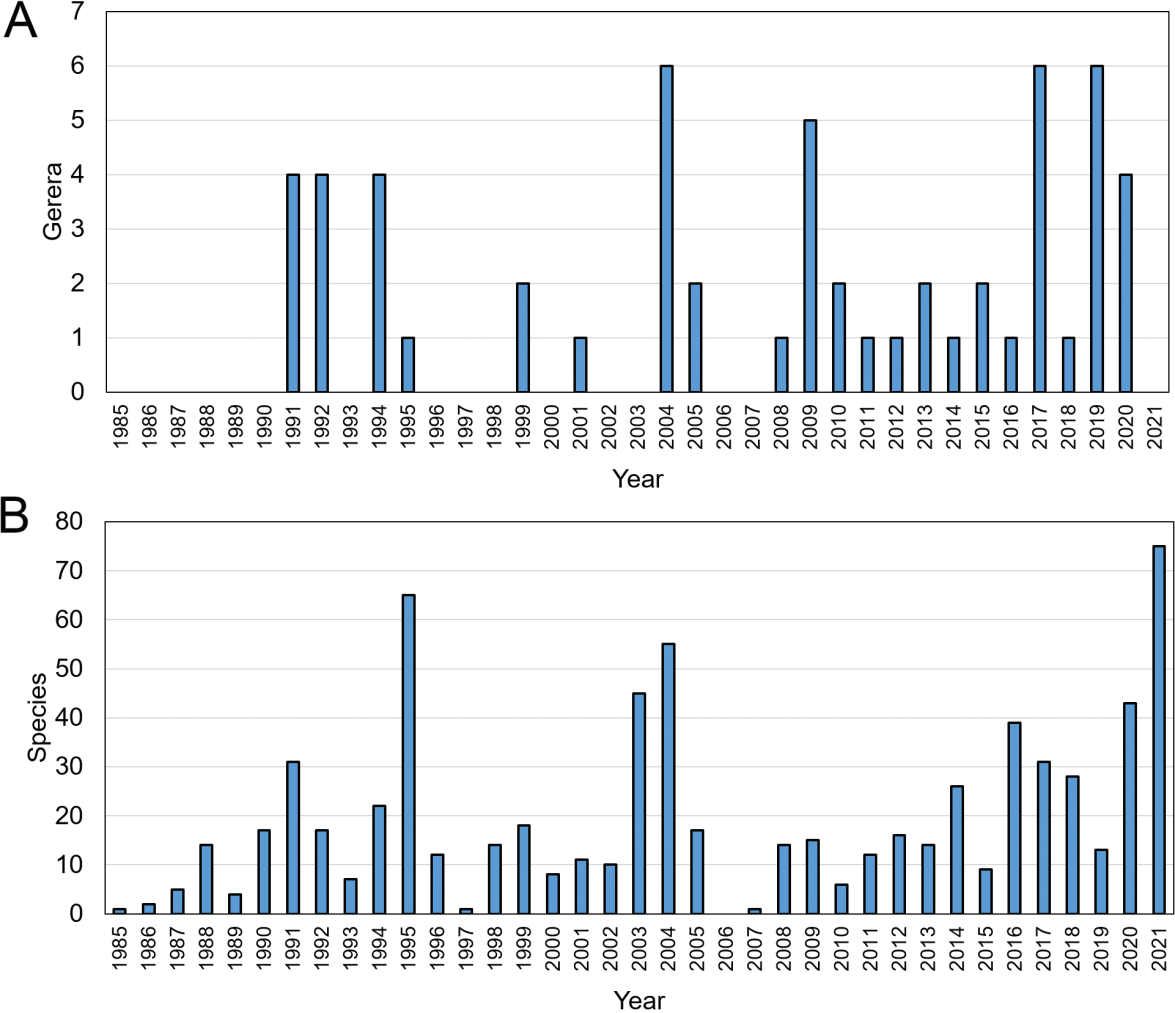
**A** Annual count of new Araneae genera and **B** species described by Yuri M. Marusik from 1985–2021.

Yuri has published 545 papers dealing with taxonomy, systematics, faunistics and the biogeography of spiders and other invertebrates (WSC 2022; Suppl. material 1: List 1, book reviews are not included). He has described 57 new genera and 718 new species of spiders over four decades career, an average of 21 names and 20 species annually (Table 1; Fig. 1; Suppl. material 1: List 2).

**Table 1.** Numbers of Araneae taxa described by Yuri M. Marusik (percentages of total counts in parentheses). Abbreviations: hom. (homonym/s); syn. (synonym/s); fos. (fossil); id. (idem).

Higher Taxa	Subfamilies	Genera	Subgenera	Species	Names
Araneae	2	57 [4 syn., 1 hom.]	3	718 [12 fos., 32 syn., 2 hom.]	780 [12 fos., 36 syn., 3 hom.]
Opisthothelae	id.	id.	id.	id.	id.
Araneomorphae	id.	id.	id.	708[12 fos., 32 syn., 2 hom.] (98.60)	770 [12 fos., 36 syn., 3 hom] (98.71)
Agelenidae		5 (8.77)		40 [3 syn.] (5.57)	45 [3 syn.] (5.77)
Amaurobiidae	1	2 (3.51)		5 [1 syn.] (0.70)	8 [1 syn.] (1.03)
Anapidae				1 [1 fos.] (0.14)	1 [1 fos.] (0.13)
Araneidae				9 [1 syn.] (1.25)	9 [1 syn.] (1.15)
Cheiracanthiidae				1 (0.14)	1 (0.13)
Clubionidae				3 (0.42)	3 (0.38)
Ctenidae		1 (1.75)		1 (0.14)	2 (0.26)
Cybaeidae				7 (0.97)	7 (0.90)
Dictynidae				22 [1 syn.] (3.06)	22 [1 syn.] (2.82)
Dysderidae				4 (0.56)	4 (0.51)
Eresidae				2 (0.28)	2 (0.26)
Filistatidae		1 (1.75)		32 (4.46)	33 (4.23)
Gnaphosidae		5 [1 syn.] (8.77)		116 [1 hom., 1 syn.] (16.16)	121 [1 hom., 2 syn.] (15.51)
Hahniidae		1 (1.75)		4 (0.56)	5 (0.64)
Hersiliidae		4 (7.02)		15 (2.09)	19 (2.44)
Linyphiidae		19 [1 hom.] (33.33)		104 [10 syn.] (14.48)	123 [1 hom., 10 syn.] (15.77)
Liocranidae	1	2 (3.51)		9 (1.25)	12 (1.54)
Lycosidae		6 (10.53)		88 [1 hom., 3 syn.] (12.26)	94 [1 hom., 3 syn.] (12.05)
Miturgidae				1 (0.14)	1 (0.13)
Nesticidae				6 [2 fos.] (0.84)	6 [2 fos.] (0.77)
Oecobiidae				3 (0.42)	3 (0.38)
Oonopidae		3 [2 syn.] (5.26)		8 [1 fos.] (1.11)	11 [1 fos., 2 syn.] (1.41)
Palpimanidae		3 (5.26)		11 (1.53)	14 (1.79)
Philodromidae				7 [2 syn.] (0.97)	7 [2 syn.] (0.90)
Phrurolithidae		1 (1.75)		4 (0.56)	5 (0.64)
Pisauridae				2 (0.28)	2 (0.26)
Prodidomidae				1 (0.14)	1 (0.13)
Salticidae		1 (1.75)	3	91 [1 syn.] (12.67)	95 [1 syn.] (12.18)
Scytodidae				1 (0.14)	1 (0.13)
Segestriidae				4 (0.28)	4 (0.51)
Sicariidae				2 (0.14)	2 (0.26)
Stenochilidae				1 (0.14)	1 (0.13)
Synaphridae				3 (0.42)	3 (0.38)
Tetragnathidae		1 [1 syn.] (1.75)		13 [3 syn.] (1.81)	14 [4 syn.] (1.79)
Theridiidae		1 (1.75)		23 [8 fos., 2 syn.] (3.20)	24 [1 fos., 2 syn.] (3.08)
Thomisidae		1 (1.75)		36 [3 syn.] (5.01)	37 [3 syn.] (4.74)
Titanoecidae				3 [1 syn.] (0.42)	3 [1 syn.] (0.38)
Trachelidae				3 (0.42)	3 (0.38)
Zodariidae				22 (3.06)	22 (2.82)
Mygalomorphae				10 (1.39)	10 (1.28)
Bemmeridae				3 (0.42)	3 (0.38)
Cyrtaucheniidae				1 (0.14)	1 (0.13)
Nemesiidae				6 (0.84)	6 (0.77)

## ﻿Expeditions

**Figure 2.**
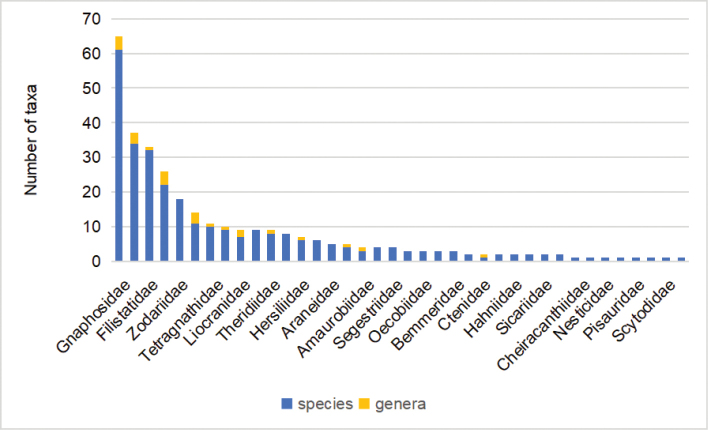
Number of new Araneae taxa described by Yuri M. Marusik after 2012, according to families and taxonomic category (blue—species: 294, yellow—genera: 24).

**Figure 3.**
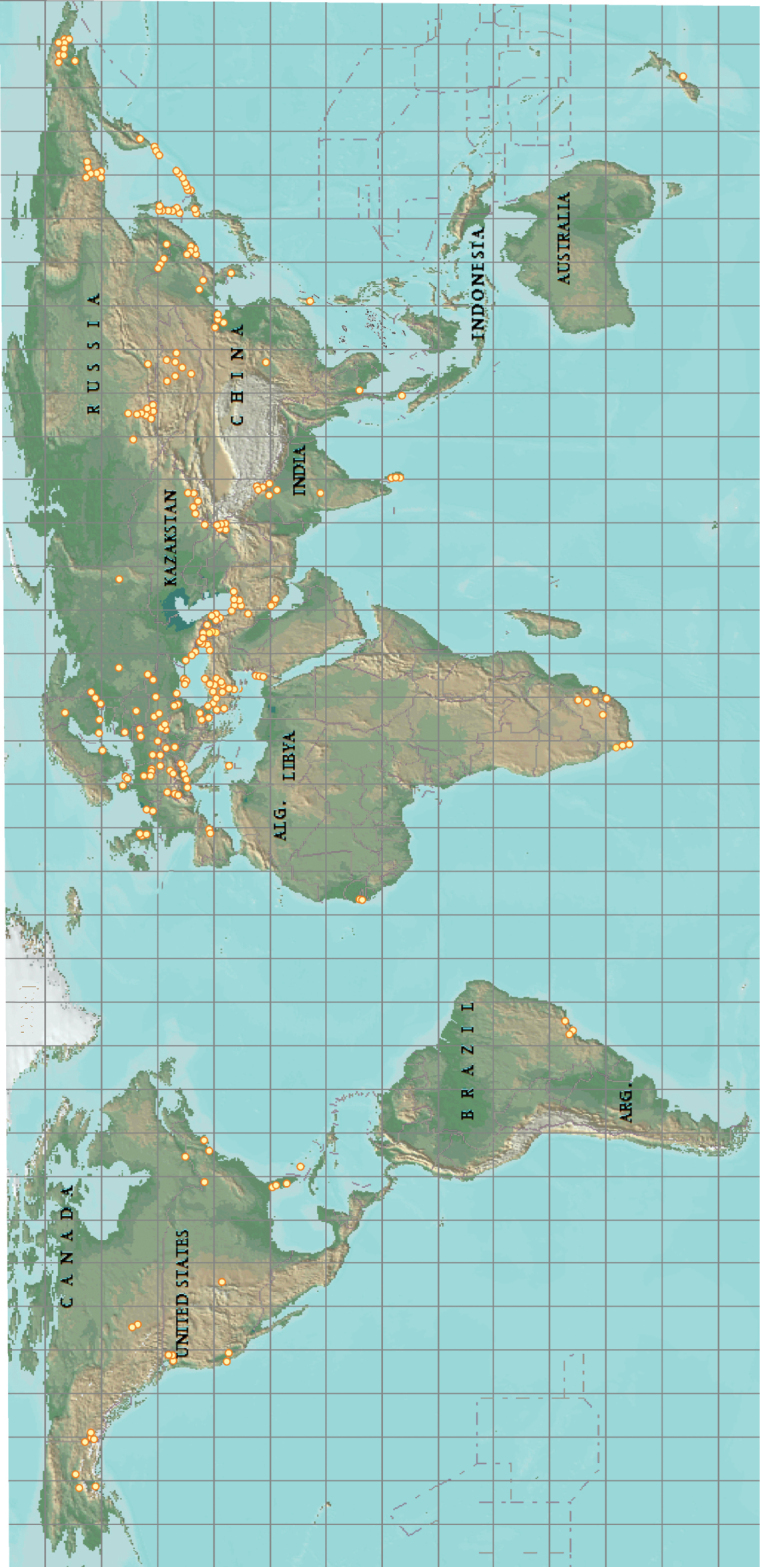
Expeditions and trips by Yuri M. Marusik up until 2022.

**Figure 4.**
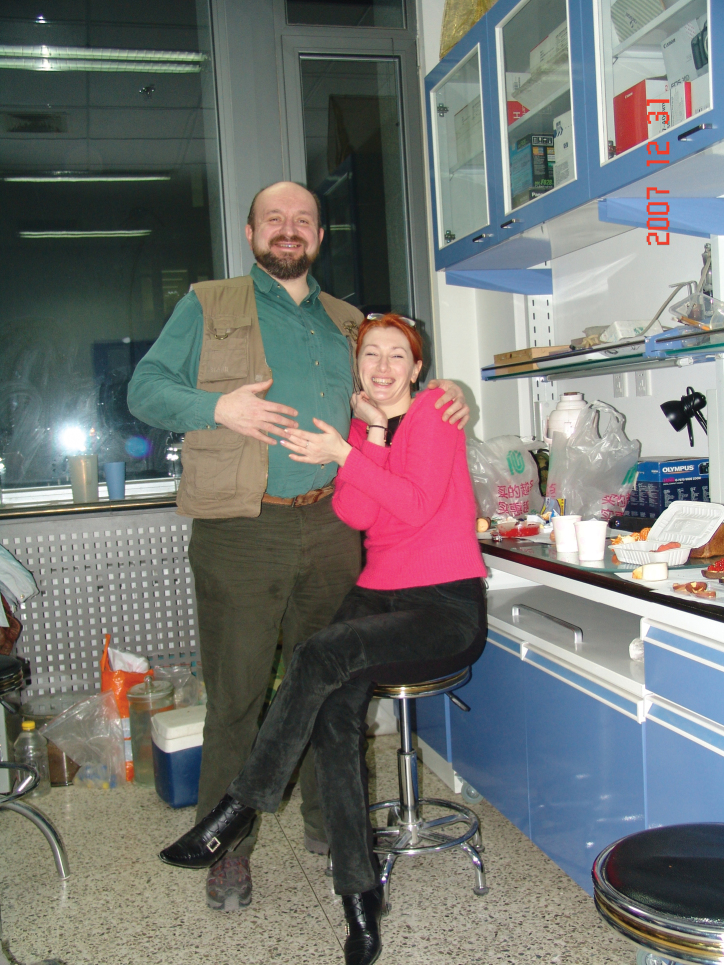
Celebrating the coming 2008, together with Irina Marusik. December 31, 2007 in Beijing, China.

**Figure 5.**
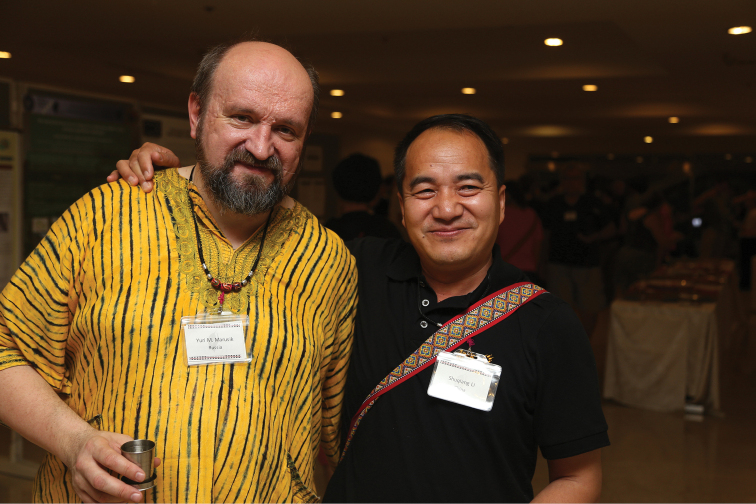
19^th^ International Congress of Arachnology in Kenting National Park, Taiwan, China. Together with Shuqiang Li, June 2013.

**Figure 6.**
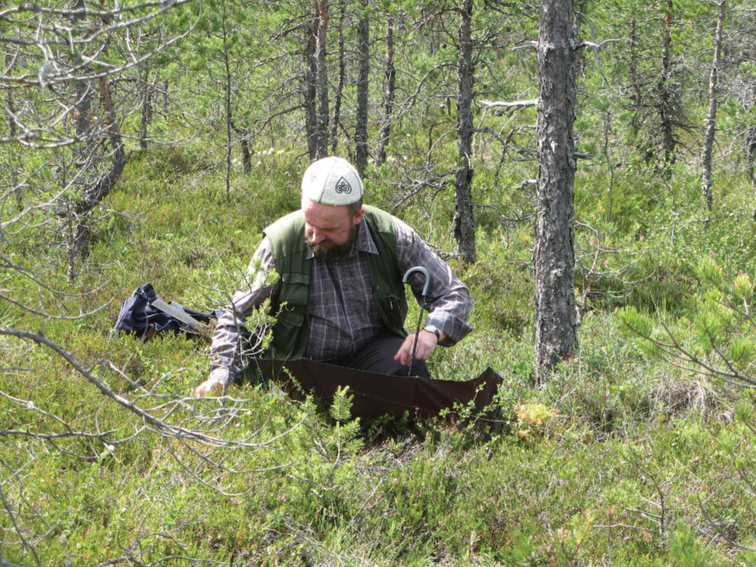
In peat bog, Finland, collecting spiders by beating, July 2011.

**Figure 7.**
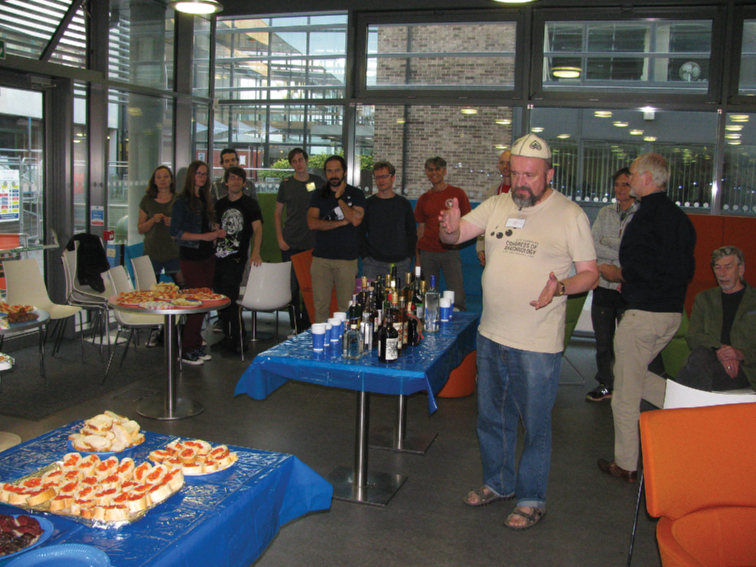
Russian Party, Nottingham, England, 2017.

Most of Yuri’s research has been based on the material and data he collected during fieldwork (Fig. 2). He has visited to 45 countries (Fig. 3), including Armenia, Azerbaijan, the Bahamas, Belarus, Belgium, Brazil, Bulgaria, Canada, China (Figs 4, 5), the Czech Republic, Denmark, Finland (Fig. 6), Gambia, Georgia, Germany, Greece, Hungary, India, Iran, Israel, Italy, Japan, Kazakhstan, Korea, Latvia, Lithuania, Moldova, Mongolia, Netherlands, New Zealand, Poland, Slovakia, Slovenia, South Africa, Spain, Sri Lanka, Sweden, Switzerland, Tajikistan, Turkey, UK (Fig. 7), Ukraine, USA and Uzbekistan. Yuri’s focus has been in the former USSR states and the Middle East, and most of the expeditions and congress trips were supported from his own salary or pension (Mikhailov and Logunov 2012). During his many fieldtrips, Yuri has collected a large number of species, hundreds described by him as new taxa. Spiders described by Yuri are distributed from the high latitudes of the Arctic (71°N, Wrangel Island) to the low latitudes of the southern hemisphere (33°S, South Africa).

Spiders collected by Yuri are dispersed in zoological museums worldwide, but those awaiting further study are temporarily kept in at the Zoological Museum in Turku (ZMT), Finland. In addition to spiders, Yuri has collected many other invertebrates. After returning from fieldwork, he sorted and diligently labelled the material, sending them to specialists that were willing to examine the specimens and revise the taxonomic groups. Unsurprisingly, many colleagues have dedicated taxa to Yuri, most commonly with the specific epithet “*marusiki*”. A total of 26 taxa are dedicated to him, including one genus and 25 species, across seven orders of Arachnida and Insecta (Suppl. material 1: List 3).

## ﻿Publications

Yuri’s publications can be categorised in the following subjects: faunistics and checklists, taxonomy, the history of arachnological studies and biographies of arachnologists as well as diverse works on other invertebrates. He has published in English, Russian, Persian, Japanese, Ukrainian and Finnish.

### ﻿Faunistics and checklists

Faunistic studies comprise the bulk of Yuri’s publications. Early in his career studying spiders of the far north, Yuri’s interest extended to adjacent Yakutia, Sakhalin, Mongolia and Northwestern North America. With the collaboration of his colleagues, he has published checklists of spiders of Northeastern Siberia, Yakutia, the Sakhalin Area, Yukon Territory, Tuva, Israel, Iran, Kenya, Greenland, Altai, Mongolia, Polar Ural region and Northern Cisokhotia (Suppl. material 1: List 1A), as well as some large reserves in Russia: Sokhondo, Bolshekhekhtsyrski, Lazo and Kamchatka (Suppl. material 1: List 1A: references 84, 96, 104, 151, 158, 242, 311, 360, 348, 367, etc.). These faunistic works have produced several lengthy monographs (more than 100 pages), detailed catalogues and mapped distribution data, providing valuable taxonomic resources for future arachnologists (Suppl. material 1: List 1A: references 62, 267, 297, 396, 497, etc.).

Perhaps for many arachnologists who focus on Palaearctic spiders, his most important contribution to arachnology is his monograph on the spiders of the Asian part of Russia: “Spiders (Arachnida, Aranei) of Siberia and Russian Far East” (2011, in collaboration with Mykola M. Kovblyuk; in Russian; Suppl. material 1: List 1A: reference 267). This work contains several original keys for identifying spider families and reviews of each family, including numerous colour illustrations. It is an indispensable reference for all aspiring arachnologists who want to work with spiders of Siberia and the Russian Far East (Mikhailov and Logunov 2012), and might be used in universities by students studying biology courses (for example, in Far Eastern Federal University, Mikhail M. Omelko use this book with his students during their field course). Before Yuri came to Magadan, only about 30 species were known from this region, with about 500 species known from all of Siberia and the Russian Far East. Because of his work, the number of species reported from Northeastern Siberia increased from 277 in 1989 to 421 in 2017 (or 454 if including the northern part of the Russian Far East) (Mikhailov 2021). It is no exaggeration that knowledge about the spiders of Siberia is largely based on the works of Yuri in the last 30 years.

In recent years, Yuri and his collaborators have intensively studied the spiders of Iran. An ongoing, large-scale faunistic series entitled “New Data on the Spider Fauna of Iran” is in progress, with eight parts published so far (Suppl. material 1: List 1A: references 459, 483, 484, 488, 489, 491, etc.). In collaboration with his Iranian colleagues, Yuri also maintains a website ‘http://spiders.ir/’ that contains all faunistic data of the spiders in Iran (Zamani et al. 2022).

### ﻿Taxonomy

Yuri is one of the most proliﬁc taxonomists today, having described about 1.4% of all spider species, more than any arachnologist from Russia. Even among arachnologist born in the Russian Empire, or those having lived in the Soviet Union, Yuri is the fourth most proliﬁc arachnologist in history, after E. Keyserling (born in Lithuania, Russian Empire, but worked on spiders after moving to Germany), Embrik Strand (born in Norway, worked actively while living in Germany before WWI, then moved to Latvia) and Alexander I. Petrunkevitch (born in Ukraine, Russian Empire, published papers after moving to the USA).

Yuri is interested in different spider families and considered an authority on numerous families. He has conducted research on Linyphiidae, Gnaphosidae and Lycosidae, and also dabbled in some medium- or small-sized spider families, such as Filistatidae, Trachelidae, Stenochilidae and others (Table 1; Suppl. material 1: List 2). Yuri has named taxa in 42 of the 130 families of spiders, in fossil and extant spiders, across both infraorders of Opisthothelae (Suppl. material 1: List 2).

Yuri’s early contributions to arachnological taxonomy (before 2012) have been discussed by Mikhailov and Logunov (2012). In our opinion, in the last ten years, his most important contributions to arachnology were the descriptions of many new taxa. Yuri has named 318 taxa in 19 families in the past ten years, focusing on Gnaphosidae (4 genera and 61 species), Lycosidae (3 genera and 34 species), Filistatidae (1 genus and 32 species), Agelenidae (4 genera and 22 species), Zodariidae (18 species), Palpimanidae (3 genera and 11 species), Tetragnathidae (1 genus and 10 species), and Linyphiidae (1 genus and 9 species) (Fig. 2; Suppl. material 1: List 2). From 2012 to 2021, Yuri has described 24 genera and 294 species of spiders (accounting for half of the total taxa described by him) reaching a career-high in 2021 describing 75 species (Fig. 1B; Suppl. material 1: List 2).

During the recent years, one of Yuri’s most important taxonomic contributions has been the redescription of known species, most of which were only known from the types. He redescribed numerous species, including the type species of at least 10 genera, refined some poorly studied genera, provided descriptions of unknown sexes and supplementary illustrations (many species were not illustrated or with poor illustrations), and updated the distribution information (Suppl. material 1: List 1A: references 111, 176, 305, 317, 326, 384, 513, 515, 516, 517, etc.). Yuri considers these redescriptions more important than descriptions of new species.

### ﻿History of arachnology

For a decade or more, Yuri has presented work on the history of arachnology, written many anniversary essays and edited festschrifts dedicated to domestic and non-Russian arachnologists (Suppl. material 1: List 1B), for example, to Andrei V. Tanasevitch, Dmitry V. Logunov, Jörg Wunderlich, Kirill G. Mikhailov, Kirill Y. Eskov, Michael I. Saaristo, Pekka T. Lehtinen, Seppo Koponen and Wanda Wesołowska. He has also published obituaries of some arachnologists (Suppl. material 1: List 1C), for example, to Da-Xiang Song, Gershom Levy, Michael I. Saaristo, Norman I. Platnick, Pontus Palmgren, Robin Leech, Takeo Yaginuma and Tamara S. Mcheidze. These publications provide invaluable information for future historians of arachnology in Russia and worldwide.

In the series “Restoring history of old names”, the biography of Adolf Grube was included. The paper “History and advances of spider taxonomy of the world” (2011, in Russian; Suppl. material 1: List 1A: reference 165) comprises very interesting data. He has written the preface for the book “The Field Guide of Spiders and Scorpions of Iran” (Zamani 2016), and also published reviews of the history of arachnological studies in Siberia and the Russian Far East, Caucasus, Iran, Israel and Kenya.

### ﻿Other contributions to invertebrate zoology (except arachnology)

Yuri has participated in studies of ecophysiology (e.g., cold resistance of spiders), earthworm fauna, taxonomy of scale insects (Coccoidea), true bugs (Heteroptera), booklice (Psocodea), millipedes (Diplopoda) and mites (Acari) as well as distribution patterns of Arctic invertebrates (Suppl. material 1: List 1A: references 1, 4, 6, 7, 8, 49, 67, 60, 61, 77, 113, 114, 142, 143, 393, 394, 430, 441, 442, 445, 446, 448, 457, etc.).

### ﻿Professional meetings activity

Yuri has participated in numerous arachnological congresses and meetings. His first international congress was the XI International Congress of Arachnology, 1989, in Turku, Finland. Since then, he has been a very active participant at numerous arachnological meetings organized by the International Society of Arachnology (Fig. 5), by European, American, Asian, Polish and Russian societies. Since the international congress in Brazil, 2007, his spouse Irina (“Ira”) has regularly accompanied him to arachnological events. In 1993, Yuri established the very popular tradition of a “Russian Party”, with salmon caviar and other sea products from Magadan, and strong (e.g., honey-pepper vodka) and milder drinks (Fig. 7). He has been active in organizing congress committees and helping to arrange the attendance of Russian-speaking colleagues. He was also the vice president of the International Society of Arachnology from 2007 to 2010.

## ﻿Collaboration and mentoring

Initially, Yuri actively collaborated with almost all Soviet taxonomists (Victor P. Tyshchenko, Kirill Y. Eskov, Andrei V. Tanasevitch, Dmitry V. Logunov, Kirill G. Mikhailov, Alexei A. Zyuzin and Chingiz K. Tarabaev). Although it was difficult to communicate with colleagues from other countries during the Soviet era, he managed to collaborate with Bruce Cutler (USA), Wanda Wesołowska (Poland) and Hirotsugu Ono (Japan), and had extensive correspondence with Pontus Palmgren, Herbert W. Levi, Jan Buchar, Jerzy Prószyński, Norman I. Platnick, Charles D. Dondale, Torbjörn Kronestedt, Konrad Thaler and others.

During the Soviet era it was almost impossible for scientists to travel abroad, and it was only at the end of Perestroika that Soviet arachnologists were able to visit other countries, and participate in the International Arachnological Congress in Turku (1989). In the early 1990s, funding for scientific research in Russia had been greatly reduced, and Yuri began to work abroad, mainly at the Zoological Museum of the University in Turku, Finland, where there was a strong group of arachnologists: Pekka T. Lehtinen, Michael I. Saaristo and Seppo Koponen. Frequent communication with senior Finnish colleagues contributed to the further development of Yuri as a scientist, and the first joint paper between Marusik and Koponen was soon published (Suppl. material 1: List 1A: 243). Yuri and several other colleagues participated in two INTAS (International Association for the Promotion of Cooperation with Scientists from the independent States of the former Soviet Union). These were established to promote cooperation between scientists from Western Europe and those from the former Soviet Union. They included projects co-ordinated by Seppo Koponen in 1995–97 and 2006–08 dealing with spider biodiversity at high altitudes in Eurasia and the diversity of spiders on Arctic islands, respectively. Yuri considers Saaristo and Lehtinen as the most influential mentors of his taxonomic career.

Yuri has worked regularly in Turku and has arranged, sometimes at his own expense, visits of colleagues from China (Ning Sun), Russia (Mikhail M. Omelko, Alexander A. Fomichev, Galina N. Azarkina), Ukraine (Mykola M. Kovblyuk, Anton A. Nadolny, Valery A. Gnelitsa), Slovakia (Anna Šestáková), Italy (Francesco Ballarin), Iran (Sepideh Shafaie), Azerbaijan (Elchin F. Huseynov), Kyrgyzstan (late S.V. Ovtchinnikov), Kazakhstan (Alexander V. Gromov), Israel (Sergei L. Zonstein) and Poland (Lukasz Trębicki). Many other arachnologists from the ex-USSR and Europe have also visited and worked in Turku. These research visits have produced several of joint publications. Due to Yuri’s activity, ZMT has been an international centre of arachnology for the last 25–30 years.

Yuri is one of the first Russian arachnologists to use extensive co-authorship since beginning of scientific career. Yuri has co-authored papers with colleagues from 30 countries. Co-authors of his prodigious monographic revisions and many smaller papers include Dmitri V. Logunov (Manchester Museum, England) and colleagues at ZMT: Alireza Zamani, Seppo Koponen, Pekka T. Lehtinen and Michael I. Saaristo (Table 2; Suppl. material 1: List 1). Additional collaborators include numerous prominent arachnologists, such as Norman I. Platnick (New York, U.S.A.), Charles D. Dondale (Ottawa, Canada), Da-Xiang Song (Baoding, China), Jörg Wunderlich (Hirschberg, Germany), Hirotsugu Ono (Tokyo, Japan), Kirill Y. Eskov (Moscow, Russia), Mikhail M. Omelko (Vladivostok, Russia), Alexander A. Fomichev (Barnaul, Russia), Kadir B. Kunt (Northern Cyprus), Mykola M. Kovblyuk (Simferopol, Ukraine), Anton A. Nadolny (Sevastopol, Ukraine), Zoya Kastrygina (Simferopol, Ukraine) and Sergei L. Zonstein (Tel Aviv, Israel), etc. (Suppl. material 1: List 1).

**Table 2.** Authorship of Araneae taxa described by Yuri M. Marusik, sorted in decreasing order (percentages of total count in parentheses).

Author	Taxa	Author	Taxa
Marusik, Y. M.			
sole author	69 (8.85)	Magalhaes, I. L. F.	3 (0.38)
coauthor	711 (91.15)	Sidorov, V. V.	3 (0.38)
first author	236 (30.26)	Özkütük, R. S.	3 (0.38)
last author	401 (51.41)	Šestáková, A.	3 (0.38)
Zamani, A.	126 (16.15)	Buckle, D. J.	2 (0.26)
Logunov, D. V.	118 (15.13)	Cutler, B.	2 (0.26)
Koponen, S.	110 (14.10)	Leech, R.	2 (0.26)
Omelko, M. M.	68 (8.72)	Lehtinen, P. T.	2 (0.26)
Eskov, K. Y.	55 (7.05)	Ma, S. C.	2 (0.26)
Fomichev, A. A.	55 (7.05)	Stockmann, M.	2 (0.26)
Zonstein, S. L.	43 (5.51)	Sun, N.	2 (0.26)
Ovtsharenko, V. I.	41 (5.26)	Zhang, F.	2 (0.26)
Platnick, N. I.	38 (4.87)	Zyuzin, A. A.	2 (0.26)
Saaristo, M. I.	35 (4.49)	Aliabadian, M.	1 (0.13)
Azarkina, G. N.	28 (3.59)	Berry, J. W.	1 (0.13)
Esyunin, S. L.	23 (2.95)	Blagoev, G.	1 (0.13)
Guseinov, E.	22 (2.82)	Chevrizov, B. P.	1 (0.13)
Chatzaki, M.	20 (2.56)	Grabolle, A.	1 (0.13)
Kovblyuk, M. M.	13 (1.67)	Japoshvili, G.	1 (0.13)
Mirshamsi, O.	13 (1.67)	Karakaş, G.	1 (0.13)
Hippa, H.	12 (1.54)	Kaya, R. S.	1 (0.13)
Rakov, S. Y.	12 (1.54)	Komisarenko, A. A.	1 (0.13)
Fet, V.	11 (1.41)	Larsen, N.	1 (0.13)
Ballarin, F.	10 (1.28)	Lin, Y. J.	1 (0.13)
Penney, D.	10 (1.28)	Malek-Hosseini, M. J.	1 (0.13)
Gnelitsa, V. A.	8 (1.03)	Moradmand, M.	1 (0.13)
Kunt, K. B.	6 (0.77)	Mozaffarian, F.	1 (0.13)
Tanasevitch, A. V.	6 (0.77)	Nekhaeva, A. A.	1 (0.13)
Li, S. Q.	5 (0.64)	Ono, H.	1 (0.13)
Nadolny, A. A.	5 (0.64)	Otto, S.	1 (0.13)
Ponomarev, A. V.	5 (0.64)	Shafaie, S.	1 (0.13)
Zhang, X. Q.	5 (0.64)	Tarabaev, C. K.	1 (0.13)
Danilov, S. N.	4 (0.51)	Trilikauskas, L. A.	1 (0.13)
Kronestedt, T.	4 (0.51)	Tsellarius, A. Y.	1 (0.13)
Lyle, R.	4 (0.51)	Tuneva, T. K.	1 (0.13)
Tu, L. H.	4 (0.51)	Uğurtaş, İ. H.	1 (0.13)
Zheng, G.	4 (0.51)	Vahtera, V.	1 (0.13)
Elverici, M.	3 (0.38)	Yağmur, E. A.	1 (0.13)
Fritzén, N. R.	3 (0.38)	Perkovsky, E. E.	1 (0.13)
Kastrygina, Z. A.	3 (0.38)		

Yuri has never officially had his own students, but during the last 20 years he has helped many Ph.D. candidates, undergraduate students and young colleagues make their first steps in spider taxonomy. These include Elchin F. Guseinov (Huseynov, 8 joint papers), Galina N. Azarkina (6), Mykola M. Kovblyuk (19), Anton A. Nadolny (11), Alexander A. Fomichev (35), Alireza Zamani (40) and Mikhail M. Omelko (78). More than half of Yuri’s papers published during last 20 years are co-authored with young colleagues. These collaborations have allowed him to double the number of publications during last 10 years (Suppl. material 1: List 1).

## ﻿Editing and reviewing

Yuri is a member of the editorial board of several journals, including “Arthropoda Selecta”, “ZooKeys”, “Zoology in the Middle East”, “Acta Zoologica Bulgarica”, “Zoosystematica Rossica”, “Acta Arachnologica Sinica” and “Acta Biologica Sibirica.” At the end of 1990s, Yuri financially supported “Arthropoda Selecta”, a journal operating almost exclusively at the publisher’s expense.

Yuri is one of most active subject editors and reviewers of the above-mentioned journals. For example, for “ZooKeys”, Yuri has the most manuscript reviews and ranks as the 18^th^ most active editor. He edited 18 manuscripts and reviewed 49 in 2020 and edited and reviewed 27 and 53 manuscripts, respectively, in 2021.

## ﻿Final words

The authors would like to thank Yuri Mikhailovich Marusik for his contributions, kindness and help he has provided to colleagues. We congratulate him on the occasion of his 60^th^ birthday and wish him more achievements in his titanic arachnological efforts, helping to produce a new generation of arachnologists and enjoying his life with his family!

## Supplementary materials

Supplementary material 1 Lists 1–3 Data type PDF file Explanation note List 1 Publication list of Yuri M. Marusik, Institute for Biological Problems of the North RAS – as of March 28, 2022. 1A: Papers or books; 1B: Anniversaries and celebrations; 1C: Obituaries. List 2. List of the taxa described by Yuri M. Marusik. List 3. List of taxa named after Yuri M. Marusik. https://binary.pensoft.net/file/683286This dataset is made available under the Open Database License (http://opendatacommons.org/licenses/odbl/1.0/). The Open Database License (ODbL) is a license agreement intended to allow users to freely share, modify, and use this Dataset while maintaining this same freedom for others, provided that the original source and author(s) are credited.Jianshuang Zhang, Kirill G. Mikhailov, Seppo Koponen, Feiyang Long, Hao Yu, Shuqiang Li
