# Supplementary material for: ﻿Forty-two years of scientific research on eight legs—celebrating the 60th birthday of Dr Yuri M. Marusik
Source: Zookeys. 2022 May 13;1100:87–101. doi: 10.3897/zookeys.1100.85374 (PMC9848612; doi:10.3897/zookeys.1100.85374)
Supplement: Supplementary material 1 — Lists 1–3 [file zookeys-1100-087_article-85374__-s001.pdf]

List 1 Publication list of Yuri M. Marusik, Institute for Biological Problems of the North RAS – as of March 28, 2022

List 1A: Papers or books (References are arranged in alphanbetic order according to name and year of publication):

1. Alsos IG, Gillespie L, Marusik YM (2009) Arctic Islands, biology. In: Gillespie RG, Clague DA (Eds) Encyclopedia of Islands Encyclopedias of the Natural World. University of California Press, Auckland, 47–55.
2. Azarkina GN, Esyunin SL, Kuzmin EA, Marusik YM (2016) On the synonymy of two wolf spider species of the genus *Alopecosa* (Araneae, Lycosidae) from the steppe zone of Russia. Zootaxa 4205(4): 339–348. doi:10.11646/zootaxa.4205.4.2
3. Azarkina GN, Marusik YM, Antonenko TV (2015) First description of the male of *Alopecosa azsheganovae* Esyunin, 1996 (Araneae: Lycosidae). Zootaxa 4033(2): 265–269. doi:10.11646/zootaxa.4033.2.5
4. Babenko A, Behan-Pelletier V, Böcher J, Boxshall G, Brodo F, Coulson SJ, De Smet W, Dózsa-Farkas K, Elias S, Fjellberg A, Fochetti R, Foottit R, Hessen D, Hobaek A, Holmstrup M, Koponen S, Liston A, Makarova O, Marusik YM, Michelsen V, Mikkola K, Mustonen T, Pont A , Renaud A, Rueda LM, Savage J, Smith H, Samchyshyna L, Velle G, Viehberg F, Vikberg V, Wall H, Weider LJ, Wetterich S, Yu Q, Zinovjev A (2013) Terrestrial and freshwater invertebrates. In: Meltofte H (Ed.) Arctic Biodiversity Assessment. Status and trends in Arctic biodiversity. Conservation of Arctic Flora and Fauna, Akureyri, 194–223.
5. Ballarin F, Marusik YM, Omelko MM, Koponen S (2012) On the *Pardosa monticola* species-group (Araneae: Lycosidae) from middle Asia. Arthropoda Selecta 21(2): 161–182. doi:10.15298/arthsel.21.2.05
6. Berman DI, Gorbunov PY, Kononenko VS, Korotyaev BA, Marusik YM (2008) Invertebrates. Supplemental list. Red data book of Magadan Area. Rare and endangered species of plants and animals. Magadan: OOO "Upravlyayushchaya kompania "Staryi Gorod", 241–247. [In Russian]

7. Berman DI, Gorbunov PY, Kononenko VS, Korotyaev BA, Marusik YM, Khrulyova OA (2008) Invertebrates. Supplemental list. Red data book of Chukotsky Autonomous District. Vol.1. Animals. Magadan: Wild North. 228–234. [in Russian]
8. Berman DI, Marusik YM (1994) On *Bimastos parvus* (Oligochaeta: Lumbricidae) from Yukon Territory (Canada), with discussion of distribution of the earthworms in Northeastern North America and Northeastern Siberia. Megadrilogica 5(10): 113–116.
9. Blick T, Marusik YM (2018) Three junior synonyms of jumping spider genera (Araneae: Salticidae). Arthropoda Selecta 27(3): 237–238. doi:10.15298/arthsel.27.3.07
10. Crawford RL, Marusik YM (2006) Harvestmen (Arachnida: Phalangida or Opiliones) of Moneron Island. - Flora and fauna of Moneron Island (Materials of International Sakhalin Island Project). Dalnauka, Vladivostok, 96–201.
11. Dondale CD, Redner JH, Marusik YM (1997) Spiders (Araneae) of the Yukon. In: Danks HV (Ed.) Insects of the Yukon, Ottawa: 73–113.
12. Dudko RY, Lyubechanski II, Dubatolov VV, Marusik YM (2012) Siberian rock gardens. Nauka iz pervykh ruk N.2(44): 106–123. [in Russian]
13. Dunlop JA, Marusik YM, Vlaskin AP (2019) Comparing Arachnids in Rovno Amber with the Baltic and Bitterfeld Deposits. Paleontological Journal 53(10), 1074–1083. doi: 10.1134/S0031030119100034
14. Eskov KY, Marusik YM (1991) New linyphiid spider (Aranei, Linyphiidae) from east Siberia. Korean Arachnology 6(2): 237–253.
15. Eskov KY, Marusik YM (1991) On *Tunagyna* and *Thaleria*, two closely related Siberio-Nearctic spider genera (Araneida: Linyphiidae). Entomologica Scandinavica 22(4): 405–416. doi: 10.1163/187631291X00200 [publ. in Jan. 1992]
16. Eskov KY, Marusik YM (1992) On fossil spiders of the family Nesticidae (Chelicerata, Araneida). Palaeontologicheskii Zhurnal 2: 87–95. [In Russian]
17. Eskov KY, Marusik YM (1992) On the mainly Siberian spider genera *Wubanoides*, *Parawubanoides* gen.n. and *Poeciloneta* (Aranei Linyphiidae). Arthropoda Selecta

1(1): 21–38.

18. Eskov KY, Marusik YM (1992) On the Siberio-Nearctic erigonine spider genus *Silometopoides* (Araneida: Linyphiidae). Reichenbachia 29(19): 97–103.
19. Eskov KY, Marusik YM (1992) The spider genus *Centromerus* (Aranei Linyphiidae) in the fauna of Siberia and the Russian Far East, with an analysis of its distribution. Arthropoda Selecta 1(2): 33–46.
20. Eskov KY, Marusik YM (1994) New data on the taxonomy and faunistics of North Asian linyphiid spiders (Aranei Linyphiidae). Arthropoda Selecta 2(4): 41–79.
21. Eskov KY, Marusik YM (1995) On the spiders from Saur Mt. range, eastern Kazakhstan (Arachnida: Araneae). Beiträge zur Araneologie 4(1994): 55–94.
22. Eskov KY, Marusik YM (1997) A new species of erigonine spider genus *Erigonoplus* (Aranei Linyphiidae) from south Siberia. Arthropoda Selecta 6(1–2): 91–93.
23. Eskov KY, Marusik YM (2021) *Glebych minutissimus* gen. et sp. nov., the smallest cobweb spider (Araneae: Theridiidae). Zootaxa 5006(1): 45–53. doi:10.11646/zootaxa.5006.1.8
24. Esyunin SL, Marusik YM (2001) A new species of the genus *Devade* Simon, 1884 from Mongolia, with notes on *D. tenella* (Tyshchenko, 1965) (Aranei: Dictynidae). Arthropoda Selecta 9(2): 129–131.
25. Esyunin SL, Marusik YM (2011) Experience of the classification of the areas of distribution for the Ural's spiders. Vestnik Permskogo Universiteta. Biologia 1: 32–36. [In Russian]
26. Fedoriak MM, Rudenko CC, Marusik YM, Brushnivska (2010). Herpetobiont spiders of Chernivtsi city parks. Zavovidna Sprava v Ukraini 16(1): 64–71.
27. Fomichev AA, Marusik YM (2011) First description of the female of *Acantholycosa logunovi* (Araneae: Lycosidae). Zootaxa 2813: 65–68. doi:10.11646/zootaxa.2813.1.3
28. Fomichev, AA, Marusik YM (2011) New data on spiders (Arachnida: Aranei) of the Altai Republic, Russia. Arthropoda Selecta 20(2): 117–123. doi:10.15298/arthsel.20.2.03

29. Fomichev AA, Marusik YM (2013) New data on spiders (Arachnida: Aranei) of east Kazakhstan. Arthropoda Selecta 22(1): 83–92. doi:10.15298/arthsel.22.1.08
30. Fomichev AA, Marusik YM (2015) A survey of East Palaearctic Gnaphosidae (Araneae). 4. A review of *Fedotovia* Charitonov, 1946. Zootaxa 3948(1): 93–108. doi:10.11646/zootaxa.3948.1.6
31. Fomichev AA, Marusik YM (2015) First description of the male of *Drassodes kaszabi* (Aranei, Gnaphosidae). Vestnik Zoologii 49(2): 467–470. doi:10.1515/vzoo–2015–0055
32. Fomichev AA, Marusik YM (2017) A survey of East Palaearctic Gnaphosidae (Araneae). 8. New data on *Berlandina* and *Gnaphosa* from Mongolia. Zootaxa 4258(1): 69–80. doi:10.11646/zootaxa.4258.1.5
33. Fomichev AA, Marusik YM (2017) A survey of East Palaearctic Lycosidae (Araneae). 12. Two new *Mongolicosa* species from Mongolia. Zootaxa 4221(2): 233–241. doi: 10.11646/zootaxa.4221.2.7
34. Fomichev AA, Marusik YM (2017) A survey of East Palaearctic Lycosidae (Araneae). 13. A new genus of spiny-legs Pardosinae from Eastern Kazakhstan. Zootaxa 4320(2): 339–350. doi:10.11646/zootaxa.4320.2.8
35. Fomichev AA, Marusik YM (2017) *Gnaphosa serzonshteini*, a replacement name for *Gnaphosa zonsteini* Fomichev & Marusik, 2017 (Araneae: Gnaphosidae). Zootaxa 4286(3): 431. doi:10.11646/zootaxa.4286.3.10
36. Fomichev AA, Marusik YM (2018) Five new species of the *Acantholycosa*-complex (Araneae: Lycosidae) from Mongolia. Zootaxa 4497(2): 271–284. doi:10.11646/zootaxa.4497.2.7
37. Fomichev AA, Marusik YM (2019) A new species of the genus *Berlandina* (Aranei: Gnaphosidae) from Kazakhstan. Far Eastern Entomologist 390: 13–18. doi:10.25221/fee.390.2
38. Fomichev AA, Marusik YM (2019) A new species of *Lachesana* Strand, 1932 (Aranei: Zodariidae) from southern Kazakhstan. Arthropoda Selecta 28(4): 556–561. doi:10.15298/arthsel.28.4.08
39. Fomichev AA, Marusik YM (2020) Notes on the spider genus *Segestria* Latreille,

1804 (Araneae: Segestriidae) in the East Palaearctic with description of three new species. Zootaxa 4758(2): 330–346. doi:10.11646/zootaxa.4758.2.7

40. Fomichev AA, Marusik YM (2020) Redescription and new data on the distribution of *Scyloxes asiatica* Dunin 1992 (Aranei: Scytodidae) from Tajikistan. Acta Arachnologica 69(1): 43–48. doi:10.2476/asjaa.69.43
41. Fomichev AA, Marusik YM (2020) The first record of the spider subfamily Urocteinae Thorell, 1869 (Aranei: Oecobiidae) in Tajikistan. Arthropoda Selecta 29(2): 235–238. doi:10.15298/arthsel.29.2.08
42. Fomichev AA, Marusik YM (2021) A survey of East Palaearctic Gnaphosidae (Araneae). 11. New data on Gnaphosidae (Araneae) from Tajikistan. Zootaxa 4966(4): 443–457. doi:10.11646/zootaxa.4966.4.3
43. Fomichev AA, Marusik YM (2021) Notes on the spider genus *Dysdera* Latreille, 1804 (Araneae: Dysderidae) in Central Asia. Zootaxa 5006(1): 73–89. doi:10.11646/zootaxa.5006.1.10
44. Fomichev AA, Marusik YM, Koponen S (2014) A new species of *Xysticus* C.L. Koch, 1835 (Aranei: Thomisidae) from South Siberia. Arthropoda Selecta 23(2): 127–134. doi:10.15298/arthsel.23.2.04
45. Fomichev AA, Marusik YM, Koponen S (2016) On the synonymy of two *Acantholycosa* species (Araneae, Lycosidae) from the Altai. ZooKeys 559: 151–156. doi:10.3897/zookeys.559.7048
46. Fomichev AA, Marusik YM, Koponen S (2018) New data on spiders (Arachnida: Araneae) of Iraq. Zoology in the Middle East 64 (4): 329–339. doi: 10.1080/09397140.2018.1484018
47. Fomichev AA, Marusik YM, Omelko MM (2013) A new species of *Gnaphosa* Latreille, 1804 (Aranei: Gnaphosidae) from western Mongolia. Arthropoda Selecta 22(2): 153–156. doi:10.15298/arthsel.22.2.04
48. Fomichev AA, Marusik YM, Sidorov VV (2018) A survey of East Palaearctic Gnaphosidae (Aranei). 9. New data on the *Parasyrisca potanini*-group from Central Asia. Arthropoda Selecta 27(2): 155–168. doi:10.15298/arthsel.27.2.07
49. Gerlach J, Marusik YM (2010) Arachnida, Myriapoda of the Seychelles islands.

Siri Scientific Press, Rochdale, 435 pp.

50. Ghahari H, Marusik YM (2009) New data on spider fauna of Iran (Araneae). Turkish Journal of Arachnology 2(3): 1–8.
51. Gillespie MAK, Alfredsson M, Barrio IC, Bowden J, Convey P, Coulson SJ, Culler LE, Dahl MT, Daly KM, Koponen S, Loboda S, Marusik YM, Sandström JP, Sikes DS, Slowik J, Høye TT (2020) Circumpolar terrestrial arthropod monitoring: A review of ongoing activities, opportunities and challenges, with a focus on spiders. Ambio 49(3), 704–717. doi: 10.1007/s13280-019-01185-y
52. Gnelitsa VA, Marusik YM (2006) Redescription of the monotypic erigonine genus *Oculocornia* Oliger, 1985 from the Russian Far East (Araneae, Linyphiidae). Zootaxa 1344: 63–68. doi:10.11646/zootaxa.1344.1.6
53. Guseinov EF, Marusik YM, Koponen S (2005) Spiders (Arachnida: Aranei) of Azerbaijan 5. Faunistic review of the funnel-web spiders (Agelenidae) with the description of a new genus and species. Arthropoda Selecta 14(2): 153–177.
54. Haddad CR, Marusik YM (2019) Clarifying the taxonomic status and distributions of the spider species collected during the Leonhard Schultze expeditions in western and central southern Africa (Arachnida: Araneae). Zootaxa 4608(3): 451–483. doi:10.11646/zootaxa.4608.3.3
55. Huseynov EF, Marusik YM (2008) Spiders (Arachnida, Aranei) of Azerbaijan 3. Survey of the genus *Enoplognatha* Pavesi, 1880 (Theridiidae). Arthropoda Selecta 16(3): 153–167. [Huseynov: see Guseinov for earlier works]
56. Ivanov V, Marusik YM, Pétillon J, Mutanen M (2021) Relevance of ddRADseq method for species and population delimitation of closely related and widely distributed wolf spiders (Araneae, Lycosidae). Scientific Reports 11(1): 2177. doi: 10.1038/s41598-021-81788-2
57. Kanyukova EV, Marusik YM (2006) A checklist of Heteroptera of the Kuril Islands and brief zoogeographical survey of the fauna. Biodiversity and Biogeography of the Kuril Islands and Sakhalin, Bulletin of the Hokkaido University Museum 3: 161–174.
58. Kaya RS, Kunt KB, Marusik YM, Uğurtaş İH (2010) A new species

of *Tegenaria* Latreille, 1804 (Araneae, Agelenidae) from Turkey. ZooKeys 51: 1–16. doi:10.3897/zookeys.51.467

59. Kaya RS, Kunt KB, Marusik YM, Yağmur EA (2010) The first record of genus *Argyrodes* Simon, 1864 (Araneae: Theridiidae) from Turkey. Serket 12(1): 7–12.
60. Kerzhner IM, Kanyukova EV, Marusik YM, Urbain BK, Nakamura M, Lelei AS (2004) Heteroptera of the Kuril Islands: material collected by the International Expedition 1994-1999 and updated checklist. Zoosystematica Rossica 12(2): 231–242.
61. Kerzhner IM, Marusik YM (1997) Heteroptera (Insecta) of the Kurile Islands: revised checklist and new data on Middle Kuriles.Russian Entomological Journal 5(1–4): 21–29.
62. Kioko GM, Marusik YM, Li SQ, Kioko EN, Ji LQ (2021) Checklist of the spiders (Araneae) of Kenya. African Invertebrates 62(1): 49–229. doi:10.3897/AfrInvertebr.62.58776
63. Komisarenko AA, Omelko MM, Marusik YM (2019) An annotated list of linyphiid spiders (Aranei: Linyphiidae) of Laos. Far Eastern Entomologist 377: 26–32. doi:10.25221/fee.377.2
64. Koponen S, Hoffmann J, Marusik YM (2002). *Kikimora palustris* Eskov, 1988 (Araneae: Linyphiidae) found in Europe. Entomologica Fennica 13: 129–133. doi: 10.33338/ef.84151
65. Koponen S, Marusik YM (1992) Spiders (Araneae) from Central Yakutia, Siberia. Entomologica Fennica 3(3): 163–166. doi: 10.33338/ef.83608
66. Koponen S, Marusik YM, Potapova NK (2004) Spiders (Araneae) from the Lena and Yana Rivers, Yakutia (Sakha Republic). Entomologica Fennica 15: 113–118. doi: 10.33338/ef.84214
67. Koponen S, Marusik YM, Tanasevitch AV 1997(1998). New data on the spider fauna of the Polar Urals (Aranei). Arthropoda Selecta 6(3–4): 109–119.
68. Kovblyuk MM, Kastrygina ZA, Marusik YM (2013) A new genus *Shaitan elchini* gen. et sp.n. (Aranei: Gnaphosidae) from Azerbaijan and

Kazakhstan. Arthropoda Selecta 22(2): 145–151. doi:10.15298/arthsel.22.2.03

69. Kovblyuk MM, Kastrygina ZA, Marusik YM (2017) New data on *Hahnia* C.L. Koch, 1841 from Crimea (Aranei: Hahniidae). Turkish Journal of Zoology 41(2): 311–317. doi: 10.3906/zoo-1603-55

70. Kovblyuk MM, Kastrygina ZA, Marusik YM, Omelko MM (2013). Redescription of the spider *Haplodrassus caspius* Ponomarev & Belosludtsev, 2008, with the first description of the male (Araneae: Gnaphosidae). Zoology in the Middle East 59: 66–69. doi:10.1080/09397140.2013.795069

71. Kovblyuk MM, Kastrygina ZA, Marusik YM, Ponomarev AV (2013). The spider genus *Pireneitega* Kishida, 1955 in the Caucasus (Aranei: Agelenidae: Coelotinae). Arthropoda Selecta 22(1): 59–73. doi:10.15298/arthsel.22.1.06

72. Kovblyuk MM, Marusik YM (2012) First description of the female of the theridiid spider *Robertus golovatchi* (Araneae: Theridiidae). Arachnologische Mitteilungen 44: 17–19. doi:10.5431/aramit4405

73. Kovblyuk MM, Marusik YM, Omelko MM (2012) A survey of Transcaucasian *Dipoena* sensu lato (Aranei: Theridiidae) with a description of new species. Arthropoda Selecta 21(3): 247–254. doi:10.15298/arthsel.21.3.07

74. Kovblyuk MM, Marusik YM, Omelko MM (2013) On four poorly known species of spiders (Araneae: Gnaphosidae and Lycosidae) described by T. Thorell from Crimea. Acta Zoologica Bulgarica 65(4): 423–427.

75. Kovblyuk MM, Marusik YM, Ponomarev AV, Gnelitsa VA, Nadolny AA (2011) Spiders (Arachnida: Aranei) of Abkhazia. Arthropoda Selecta 20(1): 21–56. doi:10.15298/arthsel.20.1.03

76. Kovblyuk MM, Otto S, Marusik YM, Ponomarev AV (2012) Redescription of the Caucasian species *Geolycosa charitonovi* (Mcheidze, 1997) (Araneae: Lycosidae), with the first description of the male. Bulletin of the British Arachnological Society 15(8): 245–252. doi:10.13156/arac.2012.15.1.245

77. Kozlov MV, Oudendijk Z, Forsman A, Lanta V, Barclay MVL, Gusarov VI, Gustafsson B, Huang ZZ, Kruglova OY, Marusik YM, Mikhailov YE, Mutanen M, Schneider A, Sekerka L, Sergeev ME, Zverev V, Zvereva EL (2021) Climate

shapes the spatiotemporal variation in color morph diversity and composition across the distribution range of *Chrysomela lapponica* leaf beetle. Insect Science (2021) (0): 1–14. doi: 10.1111/1744-7917.12966

78. Kronestedt T, Marusik YM (2002) On *Acantholycosa solituda* (Levi & Levi) and *A. sterneri* (Marusik) (Araneae: Lycosidae), a pair of geographically distant allied species. Acta Arachnologica 51(1): 63–71. doi: 10.2476/asjaa.51.63
79. Kronestedt T, Marusik YM (2011) Studies on species of Holarctic *Pardosa* groups (Araneae, Lycosidae). VII. The *Pardosa tesquorum* group. Zootaxa 3131: 1–34. doi:10.11646/zootaxa.3131.1.1
80. Kronestedt T, Marusik YM, Omelko MM (2014) Studies on species of Holarctic *Pardosa* groups (Araneae, Lycosidae). VIII. The Palearctic species of the *Pardosa nigra* group. Zootaxa 3894(1): 33–60. doi:10.11646/zootaxa.3894.1.5
81. Kropf C, Blick T, Brescovit AD, Chatzaki M, Dupérré N, Gloor D, Haddad CR, Harvey MS, Jäger P, Marusik YM, Ono H, Rheims CA, Nentwi W (2019) How not to delimit taxa: a critique on a recently proposed "pragmatic classification" of jumping spiders (Arthropoda: Arachnida: Araneae: Salticidae). Zootaxa 4545(3): 444–446. doi:10.11646/zootaxa.4545.3.10
82. Kunt KB, Marusik YM, Omelko MM (2013) A review of the genus *Hygrocrates* with a description of a new species from Turkey (Araneae: Dysderidae). Acta Zoologica Bulgarica 65(2): 273–277.
83. Kunt KB, Özkütük RS, Elverici M, Marusik YM, Karakaş G (2016) *Harpactea karaschkhan* sp. n., a new cave-dwelling blind spider species from the Mediterranean region of Turkey. Journal of Cave and Karst Studies 78(1): 36–40. doi:10.4311/2015LSC0106
84. Kurenshchikov DK, Marusik YM (2009) Spiders of the "Bolshekhekhtsyrski" Reserve: taxonomic diversity, zoogeographical status. IIIrd Druzyninskiye Chteniya. Komlexnyye Issledovaniya prirodnoy sredy v basseine reki Amur. Materialy mezhregionalnoi nauchnoi konferentsii 6-9 oktyabrya. Khabarovsk, 208-211. [In Russian]
85. Lasut L, Marusik YM, Frick H (2009) First description of the female of the

spider *Savignia zero* Eskov, 1988 (Araneae: Linyphiidae). Zootaxa 2267: 65–68. doi:10.11646/zootaxa.2267.1.5

86. Lehtinen PT, Marusik YM (2008) A redefinition of *Misumenops* F. O. Pickard-Cambridge, 1900 (Araneae, Thomisidae) and review of the New World species. Bulletin of the British Arachnological Society 14(4): 173–198.
87. Lin YJ, Marusik YM, Gao CX, Xu H, Zhang XQ, Wang ZY, Zhu WH, Li SQ (2021) Twenty-three new spider species (Arachnida: Araneae) from Asia. Zoological Systematics 46(2): 91–152. doi:10.11865/zs.2021201
88. Liu JX, Xu X, Marusik YM, Yin HQ (2021) Taxonomic notes on a pirate spider occurring in China (Araneae, Mimetidae). Zootaxa 4974(3): 565–576. doi:10.11646/zootaxa.4974.3.5
89. Logunov DV, Ballarin F, Marusik YM (2011) New faunistic records of the jumping and crab spiders of Karakoram, Pakistan (Aranei: Philodromidae, Salticidae and Thomisidae). Arthropoda Selecta 20(3): 233–240. doi:10.15298/arthsel.20.3.06
90. Logunov DV, Cutler B, Marusik YM (1993) A review of the genus *Euophrys* C. L. Koch in Siberia and the Russian Far East (Araneae: Salticidae). Annales Zoologici Fennici 30(2): 101–124.
91. Logunov DV, Marusik YM (1990) The spider genus *Argyrodes* (Aranei, Theridiidae) in the USSR. Zoologicheskiĭ Zhurnal 69(2): 133–136. [In Russian]
92. Logunov DV, Marusik YM (1990) The spider genus *Phoroncidia* (Aranei, Theridiidae) in the USSR. Proceeding of the Zoological Institute of the Academy of Sciences of USS, Leningrad, 226: 91–97. [publ. in 1992] [In Russian]
93. Logunov DV, Marusik YM (1991) Redescriptions and morphological differences of *Bianor aurocinctus* (Ohlert) and *B. aemulus* (Gertsch) (Aranei, Salticidae). Sibirskij Biologichesky Zhurnal 2: 39–47. [In Russian]
94. Logunov DV, Marusik YM (1994) A faunistic review of the crab spiders (Araneae, Thomisidae) from the mountains of south Siberia. Bulletin de l'Institut Royal des Sciences Naturelles de Belgique, Entomologie, 64: 177–197.
95. Logunov DV, Marusik YM (1994) New data on the jumping spiders of the Palearctic fauna (Aranei Salticidae). Arthropoda Selecta 3(1–2): 101–115.

96. Logunov DV, Marusik YM (1995) Spiders of the family Lycosidae (Aranei) from the Sokhondo Reserve (Chita area, east Siberia). Beiträge zur Araneologie 4(1994): 109–122. [publ. in Dec. 1995]
97. Logunov DV, Marusik YM (1998) A new species of the genus *Xysticus* from the mountains of south Siberia and Mongolia (Araneae, Thomisidae). Bulletin of the British Arachnological Society 11(3): 103–106
98. Logunov DV, Marusik YM (1999) A brief review of the genus *Chalcoscirtus* Bertkau, 1880 in the faunas of Central Asia and the Caucasus (Aranei: Salticidae). Arthropoda Selecta 7(3): 205–226.
99. Logunov DV, Marusik YM (1999) New species and new records of the jumping spiders from the Russian Far East (Araneae, Salticidae). Acta Arachnologica 48(1): 23–29. doi: 10.2476/asjaa.48.23
100. Logunov DV, Marusik YM (2000) Miscellaneous notes on Palaearctic Salticidae (Arachnida: Aranei). Arthropoda Selecta 8(4, 1999): 263–292.
101. Logunov DV, Marusik YM (2001) Catalogue of the jumping spiders of northern Asia (Arachnida, Araneae, Salticidae). KMK Scientific Press, Moscow, 300 pp.
102. Logunov DV, Marusik YM (2003) A revision of the genus *Yllenus* Simon, 1868 (Arachnida, Araneae, Salticidae). KMK Scientific Press, Moscow: 167 pp.
103. Logunov DV, Marusik YM (2003) Taxonomic and faunistic notes on *Chinoscopus* Simon, 1900 and *Lyssomanes* Hentz, 1845 from the Neotropical region (Araneae, Salticidae). Bulletin of the British Arachnological Society 12(9): 415–424.
104. Logunov DV, Marusik YM (2004) Order Araneae-spiders. Biodiversity of the Sokhondo Nature Reserve. Arthropoda. Novosibirsk-Chita: 41–80. [in Russian]
105. Logunov DV, Marusik YM (2014) Taxonomic notes on the genus *Eupoa* Żabka, 1985 (Arachnida, Araneae, Salticidae). ZooKeys 410: 63–93. Link doi:10.3897/zookeys.410.7548
106. Logunov DV, Marusik YM, Koponen S (1998) A check-list of the spiders in Tuva, South Siberia with analysis of their habitat distribution. Berichte des Naturwissenschaftlich Medizinischen Vereins in Innsbruck, 85: 125–159
107. Logunov DV, Marusik YM, Koponen S (2002) Redescription of a poorly known

spider species, *Xysticus kulczynskii* Wierzbicki 1902 (Araneae: Thomisidae). Acta Arachnologica 51(2): 99–104. doi: 10.2476/asjaa.51.99

108. Logunov DV, Marusik YM, Mozaffarian F (2002) Faunistic review of the jumping spiders of Iran (Aranei: Salticidae). Arthropoda Selecta 10(2, 2001): 155–167.
109. Logunov DV, Marusik YM, Rakov SY (1999) A review of the genus *Pellenes* in the fauna of Central Asia and the Caucasus (Araneae, Salticidae). Journal of Natural History 33(1): 89–148. doi: 10.1080/002229399300489
110. Logunov DV, Marusik YM, Trilikauskas L A (2001) A new species of the genus *Xysticus* C. L. Koch from south Siberia (Arachnida: Araneae: Thomisidae). Reichenbachia 34(3): 33–38.
111. Magalhaes ILF, Stockmann M, Marusik YM, Zonstein SL (2020) On *Sahastata* (Araneae: Filistatidae): complementary description of the generotype and two new species from Oman and Morocco. Zootaxa 4899(1): 215–246. doi:10.11646/zootaxa.4899.1.12
112. Makarova OL, Anufriyev VV, Babenko AB, Bizin MS, Glazov RM, Kolesnikova AA, Marusik YM, Tatarinov AG (2018) Siberia in Europe: an analisis of the Nenets Autonomous Okrug fauna, Russian Arctic. In Khamenkova EV (Ed) Biological Problems of the North: The Materials of International Scientific Conference, dedicated to Academician V L Kontrimavichus, Magadan, 18–22 September 2018). Magadan, 345–349. [in Russian]
113. Makarova OL, Anufriyev VV, Babenko AB, Bizin MS, Glazov PM, Kolesnikova AA, Marusik YM, Tatarinov AG (2019) Fauna of the East European Tundra: the Input of "Siberian" Species. Bulletin of the North-East Science Center: 59–71. doi: 10.34078/1814-0998-2019-1-59-71 [in Russian]
114. Mąkol J, Marusik YM (1999) Trombidiidae (Acari: Actinotrichidae, Trombidioidea) of Kuril Islands. Part I. Podothrombidiidae Thor, 1935. Annales Zoologici (Warszawa) 49(3): 329–346.
115. Marikovskii PI, Marusik YM (1985) *Araneus pallasi* (Aranei: Araneidae)-social spider of the USSR fauna. Vestnik Leningradskogo Universiteta (Biol.) 17: 3–8. [In Russian]

116. Marusik YM (1984) To the spider fauna of "Les na Vorskle" Reserve. Fauna and ecology of arachnids, Perm, 132–138. [In Russian]
117. Marusik YM (1985) A systematic list of the orb-weaving spiders (Aranei: Araneidae, Tetragnathidae, Theridiosomatidae, Uloboridae) of the European part of the USSR and the Caucasus. Trudy Zoologicheskogo Instituta Akademii Nauk SSSR, Leningrad 139: 135–140. [In Russian]
118. Marusik YM (1985) Revision of the spider genus *Cercidia* Thorell (Aranei: Araneidae) with a description of a new species. Vestnik Leningradskogo Universiteta (Biol.) 1985(3): 114–118. [In Russian]
119. Marusik YM (1986) A redescription of types of certain orb-weaving spiders (Araneidae, Tetragnathidae) from S. A. Spassky collection. Vestnik Zoologii 1986(6): 19–22. [In Russian]
120. Marusik YM (1986) The orb-weaver genus *Larinia* Simon in the USSR (Aranei, Araneidae). Spixiana 9: 245–254.
121. Marusik YM (1987) Comparative study of the webs of the orb-weaving spiders (Aranei, Araneidae, Tetragnathidae, Uloboridae) from Lagodekhi reserve. Vestnik Zoologii 3: 83–86. [In Russian]
122. Marusik YM (1987) Systematics and biology of the orb-weaving spider, *Octonoba yesoensis* (Aranei, Uloboridae). Zoologicheskiĭ Zhurnal 66(4): 613–616. [In Russian]
123. Marusik YM (1987) Three new species of the family Nesticidae (Aranei) from the fauna of the USSR. Zoologicheskiĭ Zhurnal 66(3): 461–463. [In Russian]
124. Marusik YM (1988) New species of spiders (Aranei) from the Upper Kolyma. Zoologicheskiĭ Zhurnal 67(10): 1469–1482. [In Russian]
125. Marusik YM (1988). Three new spider species of the family Linyphiidae (Aranei) from the north-east of the USSR. Zoologicheskiĭ Zhurnal 67(12): 1914–1918. [In Russian]
126. Marusik YM (1989) New data on the fauna and synonymy of the USSR spiders (Arachnida, Aranei). In: Lange AB (ed.) Fauna i Ekologiy Paukov i Skorpionov: Arakhnologicheskii Sbornik. Akademia Nauk SSSR, Moscow, 39–52. [In Russian]

127. Marusik YM (1989). Two new species of the spider genus *Xysticus* and synonymy of crab spiders (Aranei, Thomisidae, Philodromidae) from Siberia. Zoologicheskiĭ Zhurnal 68(4): 140–145. [In Russian]
128. Marusik YM (1990) 14th annual meeting of American Arachnological Society.Proceeding of the Zoological Institute of the Academy of Sciences of USSR, 226: 142–144. [In Russian]
129. Marusik YM (1990) Spider genus *Chalcoscirtus* (Aranei, Salticidae) from the USSR: Communication 1. Zoologicheskiĭ Zhurnal 69(6): 45–57. [In Russian]
130. Marusik YM (1990) Zoogeographical affinities of the upper Kolyman spider fauna. Proceeding of the Zoological Institute of the Academy of Sciences of USSR, Leningrad, 226: 125–127. [publ. in 1992] [In Russian]
131. Marusik YM (1991). Crab spiders of the family Philodromidae (Aranei) from east Siberia. Zoologicheskiĭ Zhurnal 70(10): 48–58. [In Russian]
132. Marusik YM (1991) New data on spiders of the subfamily Linyphiinae (Aranei, Linyphiidae) of east Siberia. Zoologicheskiĭ Zhurnal 70(6): 61–68. [In Russian]
133. Marusik YM (1991) Spider genus *Chalcoscirtus* (Aranei, Salticidae) from the USSR. Communication 2. Zoologicheskiĭ Zhurnal 70(1): 19–31. [In Russian]
134. Marusik YM (1991) Spider genus *Chalcoscirtus* (Aranei, Salticidae) from the USSR. Communication 3. Zoologicheskiĭ Zhurnal 70(2): 22–29. [In Russian]
135. Marusik YM (1993) A check-list of spiders (Aranei) from the upper Kolyma. Multidisciplinary study in the Kontakt Field Station. Dalnauka Press, Vladivostok, 206-223. [In Russian]
136. Marusik YM (1993) Re-description of spiders of the families Heteropodidae and Thomisidae (Aranei), described by O. P.-Cambridge from the material of the second Yarkand mission. Entomologicheskoe Obozrenie 72(2): 456–467. [The english version was published in Entomological Review 74 (6): 100–113, 1995] [In Russian]
137. Marusik YM (1993) Terrestrial invertebrates. In: Berman DI (ed.). Ecology of the Amguema River basin (Chukotka). Vladivostok 1: 164–185. [In Russian]
138. Marusik YM (1993) Three new wandering spider species (Aranei Lycosidae

Gnaphosidae) from Mongolia. Arthropoda Selecta 2(1): 77–81.

139. Marusik YM (1994) A check-list of spiders with trans-Palaearctic distribution. Bollettino dell'Accademia Gioenia di Scienze Naturali 26(345): 273–279

140. Marusik YM (1995) A review of the spider genus *Titanoeca* from Siberia (Aranei: Titanoecidae). Beiträge zur Araneologie 4(1994): 123–132. [publ. in Dec. 1995]

141. Marusik YM (1996) A comparison of the spider faunas of Scandinavia and north-east Siberia. Acta Zoologica Fennica 201: 7–10.

142. Marusik YM (2000) Orchids cheating bees. Nature (Russian) 4: 75. [In Russian]

143. Marusik YM (2002) Earthworms (Oligohaeta) – Plants and animals of the Kurile Islands (Reports of International Kuril Islands Project). Dalnauka Press, Vladivostok: 67–68. [In Russian]

144. Marusik YM (2002) Spiders (Aranei) – Plants and animals of the Kurile Islands (Reports of International Kuril Islands Project). Dalnauka Press, Vladivostok: 69–73. [In Russian]

145. Marusik YM (2004) Fauna and populations of stone debris spiders (Arachnida, Aranei) of north-east Siberia and north-west Canada. Arthropoda Selecta (Special Issue №1, 2004): 185–200.

146. Marusik YM (2005) A new family and interesting new records of spiders (Aranei) from the European part of Russia. Arthropoda Selecta 14(1): 89–91.

147. Marusik YM (2005) Advances and perspectives in study of taxonomic diversity of arachnids (Arachnida: Aranei, Opiliones, Pseudoscorpiones) in northeastern Asia. Magadan: Northeastern Scientific Centre of Russian Academy of Sciences: 329–332. [in Russian]

148. Marusik YM (2005) Arachnids (Arachnida: Aranei, Opiliones) of northern Cisokhotia. Euroasian Entomological Journal 4(3): 187–208. [In Russian]

149. Marusik YM (2005) Spiders and harvestmen of the Taui Bay shore and adjacent parts of northern Cisokhotia. Biodiversity of Tauysk Bay of the Sea of Okhotsk: 262–289. [In Russian]

150. Marusik YM (2008) Synopsis of the *Ozyptila rauda*-group (Araneae, Thomisidae), with revalidation of *Ozyptila balkarica* Ovtsharenko, 1979. Zootaxa 1909: 52–64.

doi: 10.11646/zootaxa.1909.1.5

151. Marusik YM (2009) A check-list of spiders (Aranei) from the Lazo Reserve, Maritime Province, Russia. Arthropoda Selecta 18(1–2): 95–109.
152. Marusik YM (2009) Araneogeographic belonging of Greenland.Izvestiya Irkutskgo Gosudarstvennogo Universiteta. Seriya Biologia Ecologiya 2(2): 11–13. [in Russian]
153. Marusik YM (2009) *Araneus pallasi* (Thorell, 1875) (Araneae: Araneidae): A new species for Turkey. Turkish Journal of Arachnology 2(1): 1–3. [In Russian]
154. Marusik YM (2009) First description of the male of *Alloclubionoides amurensis* (Ovtchinnikov, 1999) (Aranei: Amaurobiidae). Arthropoda Selecta 18(1–2): 51–55.
155. Marusik YM (2009) First description of the male of *Prodidomus redikorzevi* Spassky, 1940 (Aranei: Prodidomidae). Acta Arachnologica 58(2): 59–63. doi:10.2476/asjaa.58.59
156. Marusik YM (2009) On central Asian *Castianeira arnoldii* Charitonov, 1946 (Araneae, Corinnidae), earlier known from juvenile specimens. Zootaxa 2226: 66–68. doi:10.11646/zootaxa.2226.1.6
157. Marusik YM (2009) On the northernmost species of Hersiliidae (Aranei), *Hersiliola xinjiangensis* (Liang & Wang, 1989), comb.n. Arthropoda Selecta 17(3–4): 153–156.
158. Marusik YM (2009) Order Aranei - Spiders. Insects of the Lazo Reserve. Dal'nauka, Vladivostok, 380–392. [In Russian]
159. Marusik YM (2009). Redescription of *Paccius quadridentatus* Simon, 1898 (Araneae: Corinnidae: Trachelinae) based on the holotype. Zootaxa 1990: 65–68. doi: 10.11646/zootaxa.1990.1.5
160. Marusik YM (2009) Spiders (Araneae) new to the fauna of Turkey. 4. New species record of *Anyphaena* (Anyphaenidae). Turkish Journal of Arachnology 2(4): 9–11.
161. Marusik YM (2009) Spiders (Araneae) new to the fauna of Turkey. 6. New species and genera records of Araneidae. Turkish Journal of Arachnology 2(4): 12–16.
162. Marusik YM (2009) The Yenisei zoogeographical boundary and distribution of

spiders in northern Asia. Izvestiya Irkutskgo Gosudarstvennogo Universiteta. Seriya Biologia Ecologiya 2(1): 49–52. [in Russian]

163. Marusik YM (2010). A new species of *Tetragnatha* Latreille, 1904 (Aranei: Tetragnathidae) from western Kazakhstan. Arthropoda Selecta 19(3): 199–202. doi:10.15298/arthsel.19.3.07
164. Marusik YM (2010) First description of the female of *Xerophaeus espoir* Platnick, 1981 (Aranei: Gnaphosidae). Arthropoda Selecta 19(1): 7–9. doi:10.15298/arthsel.19.1.02
165. Marusik YM (2011) Advances in the study of spiders (Aranei) of Russia and of the World. Entomological Review 4: 917–929. [in Russian]
166. Marusik YM (2011) A new genus of hahniid spiders from Far East Asia (Araneae: Hahniidae). Zootaxa 2788: 57–68. doi:10.11646/zootaxa.2788.1.4
167. Marusik YM (2011) A new *Trygetus* species from Central Asia (Araneae: Zodariidae). Turkish Journal of Zoology 35(1): 29–31. doi:10.3906/zoo–0907–78
168. Marusik YM (2012) Araneogeographic status of Beringia. Eurasian Entomological Journal 11(Suppl. 1): 41–54. [in Russian]
169. Marusik YM (2015). Araneae (Spiders). In: Böcher J, Kristensen NP, Pape T, Vilhelmsen L (eds.) The Greenland Entomofauna. An identification manual of insects, spiders and their allies. Brill, Leiden, Fauna Entomologica Scandinavica **44**: 667–703. doi:10.1163/9789004261051_019
170. Marusik YM (2016) Redescription of the male of *Pterotricha strandi* (Aranei: Gnaphosidae) with special emphasis on the structure of the palp. Arthropoda Selecta 25(3): 279–282. doi:10.15298/arthsel.25.3.07
171. Marusik YM (2017) A review of *Diphya* (Aranei: Tetragnathidae) from South Africa. Arthropoda Selecta 26(2): 133–138. doi:10.15298/arthsel.26.2.06
172. Marusik YM (2017) Description of unknown sexes in two *Pireneitega* species from Tajikistan (Araneae: Agelenidae, Coelotinae). Zootaxa 4273(3): 435–438. doi:10.11646/zootaxa.4273.3.8
173. Marusik YM (2017) On two sibling species of *Hahnia* (Araneae: Hahniidae) from Western Cape, South Africa. Arachnology 17(6): 299–

301. doi:10.13156/arac.2017.17.6.299

174. Marusik YM (2017) Redescription of *Dysdera cylindrica* (Aranei: Dysderidae) from Pakistan with notes on the distribution of the family. Arthropoda Selecta 26(4):310–314. doi: 10.15298/arthsel.26.4.05

175. Marusik YM (2017) Redescription of the holotype of *Larinia vara* Kauri, 1950 (Aranei: Araneidae) from South Africa, with establishing of a new combination. Arthropoda Selecta 26(3): 245–247. doi:10.15298/arthsel.26.3.06

176. Marusik YM (2017) Redescription of the type species of the genus *Argistes* (Aranei, Liocranidae). Vestnik Zoologii 51(5): 363–368. doi:10.1515/vzoo–2017–0043

177. Marusik YM (2018) Case 3774 – Diphyini Simon, 1894 (Arachnida, Araneae, Tetragnathidae): proposed emendation of the current spelling to Diphyaini to remove homonymy with Diphyidae Quoy & Gaimard, 1827 (Hydrozoa, Siphonophorae). Bulletin of Zoological Nomenclature 75: 170–172. doi:10.21805/bzn.v75.a034

178. Marusik YM (2018) Redescription of *Alopecosa albostriata* (Araneae: Lycosidae) based on specimens from Siberia. Zootaxa 4482(2): 383–391. doi:10.11646/zootaxa.4482.2.10

179. Marusik YM (2018) Redescription of the Siberian species *Pardosa jeniseica* (Araneae: Lycosidae). Zootaxa 4497(1): 141–144. doi:10.11646/zootaxa.4497.1.9

180. Marusik YM (2018) Supraspecific names in spider systematics and their nomenclatural problems. Arachnologische Mitteilungen: Arachnology Letters 55(1): 42–45. doi: 10.30963/aramit5507

181. Marusik YM (2018) The first record of *Micaria bonneti* (Aranei: Gnaphosidae) in Mongolia. Arthropoda Selecta 27(4): 335–338. doi:10.15298/arthsel.27.4.09

182. Marusik YM (2019) A new species of *Euophrys* (Aranei: Salticidae) from Israel. Arthropoda Selecta 28(4): 562–566. doi:10.15298/arthsel.28.4.09

183. Marusik YM (2021) Epigyne or epigynum: what is correct? Arthropoda Selecta 30(1): 130–132. doi: 10.15298/arthsel.30.1.13

184. Marusik YM, Alfimov AV (2012) Spiders (Arachnida, Aranei) and microclimate of xeromorphic habitats in the lower reaches of Kolyma River 69° N. Eurasian Entomological Journal 11(Suppl. 1): 27–40. [in Russian]
185. Marusik YM, Azarkina GN (2016) Redescription of the poorly known crab spider *Xysticus spasskyi* (Araneae: Thomisidae). *Zootaxa* 4161(4): 561–566. doi:10.11646/zootaxa.4161.4.7
186. Marusik YM, Azarkina GN (2020) Who is *Eresus tristis* Kroneberg, 1875 (Aranei: Eresidae)?. Arthropoda Selecta 29(4): 470–474. doi:10.15298/arthsel.29.4.09
187. Marusik YM, Azarkina GN, Koponen S (2004) A survey of east Palearctic Lycosidae (Aranei). II. Genus *Acantholycosa* F. Dahl, 1908 and related new genera. Arthropoda Selecta 12(2, 2003): 101–148. [publ. Feb. 28, 2004]
188. Marusik YM, Azarkina GN, Omelko MM (2015) Redescription and a new synonym of the spider *Pachygnatha amurensis* Strand, 1907 (Araneae, Tetragnathidae, Tetragnathinae). Zootaxa 4007(1): 126–130. doi: 10.11646/zootaxa.4007.1.10
189. Marusik YM, Ballarin F (2011) A new species of *Draconarius* Ovtchinnikov, 1999 (Araneae, Amaurobioidea, Coelotinae) from northern Pakistan. Zootaxa 2739: 27–32. doi:10.11646/zootaxa.2739.1.2
190. Marusik YM, Ballarin F (2011) Redescription of the Himalaian *Pardosa flavisterna* Caporiacco, 1935 (Aranei: Lycosidae) with notes of the *Pardosa nebulosa* species-group. Proceedings of the Zoological Institute of the Russian Academy of Sciences 315(1): 63–69.
191. Marusik YM, Ballarin F, Omelko MM (2012) On the *Pardosa monticola*-species group from Iran (Araneae: Lycosidae). Zoology in the Middle East 56: 111–123. doi:10.1080/09397140.2012.10648946
192. Marusik YM, Ballarin F, Omelko MM (2012) On the spider genus *Amaurobius* (Araneae, Amaurobiidae) in India and Nepal. ZooKeys 168: 55–64. doi:10.3897/zookeys.168.2352
193. Marusik YM, Ballarin F, Omelko MM, Koponen S (2014). On new and interesting records of spiders from northern Pakistan and India (Aranei). Arthropoda

Selecta 23(4): 415–424. doi:10.15298/arthsel.23.4.09

194. Marusik YM, Blagoderov V (2021) Kirill Glebovich Mikhailov: On the occasion of his 60th Birthday (Foreword). Zootaxa 5006(1): 5. doi: 10.11646/zootaxa.5006.1.3
195. Marusik YM, Blick T (2019) Further new synonyms of jumping spider genera (Araneae: Salticidae). Arachnologische Mitteilungen 57(1): 89–91. doi:10.30963/aramit5717
196. Marusik YM, Böcher J, Koponen S (2006) The collection of Greenland spiders (Aranei) kept in the Zoological Museum, University of Copenhagen. Arthropoda Selecta 15(1): 59–80.
197. Marusik YM, Buchar J (2004) A survey of the east Palearctic Lycosidae (Aranei). 3. On the wolf spiders collected in Mongolia by Z. Kaszab in 1966–1968. Arthropoda Selecta 12(2, 2003): 149–158. [publ. Feb. 28, 2004]
198. Marusik YM, Buckle DJ, Koponen S (2007) A survey of the Holarctic Linyphiidae (Araneae), a review of the erigonine genus *Zornella* Jackson, 1932. Acta Zootaxonomica Sinica 32(1): 21–34.
199. Marusik YM, Chevrizov BP (1990) Three new crab spiders from the Asian part of the USSR (Arachnida, Araneae: Thomisidae). Reichenbachia 27(15): 89–93.
200. Marusik YM, Crawford RL (2006) Spiders (Aranei) of Moneron Island. In: Flora and Fauna of Moneron Island. Dalnauka, Vladivostok, 171–195. [In Russian]
201. Marusik YM, Cutler B (1989) Descriptions of the males of *Dendryphantes czekanowskii* Prószyński and *Heliophanus baicalensis* Kulczyński (Araneae, Salticidae) from Siberia. Acta Arachnologica 37: 51–55. doi: 10.2476/asjaa.37.51
202. Marusik YM, Eskov KY (2009) Spiders (Arachnida: Aranei) of the tundra zone of Russia. In: Babenko AB, Matveeva NV, Makarova OL, Golovatch SI (Eds). Vidy i soobshchestva v ekstremalnykh usloviyakh. Sbornik posvyshchennyi 75-letiyu akademika Yuriya Ivanovitcha Chernova. In Pensoft Publishers, KMK Scientist Press, Sofia-Moscow, 92–123. [in Russian]
203. Marusik YM, Eskov KY (2009) Spiders (Arachnida: Aranei) of the tundra zone of Russia. In: Golovatch SI, Makarova OL, Babenko AB, Penev LD (Eds) Species

and Communities in Extreme Environments. Festschrift towards the 75th Anniversary and a Laudatio in Honour of Academician Yuri Ivanovich Chernov. Pensoft Publishers & KMK Scientist Press, Sofia-Moskow, 131–164.

204. Marusik YM, Eskov KY, Kim JP (1992) A check-list of spiders (Aranei) of North-East Asia. Korean Arachnology 8(1/2): 129-158.

205. Marusik YM, Eskov KY, Koponen S, Vinokurov NN (1993). A check-list of the spiders (Aranei) of Yakutia, Siberia. Arthropoda Selecta 2(2): 63–79.

206. Marusik YM, Eskov KY, Logunov DV, Basarukin AM (1993). A check-list of spiders (Arachnida Aranei) from Sakhalin and Kurile Islands. Arthropoda Selecta 1(4): 73–85.

207. Marusik YM, Esyunin SL (1998) A new species of the spider genus *Pelecopsis* Simon, 1864 (Aranei Linyphiidae) from south Siberia. Arthropoda Selecta 6(3–4): 105–108.

208. Marusik YM, Esyunin SL (2010) On the northernmost *Ajmonia* Caporiacco, 1934 (Aranei: Dictynidae: Dictyninae). Journal of Natural History 44(5–6): 361–367. doi:10.1080/00222930903383578

209. Marusik YM, Esyunin SL, Tuneva TK (2015) A survey of Palaearctic Dictynidae (Araneae). 1. Taxonomic notes on *Dictynomorpha* Spassky, 1939, *Brigittea* Lehtinen, 1967 and *Lathys* Simon, 1884. Zootaxa 3925(1): 129–144. doi:10.11646/zootaxa.3925.1.9

210. Marusik YM, Fedoriak MM, Koponen S, Prokopenko EV, Voloshyn VL (2017) Taxonomic notes on two species of Nesticidae (Arachnida: Araneae) in the Ukraine, with the first description of the male of *Carpathonesticus eriashvilii*. Arachnology 17 (6): 302–308. doi: 10.13156/arac.2017.17.6.302

211. Marusik YM, Fet V (2009) A survey of east Palearctic *Hersiliola* Thorell, 1870 (Araneae, Hersiliidae), with a description of three new genera. ZooKeys 16: 75–114. doi:10.3897/zookeys.16.229

212. Marusik YM, Fet V (2021) Kirill Glebovich Mikhailov: On the occasion of his 60th Birthday (Biography). Zootaxa 5006(1): 6–12. doi: 10.11646/zootaxa.5006.1.4

213. Marusik YM, Fomichev AA (2010) A new species of *Parasyrisca* Schenkel, 1963 (Araneae, Gnaphosidae) from the Altai. Zootaxa 2626: 65–68. doi:10.11646/zootaxa.2626.1.4

214. Marusik YM, Fomichev AA (2016) A new species of *Cheiracanthium* (Araneae: Cheiracanthiidae) from Mongolia. Indian Journal of Arachnology 5: 79–83.

215. Marusik YM, Fomichev AA (2016) A survey of East Palaearctic Gnaphosidae (Araneae). 5. On *Synaphosus* from Central Asia. Zootaxa 4178(3): 428–442. doi:10.11646/zootaxa.4178.3.7

216. Marusik YM, Fomichev AA (2016) A survey of East Palaearcrtic Gnaphosidae (Araneae). 7. Review of the *Parasyrisca vinosa*-group. Biological Bulletin of Bogdan Chmelnitskiy Melitopol State Pedagogical University 6(2): 110–118. doi: 10.15421/201643

217. Marusik YM, Fomichev AA (2020) A new genus of Liocranidae (Arachnida: Araneae) from Tajikistan. Acta Biologica Sibirica 6: 583–594. doi:10.3897/abs.6.e59687

218. Marusik YM, Fomichev AA (2020) A new species of *Trachelas* L. Koch, 1872 (Araneae, Trachelidae) from Tajikistan. ZooKeys 993: 27–34. doi:10.3897/zookeys.993.59932

219. Marusik YM, Fomichev AA, Koponen S (2016) Redescription and new data on the distribution of a poorly known pirate spider *Ermetus inopinabilis* (Aranei: Mimetidae). Biological Bulletin of Bogdan Chmelnitskiy Melitopol State Pedagogical University 6(3): 359–364. doi:10.15421/2016105

220. Marusik YM, Fomichev AA, Omelko MM (2014). A survey of East Palaearctic Gnaphosidae (Araneae). 1. On the *Berlandina* Dalmas, 1922 (Gnaphosinae) from Mongolia and adjacent regions. Zootaxa 3827(2): 187–213. doi:10.11646/zootaxa.3827.2.4

221. Marusik YM, Fomichev AA, Omelko MM (2014). A survey of East Palaearctic Gnaphosidae (Araneae). 2. Two new *Gnaphosa* Latreille, 1804 species from Western Mongolia. ZooKeys 426: 1–9. doi:10.3897/zookeys.426.7898

222. Marusik YM, Fomichev AA, Omelko MM (2019) New data on *Parasyrisca*

(Araneae: Gnaphosidae) from Mongolia. Zootaxa 4688(2), 199–212. doi: 10.11646/zootaxa.4688.2.2

223. Marusik YM, Fomichev AA, Tuneva TK (2016) Redescription of *Asagena semideserta* (Ponomarev, 2005) comb. n. (Araneae: Theridiidae). Acta Zoologica Bulgarica 68(4): 477–482.

224. Marusik YM, Fomichev AA, Vahtera V (2018) A new species of *Chalcoscirtus* (Araneae: Salticidae) from Altai, South Siberia, Russia. Entomologica Fennica 29(1): 39–48. doi: 10.33338/ef.70079

225. Marusik YM, Fritzén NR (2009) A new species of *Parasyrisca* (Araneae: Gnaphosidae) from Xinxiang, China. Zootaxa 1982: 63–65. doi:10.11646/zootaxa.1982.1.3

226. Marusik YM, Fritzén NR (2009) A new wolf spider species in the *Pardosa monticola*-group (Araneae: Lycosidae) from Xinjiang, China. Journal of Natural History 43(7–8): 411–422. doi:10.1080/00222930802598409

227. Marusik YM, Fritzén NR (2011) On a new *Dictyna* species (Araneae, Dictynidae) from the northern Palaearctic confused with the east Siberian *D. schmidti* Kulczyński, 1926. ZooKeys 138: 93–108. doi:10.3897/zookeys.138.1849

228. Marusik YM, Fritzén NR, Song DX (2007) On spiders (Aranei) collected in central Xinjiang, China. Arthropoda Selecta 15(3, 2006): 259–276. [publ. July 16, 2007]

229. Marusik YM, Gerlach J (2011) First description of the male of *Sason sechellanum* Simon, 1898 (Araneae: Barychelidae). Zootaxa 2833: 65–68. doi:10.11646/zootaxa.2833.1.7

230. Marusik YM, Gnelitsa VA (2009) A remarkable new species of *Ceratinella* Emerton, 1882 (Araneae, Linyphiidae, Erigoninae) from the Russian Far East. Acta Zootaxonomica Sinica 34(4): 722–724.

231. Marusik YM, Gnelitsa VA (2009) Description of a new genus of spiders from the eastern Mediterranean and the most armored erigonid species from the western Caucasus (Aranei: Linyphiidae: Erigoninae). Arthropoda Selecta 18(1–2): 57–68.

232. Marusik YM, Gnelitsa VA, Koponen S (2006) A survey of Holarctic Linyphiidae (Araneae). 1. A review of the Palaearctic genus *Notioscopus* Simon, 1884. Bulletin

of the British Arachnological Society 13(8): 321–328.

233. Marusik YM, Gnelitsa VA, Koponen S (2007) A survey of Holarctic Linyphiidae (Aranei). 4. A review of the erigonine genus *Lophomma* Menge, 1868. Arthropoda Selecta 15(2, 2006): 153–171. [publ. Mar. 23, 2007]

234. Marusik YM, Gnelitsa VA, Koponen S (2008) A survey of Holarctic Linyphiidae (Araneae). 3. A review of the genus *Praestigia* Millidge, 1954. Bulletin of the British Arachnological Society 14(5): 213–231. doi:10.13156/arac.2008.14.5.213

235. Marusik YM, Gnelitsa VA, Kovblyuk MM (2005) A new species of *Synaphris* (Araneae, Synaphridae) from Ukraine. Bulletin of the British Arachnological Society 13(4): 125–130.

236. Marusik YM, Guseinov EF (2003) Spiders (Arachnida: Aranei) of Azerbaijan. 1. New family and genus records. Arthropoda Selecta 12(1): 29–46.

237. Marusik YM, Guseinov EF, Aliev HA (2004) Spiders (Arachnida: Aranei) of Azerbaijan 4. Fauna of Nakhchivan. Arthropoda Selecta 13(3, 2004): 135–149.

238. Marusik YM, Guseinov EF, Koponen S (2003) A survey of east Palaearctic Lycosidae (Araneae). I. On three closely related species of the *Pardosa falcata*-group. Acta Arachnologica 52(1): 43–50. doi: 10.2476/asjaa.52.43

239. Marusik YM, Guseinov EF, Koponen S (2003) Spiders (Arachnida: Aranei) of Azerbaijan. 2. Critical survey of wolf spiders (Lycosidae) found in the country with description of three new species and brief review of Palaearctic *Evippa* Simon, 1885. Arthropoda Selecta 12(1): 47–65.

240. Marusik YM, Guseinov EF, Koponen S, Yoshida H (2005) A new case of Caucasus-Far East disjunctive range in spiders (Araneae). Acta Arachnologica 53(2, 2004): 125–129.

241. Marusik YM, Hippa H, Koponen S (1996) Spiders (Araneae) from the Altai area, southern Siberia. Acta Zoologica Fennica 201: 11–45.

242. Marusik YM, Khrulyova OA (2011) First data on spiders and harvestmen (Arachnida: Aranei, Opiliones) from Karaginski Island, Eastern Koryakia, Kamchatka Peninsula. Arthropoda Selecta 20(4): 323–329. doi: 10.15298/arthsel.20.4.07

243. Marusik YM, Koponen S (1992) A review of *Meta* (Araneae, Tetragnathidae), with description of two new species. Journal of Arachnology 20: 137–143.
244. Marusik YM, Koponen S (1998) New and little known spiders of the subfamily Dictyninae (Araneae: Dictynidae) from south Siberia. Entomological Problems 29(2): 79–86.
245. Marusik YM, Koponen S (2000) Circumpolar diversity of spiders: implications for research, conservation and management. Annales Zoologici Fennici 37: 265–269.
246. Marusik YM, Koponen S (2000) New data on spiders (Aranei) from the Maritime Province, Russian Far East. Arthropoda Selecta 9(1): 55–68.
247. Marusik YM, Koponen S (2001) Description of a new species and new records of some species of the genus *Gnaphosa* (Araneae: Gnaphosidae) from east Palaearctic. Acta Arachnologica 50(2): 135–144. doi: 10.2476/asjaa.50.135
248. Marusik YM, Koponen S (2001) Revision of the Holarctic spider genus *Procerocymbium* Eskov 1989 (Araneae: Linyphiidae). Acta Arachnologica 50(2): 145–156. doi: 10.2476/asjaa.50.145
249. Marusik YM, Koponen S (2001) Spiders of the family Zodariidae from Mongolia (Arachnida: Araneae). Reichenbachia 34(4): 39–48.
250. Marusik YM, Koponen S (2002) Diversity of spiders in boreal and arctic zones. Journal of Arachnology 30(2): 205–210. doi: 10.1636/0161-8202(2002)030[0205:DOSIBA]2.0.CO;2
251. Marusik YM, Koponen S (2002) *Vermontia*, a linyphiid genus new to the Palaearctic, and new records of spiders from northeast Asia (Araneae). Bulletin of the British Arachnological Society 12(4): 159–165.
252. Marusik YM, Koponen S (2005) A survey of spiders (Araneae) with Holarctic distribution. Journal of Arachnology 33(2): 300–305. doi: 10.1636/04-115.1
253. Marusik YM, Koponen S (2008) A survey of the Siberian erigonine genus *Perlongipalpus* (Aranei: Linyphiidae: Erigoninae) with description of two new species. Arthropoda Selecta 17(1–2): 55–63.
254. Marusik YM, Koponen S (2009) A new species of *Uusitaloia* Marusik, Koponen & Danilov (Araneae: Linyphiidae: Erigoninae), a genus through to be monotypic,

from the Wrangel Island NE Russia. Entomologica Fennica 20(1): 18–21. doi: 10.33338/ef.84455

255. Marusik YM, Koponen S (2010) A review of the Holarctic genus *Tmeticus* Menge, 1868 (Araneae, Linyphiidae), with a description of a new genus. ZooKeys 59: 15–37. doi:10.3897/zookeys.59.508
256. Marusik YM, Koponen S (2015) New biogeographical records of spiders and harvestmen (Arachnida: Araneae & Opiliones) from West Siberia, including an annotated list of species. Entomologica Fennica 26(4): 165–170. doi: 10.33338/ef.53371
257. Marusik YM, Koponen S (2017) On two sibling species of *Dictyna* (Araneae: Dictynidae) from Ukraine and Caucasus. Entomologica Fennica 28(1): 41–48.
258. Marusik YM, Koponen S, Danilov SN (2001). Taxonomic and faunistic notes on linyphiids of Transbaikalia and south Siberia (Araneae, Linyphiidae). Bulletin of the British Arachnological Society 12(2): 83–92.
259. Marusik YM, Koponen S, Fritzén NR (2009) On two sibling *Lathys* species (Araneae, Dictynidae) from northern Europe. ZooKeys 16: 181–195. doi:10.3897/zookeys.16.228
260. Marusik YM, Koponen S, Makarova OL (2016) A survey of spiders (Araneae) collected on the arctic island of Dolgiy (69°12′N), Barents Sea. Arachnology 17(1): 10–24. doi:10.13156/arac.2006.17.1.10
261. Marusik YM, Koponen S, Potapova NK (2004) Spiders (Aranei) from Oymyakon, the cold pole of the northern hemisphere (Yakutia, Siberia). Arthropoda Selecta 13(1–2): 69–75.
262. Marusik YM, Koponen S, Vinokurov NN, Nogovitsyna SN (2002) Spiders (Aranei) from northernmost forest-tundra of northeastern Yakutia (70°35'N, 134°34'E) with description of three new species. Arthropoda Selecta 10(4): 351–370
263. Marusik YM, Koponen S, Zonstein SL (2017) A revalidation and redescription of *Sahastata infuscata*, with notes on *S. nigra* (Araneae: Filistatidae). Arachnology 17(6): 309–311. doi:10.13156/arac.2017.17.6.309
264. Marusik YM, Kovblyuk MM (2004) New and interesting cribellate spiders from

Abkhazia (Aranei: Amaurobiidae, Zoropsidae). Arthropoda Selecta 13(1): 55–61.
265. Marusik YM, Kovblyuk MM (2009) Redescription of *Minosiella intermedia* Denis, 1958 (Araneae: Gnaphosidae) with first description of the male. Zootaxa 2291: 65–68. doi:10.11646/zootaxa.2291.1.5
266. Marusik YM, Kovblyuk MM (2010) The spider genus *Trachelas* L. Koch, 1872 (Aranei: Corinnidae) in Russia. Arthropoda Selecta 19(1): 21–27. doi:10.15298/arthsel.19.1.04
267. Marusik YM, Kovblyuk MM (2011) Spiders (Arachnida, Aranei) of Siberia and Russian Far East. KMK Scientific Press, Moscow, 344 pp. [in Russian]
268. Marusik YM, Kovblyuk MM, Koponen S (2011) A survey of the east Palaearctic Lycosidae (Araneae). 9. Genus *Xerolycosa* Dahl, 1908 (Evippinae). ZooKeys 119: 11–27. doi:10.3897/zookeys.119.1706
269. Marusik YM, Kovblyuk MM, Nadolny AA (2009) A survey of *Lathys* Simon, 1884, from Crimea with resurrection of *Scotolathys* Simon, 1884 (Aranei: Dictynidae). Arthropoda Selecta 18(1–2): 21–33.
270. Marusik YM, Kovblyuk MM, Ponomarev A. V. (2010). A new subfamily of amaurobiid spiders (Aranei: Amaurobiidae) from west Caucasus. Arthropoda Selecta 19(4): 227–236. doi:10.15298/arthsel.19.4.03
271. Marusik YM, Kunt KB (2009) Spiders (Aranei) new to the fauna of Turkey. 3. Genus and species records of Hahniidae. Arthropoda Selecta 18(1–2): 77–80.
272. Marusik YM, Kunt KB (2009). Spiders (Araneae) new to the fauna of Turkey. 7. New species and genera records of Linyphiidae. Serket 11(3–4): 82–86.
273. Marusik YM, Kunt KB (2010) Spiders (Araneae) new to the fauna of Turkey. 1. Genera of Theridiidae. Turkish Journal of Arachnology 3(1): 1–8.
274. Marusik YM, Kunt KB (2010) Spiders (Araneae) new to the fauna of Turkey. 4. New species records of Clubionidae. Turkish Journal of Arachnology 3(1): 13–15.
275. Marusik YM, Kunt KB (2011) Spiders (Aranei) new to the fauna of Turkey. 9. Two new family records (Mysmenidae and Synaphridae) and one species record of Mimetidae. Caucasian Entomological Bulletin 7(1): 3–5.
276. Marusik YM, Kunt KB, Danışman T (2009) Spiders (Aranei) new to the fauna of

Turkey. 2. New species records of Theridiidae. Arthropoda Selecta 18(1–2): 69–75.

277. Marusik YM, Kunt KB, Yağmur EA (2010) A new species of *Hersiliola* Thorell, 1870 (Araneae, Hersiliidae) from Turkey. ZooKeys 37: 27–34. doi:10.3897/zookeys.37.398

278. Marusik YM, Kuzminykh GV (2010) On two spider genera new to Russia (Aranei: Corinnidae, Sparassidae). Arthropoda Selecta 19(2): 97–100. doi:10.15298/arthsel.19.2.07

279. Marusik YM, Larsen N (2018) A synopsis of African *Metellina* (Aranei, Tetragnathidae, Metainae) with description of a new species from South Africa. Vestnik Zoologii 52(3): 205–216. doi:10.2478/vzoo–2018–0021

280. Marusik YM, Leech R (1993) The spider genus *Hypselistes*, including two new species, from Siberia and the Russian Far East (Araneida: Erigonidae). The Canadian Entomologist 125(6): 1115–1126. doi: 10.4039/Ent1251115-6

281. Marusik YM, Lehtinen PT (2003) Synaphridae Wunderlich, 1986 (Aranei: Araneoidea), a new family status, with a description of a new species from Turkmenistan. Arthropoda Selecta 11(2): 143–152.

282. Marusik YM, Lehtinen PT, Kovblyuk MM (2005) *Cozyptila*, a new genus of crab spiders (Aranei: Thomisidae: Thomisinae: Coriarachnini) from the western Palaearctic. Arthropoda Selecta 13(3, 2004): 151–163 & Erratum 14(1): 18. [publ. Mar. 15, 2005; Erratum Jul. 20, 2005]

283. Marusik YM, Li SQ (2011) About types of arachnids (Araneae, Opiliones, Pseudoscorpiones) described by E. Schenkel from China in 1953. Zootaxa 3002: 59–61. doi: 10.11646/zootaxa.3002.1.6

284. Marusik YM, Logunov DV (1990) The crab spiders of Middle Asia, USSR (Aranei, Thomisidae). 1. Descriptions and notes on distribution of some species. Korean Arachnology 6(1): 31–62.

285. Marusik YM, Logunov DV (1991) Spiders of the superfamily Amaurobioidea (Aranei) from Sakhalin and Kurily Islands. Zoologicheskiĭ Zhurnal 70(9): 87–94. [in Russian]

286. Marusik YM, Logunov DV (1995) Gnaphosid spiders from Tuva and adjacent

territories, Russia. Beiträge zur Araneologie 4(1994): 177–210. [publ. in Dec. 1995]

287. Marusik YM, Logunov DV 1991 (1995). Poorly known spider species of the families Salticidae and Thomisidae (Aranei) from the Far East of USSR. In: Entomological Studies in the North-East of the USSR. USSR Academy of Sciences, Institute of Biological Problems of the North, Vladivostok 2: 131–140. (dated "1991," first distributed March, 1996) [In Russian]
288. Marusik YM, Logunov DV (1995). The crab spiders of Middle Asia (Aranei, Thomisidae), 2. Beiträge zur Araneologie 4(1994): 133–175. [publ. in Dec. 1995]
289. Marusik YM, Logunov DV (1998) Taxonomic notes on the *Evarcha falcata* species complex (Aranei Salticidae). Arthropoda Selecta 6(3–4): 95–104.
290. Marusik YM, Logunov DV (1999) On the spiders (Aranei) collected in central Mongolia during a joint American–Mongolian–Russian expedition in 1997. Arthropoda Selecta 7(3): 233–254.
291. Marusik YM, Logunov DV (2002). New and poorly known species of crab spiders (Aranei: Thomisidae) from south Siberia and Mongolia. Arthropoda Selecta 10(4): 315–322.
292. Marusik YM, Logunov DV (2002) New faunistic records for the spiders of Buryatia (Aranei), with a description of a new species from the genus *Enoplognatha* (Theridiidae). Arthropoda Selecta 10(3): 265–272.
293. Marusik YM, Logunov DV (2006) On the spiders collected in Mongolia by Dr. Z. Kaszab during expeditions in 1966–1968 (Arachnida, Aranei (excluding Lycosidae)). Arthropoda Selecta 15(1): 39–57.
294. Marusik YM, Logunov DV (2009) New faunistic records of spiders collected from the mountain Altai (Arachnida: Aranei). Arthropoda Selecta 18(3–4): 145–152.
295. Marusik YM, Logunov DV (2011) New faunistic records of spiders from east Kazakhstan (Arachnida: Aranei). Arthropoda Selecta 20(1): 57–63. doi:10.15298/arthsel.20.1.04
296. Marusik YM, Logunov DV (2017) New faunistic and taxonomic data on spiders (Arachnidae: Aranei) from the Russian Far East. Acta Arachnologica 66(2): 87–

96. doi:10.2476/asjaa.66.87

297. Marusik YM, Logunov DV, Koponen S (2000). Spiders of Tuva, south Siberia. Institute for Biological Problems of the North, Magadan, 253 pp.

298. Marusik YM, Mikhailov KG (2010). First description of the male of *Castianeira arnoldii* Charitonov, 1946 (Aranei: Corinnidae) from central Asia, and a survey of Palaearctic *Castianeira*. Arthropoda Selecta 19(2): 91–95. doi:10.15298/arthsel.19.2.06

299. Marusik YM, Mikhailov KG (2021) Revalidation of *Xysticus tuberosus* Thorell, 1875 (Aranei: Thomisidae) with notes on the related species. Arthropoda Selecta 30(1): 119–124. doi:10.15298/arthsel.30.1.11

300. Marusik YM, Mikhailov KG, Guseinov EF (2005) History and perspectives of study of the spider fauna of Transcaucasia. Biological Diversity of Caucasus. Proceedings of the Third International Conference, Nalchik (Russia), October 2004. 185–190. [In Russian]

301. Marusik YM, Mikhailov KG, Guseinov EF (2006) Advances in study of biodiversity of Caucasian spiders (Araneae). European Arachnology 2005 (Deltshev C & Stoev P eds). Acta Zoologica Bulgarica Suppl. 1: 259–268.

302. Marusik YM, Mikhailov KG, Kuzmin EA (2015) On the synonymy of *Tetragnatha qiuae* (Aranei: Tetragnathidae). Arthropoda Selecta 24(4): 443–444. doi:10.15298/arthsel.24.4.07

303. Marusik YM, Mikhailov KG, Omelko MM (2015) Taxonomic notes on spiders (Arachnida: Aranei) of the Russian Far East. Arthropoda Selecta 24(1): 117–124. doi:10.15298/arthsel.24.1.09

304. Marusik YM, Nadimi A, Omelko MM, Koponen S (2014). First data about cave spiders (Arachnida: Araneae) from Iran. Zoology in the Middle East 60(3): 255–266. doi:10.1080/09397140.2014.943465

305. Marusik YM, Nadolny AA (2018) Redescription of enigmatic spider genus *Stoliczka* O. Pickard-Cambridge, 1885 (Araneae: Lycosoidea) based on the type species. Zootaxa 4497(2): 295–300. doi:10.11646/zootaxa.4497.2.9

306. Marusik YM, Nadolny AA (2020) On the identity of *Trochosa hispanica* (Araneae,

Lycosidae), with notes on the synonymy of West Palaearctic "*Trochosa*" species. Zootaxa 4859(1): 56–80. doi:10.11646/zootaxa.4859.1.2

307. Marusik YM, Nadolny AA (2021) Redescription of *Hippasa deserticola*, the northernmost species of *Hippasa* (Aranei: Lycosidae), with taxonomic notes on other species of the genus. Zoosystematica Rossica 30(2): 222–235. doi:10.31610/zsr/2021.30.2.222
308. Marusik YM, Nadolny AA, Koponen S (2018) A survey of the *Alopecosa cursor* species group (Aranei: Lycosidae) from Asia. Arthropoda Selecta 27(4): 348–362. doi:10.15298/arthsel.27.4.12
309. Marusik YM, Nadolny AA, Koponen S (2020) Redescription of *Trochosa urbana* (Araneae: Lycosidae) with notes on its distribution. Arachnology 18(5), 482–489. doi.org/10.13156/arac.2020.18.5.482
310. Marusik YM, Nadolny AA, Omelko MM (2013) A survey of east Palearctic Lycosidae (Araneae). 10. Three new *Pardosa* species from the mountains of central Asia. Zootaxa 3722(2): 204–218. doi:10.11646/zootaxa.3722.2.5
311. Marusik YM, Nekhaeva AA (2020) New data on spiders (Arachnida, Aranei) of Kamchatka, Russia. Acta Biologica Sibirica 6: 649–668. doi:10.3897/abs.6.e60005
312. Marusik YM, Nekhaeva AA (2020). Redescription of two poorly known Arctic *Hilaira* species (Aranei: Linyphiidae) with notes on species grouping. Arthropoda Selecta 29(1): 133–140. doi:10.15298/arthsel.29.1.12
313. Marusik YM, Nekhaeva AA, Koponen S (2019) On four sibling *Erigone* species occurring in the Arctic (Aranei: Linyphiidae). Arthropoda Selecta 28(1): 135–146. doi:10.15298/arthsel.28.1.12
314. Marusik YM, Omelko MM (2008) First description of the female of *Ozyptila utotchkini* (Araneae: Thomisidae). Zootaxa 1823: 65–68. doi:10.11646/zootaxa.1823.1.5
315. Marusik YM, Omelko MM (2011) A survey of East Palaearctic Lycosidae (Araneae). 7. A new species of *Acantholycosa* Dahl, 1908 from the Russian Far East. ZooKeys 79: 1–10. doi:10.3897/zookeys.79.945

316. Marusik YM, Omelko MM (2012) Redescription of *Cryptothele alluaudi* Simon, 1893 (Aranei: Cryptothelidae). Arthropoda Selecta 21(2): 183–186. doi:10.15298/arthsel.21.2.06
317. Marusik YM, Omelko MM (2013) First description of the male of *Cryptothele verrucosa* L. Koch, 1872 (Araneae), the type species of the genus. ZooKeys 351: 31–36. doi:10.3897/zookeys.351.6255
318. Marusik YM, Omelko MM (2014). A survey of East Palaearctic Gnaphosidae (Araneae). 3. On new and poorly known *Gnaphosa* Latreille, 1804. Zootaxa 3894(1): 10–32. doi:10.11646/zootaxa.3894.1.4
319. Marusik YM, Omelko MM (2014). Reconsideration of *Xysticus* species described by Ehrenfried Schenkel from Mongolia and China in 1963 (Araneae: Thomisidae). Zootaxa 3861(3): 275–289. doi:10.11646/zootaxa.3861.3.5
320. Marusik YM, Omelko MM (2015) Redescription of "*Amaurobius*" *rufipes* Taczanowski, 1874 (Araneae: Corinnidae) with notes of *Amaurobius* species described from French Guyana. Zootaxa 3974(1): 148–150. doi:10.11646/zootaxa.3974.1.14
321. Marusik YM, Omelko MM (2016). Redescription of *Draposa subhadrae* (Patel & Reddy, 1993) (Araneae: Lycosidae, Pardosinae). Zootaxa 4107(1): 89–92. doi:10.11646/zootaxa.4107.1.6
322. Marusik YM, Omelko MM (2016). Redescription of two Ctenidae (Araneae) from Taiwan. Zootaxa 4173(2): 155–162. doi:10.11646/zootaxa.4173.2.6
323. Marusik YM, Omelko MM (2017) A new genus of tetragnathid spiders from Papua New Guinea (Aranei, Tetragnathidae). Vestnik Zoologii 51(3): 203–214. doi:10.1515/vzoo–2017–0027
324. Marusik YM, Omelko MM (2017) A new species of *Acantholycosa baltoroi*-group (Araneae: Lycosidae, Pardosinae) from the Russian Far East. Zootaxa 4232(4): 597–600. doi:10.11646/zootaxa.4232.4.12
325. Marusik YM, Omelko MM (2017) A new species of *Tekellina* (Araneae, Araneoidea) from the Russian Far East. Entomologica Fennica 28(3): 164–168. doi: 10.33338/ef.84684

326. Marusik YM, Omelko MM (2017) First illustrated description of the male of *Diphya macrophthalma*, the type species of the genus (Araneae, Tetragnathidae). Iheringia, Série Zoologia 107(e2017036): 1–7. doi:10.1590/1678–4766e2017036
327. Marusik YM, Omelko MM (2017) Redescription of *Micaria beaufortia* (Araneae, Gnaphosidae), with notes on Afrotropical *Micaria*. Zootaxa 4294(4): 462–470. doi:10.11646/zootaxa.4294.4.5
328. Marusik YM, Omelko MM (2018) A survey of the *Porrhoclubiona* Lohmander, 1944 from Central Asia (Araneae, Clubionidae). ZooKeys 802: 19–38. doi:10.3897/zookeys.802.30236
329. Marusik YM, Omelko MM (2018) First record of *Eupoa pulchella* (Araneae: Salticidae) in Laos with description of previously unknown female. Far Eastern Entomologist 354: 19–22.
330. Marusik YM, Omelko MM (2018) New data on *Synaphosus* (Araneae: Gnaphosidae) from Southeast Asia. Zootaxa 4374(2): 235–248. doi:10.11646/zootaxa.4374.2.4
331. Marusik YM, Omelko MM (2019) Redescription of gnaphosids (Aranei: Gnaphosidae) described by O. Pickard-Cambridge from the material of the Second Yarkand Mission. Arthropoda Selecta 28(2): 277–290. doi:10.15298/arthsel.28.2.11
332. Marusik YM, Omelko MM (2021) A new genus record of an enigmatic linyphiid spider (Aranei: Linyphiidae) from the Russian Far East. Arthropoda Selecta 30(4): 565–571. doi: 10.15298/arthsel.30.4.13
333. Marusik YM, Omelko MM (2021) A new species and new records of *Cybaeus* L. Koch, 1868 (Aranei: Cybaeidae) in the Maritime Province of Russia. Arthropoda Selecta 30(2): 216–220. doi:10.15298/arthsel.30.2.08
334. Marusik YM, Omelko MM, Benjamin SP (2013) The first description of adult female of *Borboropactus asper* (O. P.-Cambridge, 1884) from Sri Lanka (Araneae: Thomisidae). Zootaxa 3737(2): 197–200. doi:10.11646/zootaxa.3737.2.8
335. Marusik YM, Omelko MM, Koponen S (2010) A survey of the east Palearctic

Lycosidae (Aranei). 5. Taxonomic notes on the easternmost Palearctic *Pirata* species and on the genus *Piratosa* Roewer, 1960. Arthropoda Selecta 19(1): 29–36. doi:10.15298/arthsel.19.1.05

336. Marusik YM, Omelko MM, Koponen S (2011) On the distribution of *Gnaphosa orites* (Araneae: Gnaphosidae) with a note on its southernmost record, from the Russian Far East. Acta Arachnologica 60(1): 51–53. doi:10.2476/asjaa.60.51
337. Marusik YM, Omelko MM, Koponen S (2013) Redescription of *Pterotricha loeffleri* (Roewer, 1955) (Aranei: Gnaphosidae: Gnaphosinae). Arthropoda Selecta 22(4): 349–352. doi:10.15298/arthsel.22.4.05
338. Marusik YM, Omelko MM, Koponen S (2015). A redescription of the ant mimicking spider *Myrmecium gounellei* (Araneae: Corinnidae, Castianeirinae), with notes on the genus. Zootaxa 3985(2): 296–300. doi:10.11646/zootaxa.3985.2.9
339. Marusik YM, Omelko MM, Koponen S (2015) A survey of East Palaearctic Lycosidae (Araneae). 11. Two new genera from the *Acantholycosa* complex. Zootaxa 3985(2): 252–264. doi:10.11646/zootaxa.3985.2.4
340. Marusik YM, Omelko MM, Koponen S (2015) Redescription of *"Nesticus" citrinus* (Taczanowski, 1874) (Araneae, Araneoidea) from French Guyana. ZooKeys 537: 97–101. doi:10.3897/zookeys.537.6818
341. Marusik YM, Omelko MM, Koponen S (2015). Redescription of *Oecobius tadzhikus* Andreeva et Tyshchenko, 1969 (Aranei: Oecobiidae). Arthropoda Selecta 24(2): 197–200. doi:10.15298/arthsel.24.2.05
342. Marusik YM, Omelko MM, Koponen S (2016) Two new *Bifurcia* species (Aranei: Linyphiidae: Micronetinae) from Far East Russia. Arthropoda Selecta 25(1): 77–83. doi:10.15298/arthsel.25.1.08
343. Marusik YM, Omelko MM, Koponen S (2016) Rare and new for the fauna of the Russian Far East spiders (Aranei). Far Eastern Entomologist 317: 1–15.
344. Marusik YM, Omelko MM, Koponen S **(2017)** First record of *Diphya wulingensis* Yu, Zhang et Omelko, 2014 (Aranei: Tetragnathidae) in

Russia. Arthropoda Selecta 26(2): 139–144. doi:10.15298/arthsel.26.2.07

345. Marusik, Y M, Omelko MM, Koponen S (2018) Redescription of the Himalayan *Trachelas costatus* (Araneae: Trachelidae). Zootaxa 4433(2): 390–392. doi: 10.11646/zootaxa.4433.2.11
346. Marusik YM, Omelko MM, Koponen S (2020) On two enigmatic spiders (Araneae: Cybaeidae & Phrurolithidae) from the Russian Far East. Zootaxa 4899(1): 247–258. doi:10.11646/zootaxa.4899.1.13
347. Marusik YM, Omelko MM, Ponomarev AV (2017). A survey of the Holarctic genus *Arctella* Holm, 1945 (Araneae: Dictynidae, Tricholathysinae), with the description of *Tricholathys ovtchinnikovi* sp. n. Oriental Insects 51(3): 246–261. doi:10.1080/00305316.2017.1279086
348. Marusik YM, Omelko MM, Ryabukhin AS (2013) New data on spiders (Aranei) from eastern Koryakia, Kamchatka Peninsula. Arthropoda Selecta 22(4): 363–377. doi:10.15298/arthsel.22.4.09
349. Marusik YM, Omelko MM, Simmons ZM (2020) Redescription of two west Himalayan *Cheiracanthium* (Aranei: Cheiracanthiidae). Arthropoda Selecta 29(3): 339–347. doi:10.15298/arthsel.29.3.07
350. Marusik YM, Omelko MM, Simonov PS (2020) A redescription of *Aculepeira matsudae* (Aranei: Araneidae), a species recently found in Far East Russia. Arthropoda Selecta 29(1): 121–126. doi:10.15298/arthsel.29.1.10
351. Marusik YM, Omelko MM, Simonov PS, Koponen S (2015) New data about orb-weaving spiders (Aranei: Araneidae and Tetragnathidae) from the Russian Far East. Arthropoda Selecta 24(2): 207–214. doi:10.15298/arthsel.24.2.07
352. Marusik YM, Omelko MM, Simonov PS, Mi XQ (2020) A survey of *Alenatea* Song et Zhu, 1999 (Aranei: Araneidae). Arthropoda Selecta 29(4): 487–494. doi:10.15298/arthsel.29.4.12
353. Marusik YM, Otto S, Japoshvili G (2020) Taxonomic notes on *Amaurobius* (Araneae: Amaurobiidae), including the description of a new species. Zootaxa 4718(1): 47–56. doi:10.11646/zootaxa.4718.1.3
354. Marusik YM, Ovchinnikov SV, Koponen S (2006) Uncommon conformation of the

male palp in common Holarctic spiders belonging to the *Lathys stigmatisata* group (Araneae, Dictynidae). Bulletin of the British Arachnological Society 13(9): 353–360.

355. Marusik YM, Özkütük RS, Kunt KB (2012) Spiders (Araneae) new to the fauna of Turkey. 10. Two new species records of Tetragnathidae. Anadolu University Journal of Science and Technology, C, Life Sciences and Biotechnology 2(2): 69–73.
356. Marusik YM, Özkütük RS, Kunt KB (2013) On the identity and distribution of the poorly known spider *Orthobula charitonovi* (Mikhailov, 1986) (Aranei: Corinnidae). Arthropoda Selecta 22(2): 157–162. doi:10.15298/arthsel.22.2.05
357. Marusik YM, Özkütük RS, Kunt KB, Kaya RS (2011) Spiders (Araneae) new to the fauna of Turkey. 8. New records of Hahniidae and Dicytnidae. Anadolu University Journal of Science and Technology, Life Sciences and Biotechnology 1(2): 161–170.
358. Marusik YM, Penney D (2004) A survey of Baltic amber Theridiidae (Araneae) inclusions, with descriptions of six new species. In: Logunov DV & Penney D (eds.) European Arachnology 2003. Proceedings of the 21st European Colloquium of Arachnology, St.-Petersburg, 4–9 August 2003. Arthropoda Selecta, Special Issue 1, 201–218.
359. Marusik YM, Perkovsky E, Eskov KY (2018) First records of spiders (Arachnida: Aranei) from Sakhalinian Amber with description of a new species of the genus *Orchestina* Simon, 1890. Far Eastern entomologist 367, 1-9. doi: 10.25221/fee.367.1
360. Marusik YM, Ryabukhin AS, Kuzminykh GV (2010) New data on spiders and harvestmen (Arachnida: Aranei & Opiliones) from Western Koryakia, Kamchatka Peninsula. Arthropoda Selecta 19(4): 283–293. doi:10.15298/arthsel.19.4.10
361. Marusik YM, Rybalov LB, Koponen S, Tanasevitch AV (2002). Spiders (Aranei) of middle Siberia, an updated check-list with a special reference to the Mirnoye Field Station. Arthropoda Selecta 10(4): 323–350.
362. Marusik YM, Saaristo MI (1999) Review of East Palearctic species of the genus

Minicia Thorell, 1875 with descriptions of two new genera (Aranei: Linyphiidae: Erigoninae). Arthropoda Selecta 8(2): 125–130.

363. Marusik YM, Seropian A, Koponen S (2019) On the northernmost record of *Pritha* (Aranei: Filistatidae) in the Caucasus and entire Asia with notes on Filistatidae from Caucasus. Arthropoda Selecta 28(3): 403–407. doi:10.15298/arthsel.28.3.04
364. Marusik YM, Šestáková A, Omelko MM (2012) First description of the male with redescription of the female of *Araneus strandiellus* Charitonov, 1951 (Araneae, Araneidae). ZooKeys 205: 91–98. doi:10.3897/zookeys.205.3077
365. Marusik YM, Tanasevitch AV (1998) Notes on the spider genus *Styloctetor* Simon, 1884 and some related genera, with description of two new species from Siberia (Aranei: Linyphiidae). Arthropoda Selecta 7(3): 153–159.
366. Marusik YM, Tanasevitch AV (2003) Two new erigonine spiders (Aranei: Linyphiidae) from mountains of south Siberia. Arthropoda Selecta 11(2): 159–165.
367. Marusik YM, Tanasevitch AV, Kurenshchikov DK, Logunov DV (2007) A check-list of the spiders (Araneae) of the Bolshekhekhtsyrski Nature Reserve, Khabarovsk Province, the Russian Far East. Acta Arachnologica Sinica 16(1): 37–64.
368. Marusik YM, Tarabayev CK (1991) The catalogue of orbweaving spiders in Kazakhstan. 2. Families: Tetragnathidae, Uloboridae. Khabarly Kazakhstan SSR Academy of Sciences 164(2): 26–28. [In Russian]
369. Marusik YM, Tarabaev CK (1995) A new species and two interesting records of the black-widow spiders from Middle Asia and the Caucasus (Aranei, Theridiidae). Beiträge zur Araneologie 4(1994): 211–215. [publ. in Dec. 1995]
370. Marusik YM, Tarabayev CK, Litovchenko AM (1990) The catalogue of orb-weaving spiders in Kazakhstan. Familia: Araneidae. Khabarly Kazakhstan SSR Academy of Sciences 4(160): 14—23. [In Russian]
371. Marusik YM, Tarabaev CK, Litovchenko AM (1991) Katalog paukov-krugopyadov Kazakhstana. Semeistvo Araneidae. Izvestiya Akademii Nauk Kazakhskoi SSR Seriya Biologicheskikh Nauk (Biol.) 1990(4): 14–22.

372. Marusik YM, Tsellarius AY (1986) New species of spiders of the genus *Theridion* (Aranei, Theridiidae) from Karelia. Zoologicheskiĭ Zhurnal 65(11): 1736–1738. [In Russian]
373. Marusik YM, Wunderlich J (2008) A survey of fossil Oonopidae (Arachnida: Aranei). Arthropoda Selecta 17(1–2): 65–79.
374. Marusik YM, Zamani A (2015) Additional new species of Filistatidae (Aranei) from Iran. Arthropoda Selecta 24(4): 429–435. doi:10.15298/arthsel.24.4.05
375. Marusik YM, Zamani A (2015) First description of the male of *Tegenaria zamanii* Marusik & Omelko, 2014 (Araneae: Agelenidae) from northern Iran. Zootaxa 4052(2): 226–228. doi:10.11646/zootaxa.4052.2.6
376. Marusik YM, Zamani A (2015) The spider family Filistatidae (Araneae) in Iran. ZooKeys 516: 123–135. doi:10.3897/zookeys.516.10146
377. Marusik YM, Zamani A (2016) A new species of *Sahastata* from southern Iran (Aranei, Filistatidae). Vestnik Zoologii 50(3): 267–270. doi: 10.1515/vzoo-2016-0031
378. Marusik YM, Zamani A, Mirshamsi O (2014) Three new species of mygalomorph and filistatid spiders from Iran (Araneae, Cyrtaucheniidae, Nemesiidae and Filistatidae). ZooKeys 463: 1–10. doi:10.3897/zookeys.463.8692
379. Marusik YM, Zhang F, Omelko MM (2012) A new genus and species of ctenid spiders (Aranei: Ctenidae) from south China. Arthropoda Selecta 21(1): 61–66. doi:10.15298/arthsel.21.1.05
380. Marusik YM, Zheng G, Li SQ (2008) A review of the genus *Paratus* Simon (Araneae, Dionycha). Zootaxa 1965: 50–60. doi: 10.11646/zootaxa.1965.1.2
381. Marusik YM, Zheng G, Li SQ (2009) First description of the female of *Echinax panache* Deeleman-Reinhold, 2001 (Aranei: Corinnidae: Castianeirinae). Arthropoda Selecta 17(3–4): 165–168.
382. Marusik YM, Zonstein S (2011) A synopsis of east-Mediterranean *Synaphris* Simon, 1894 (Araneae, Synaphridae) with a description of a new species from Israel. ZooKeys 82: 35–44. doi:10.3897/zookeys.82.957

383. Marusik YM, Zonstein SL (2014) A synopsis of Middle East *Filistata* (Aranei: Filistatidae), with description of new species from Azerbaijan. Arthropoda Selecta 23(2): 199–205. doi:10.15298/arthsel.23.2.09
384. Marusik YM, Zonstein SL (2018) Notes on the spider genus *Steriphopus* (Araneae: Palpimanidae), with redescription of the type species. Arachnology 17(9): 491–496. doi:10.13156/arac.2018.17.9.491
385. Marusik YM, Zonstein SL (2019) Redescription of the Central Asian spider *Pholcoides seclusa* comb. nov. (Araneae: Filistatidae). Arachnologische Mitteilungen: Arachnology Letters 57(1), 43–47. doi: 10.30963/aramit5708
386. Marusik YM, Zonstein SL (2021) Description of *Raveniola ambardzumyani* sp. n. from Armenia (Araneae, Nemesiidae). Israel Journal of Entomology 51: 93–101. doi: 10.5281/zenodo.559646
387. Marusik YM, Zonstein SL, Koponen S (2019) Redescription of a poorly known insular spider *Labahitha gibsonhilli* (Araneae: Filistatidae). Arachnology 18(3), 258–259. doi: 10.13156/arac.2019.18.3.258
388. Marusik YM, Zonstein SL, Omelko MM (2013) First description of the male of *Zelotes haifaensis* Levy, 2009 (Aranei: Gnaphosidae). Arthropoda Selecta 22(3): 255–258. doi:10.15298/arthsel.22.3.08
389. Marusik YM, Zonstein SL, Omelko MM (2015) Redescription of *Phrurolithus flavipes* (Araneae: Phrurolithidae), with the first description of the male. Zootaxa 4020(1): 197–200. doi:10.11646/zootaxa.4020.1.10
390. Ma SC, Marusik YM, Tu LH (2016) A review of the Nearctic linyphiid spider *Arcuphantes fragilis* (Araneae, Linyphiidae) and closely related species. Zootaxa 4144(3): 383–396. doi:10.11646/zootaxa.4144.3.6
391. Medvedev GS, Belokobylskiy SA, Belyaeva NA, Golub VB, Gorokhov AV, Zamotailov AS, Ivanov VD, Knyazev AN, Korotyaev BA, Krishtopa AN, Kuzhnetsova VG, Marusik YM, Medvedev SG, Mozolevskaya EG, Moreva LY, Narchuk EP, Pavlyushin VA, Selikhovkin AV, Sergeev MG, Fasulati SR, Chernyshov VB, Shchurov VI (2008) XIIIth Congress of the Russian Entomological Society. Krasnodar, 9-15 September 2007. Entomologicheskoye

Obozreniye 87(2): 468-482.

392. Mikhailov KG, Marusik YM 1991 (1995) Spiders of the north-east USSR. Families Clubionidae, Zoridae, Liocranidae and Gnaphosidae (genus *Micaria*) (Arachnida, Aranei). In: Entomological Studies in the North-East of the USSR. USSR Academy of Sciences, Institute of Biological Problems of the North, Vladivostok 2: 90–113. (dated "1991," first distributed March, 1996)
393. Mikhaljova EV, Marusik YM (2004) New data on taxonomy and fauna of the millipedes (Diplopoda) from the Russian Far East, Siberia and Mongolia. Far Eastern Entomologis 133: 1–12.
394. Mikhaljova EV, Marusik YM (2005) Millipedes (Diplopoda) of the Kurile Islands. Biodiversity and Biogeography of the Kuril Islands and Sakhalin, Bulletin of the Hokkaido University Museum 3: 115–127.
395. Mirshamsi O, Marusik YM, Jamili E (2013) The spider family Hersiliidae Thorell, 1870 (Arachnida: Araneae) in Iran. Zoology in the Middle East 59: 347–352. doi:10.1080/09397140.2013.868140
396. Mirshamsi O, Marusik YM, Zamani A, Moradmand M, Kashefi R (2015) Annotated checklist of the spiders of Iran (Arachnida: Araneae). Iranian Journal of Animal Biosystematics FAUNA IRANICA 1: 1–108.
397. Mirshamsi O, Shafaie S, Aliabadian M, Moradmand M, Marusik YM (2017) An overview of species concept used in spiders taxonomy with an emphasis on mating structures. Taxonomy and Biosystematics 9(31): 6, 87–99.
398. Mirshamsi O, Zamani A, Marusik YM (2016) A survey of Hersiliidae (Arachnida: Araneae) of Iran with description of one new genus and two new species. Journal of Natural History 50(23–24): 1447–1461. doi:10.1080/00222933.2015.1113318
399. Mozaffarian F, Marusik YM (2001) A checklist of Iranian spiders (Aranei). Arthropoda Selecta 10(1): 67–74.
400. Nadolny AA, Omelko MM, Marusik YM, Blagoev G (2016) A new species of spider belonging to the *Pardosa lugubris*-group (Araneae: Lycosidae) from Far East Asia. Zootaxa 4072(2): 263–281. doi:10.11646/zootaxa.4072.2.8
401. Nekhaeva AA, Marusik YM, Buckle DJ (2019) A survey of the Siberio-Nearctic

genus *Masikia* Millidge, 1984 (Aranei: Linyphiidae: Erigoninae). Arthropoda Selecta 28(1): 157–168. doi:10.15298/arthsel.28.1.15

402. Oliger TI, Marusik YM, Koponen S (2002) New and interesting records of spiders (Araneae) from the Maritime Province of Russia. Acta Arachnologica 51(2): 93–98. doi: 10.2476/asjaa.51.93
403. Omelko MM, Komisarenko AA, Marusik YM (2016) A new species of *Acantholycosa* Dahl, 1908 (Araneae: Lycosidae) from the Russian Far East. Zootaxa 4072(5): 596–600. doi:10.11646/zootaxa.4072.5.9
404. Omelko MM, Marusik YM (2008) A survey of the east Palearctic Lycosidae (Aranei). 4. On two somatically similar species of *Alopecosa* from the Russian Far East. Arthropoda Selecta 16(4): 237–243.
405. Omelko MM, Marusik YM (2012) A review of the *Haplodrassus montanus*-group (Aranei: Gnaphosidae) in the east Paleartic and preliminary grouping of the genus. Arthropoda Selecta 21(4): 339–348. doi:10.15298/arthsel.21.4.04
406. Omelko MM, Marusik YM (2013) A survey of east Palearctic Lycosidae (Araneae). 9. A review of *Sibirocosa* with a descriptions of three new species. Zootaxa 3666: 319–330. doi:10.11646/zootaxa.3666.3.2
407. Omelko MM, Marusik YM (2014) New data on spiders (Aranei) from southern part of the Khabarovsk Province. Arthropoda Selecta 23(3): 311–318. doi:10.15298/arthsel.23.3.10
408. Omelko MM, Marusik YM (2020) A new species and new records of wolf spiders (Araneae: Lycosidae) from Laos. Raffles Bulletin of Zoology 68: 479–487. doi:10.26107/RBZ–2020–0066
409. Omelko MM, Marusik YM (2021) New data on Linyphiidae spiders (Aranei) in the Maritime Province of Russia. Arthropoda Selecta 30(2): 245–256. doi:10.15298/arthsel.30.2.12
410. Omelko MM, Marusik YM, Koponen S (2011) A survey of the east Palearctic Lycosidae (Aranei). 8. The genera *Pirata* Sundevall, 1833 and *Piratula* Roewer, 1960 in the Russian Far East. Arthropoda Selecta 20(3): 195–232. doi:10.15298/arthsel.20.3.05

411. Omelko MM, Marusik YM, Koponen S (2013) A new species of *Alopecosa* Simon (Araneae, Lycosidae) from Sakhalin Island. Acta Zootaxonomica Sinica 38 (2): 267–271.
412. Omelko MM, Marusik YM, Kim BW (2013) Redescription of east Palearctic ground spider *Drassyllus biglobus* Paik, 1986 (Araneae: Gnaphosidae). Zootaxa 3686: 297–300. doi:10.11646/zootaxa.3686.2.10
413. Omelko MM, Marusik YM, Lyle R (2020) A survey of *Diphya* Nicolet, 1849 (Araneae: Tetragnathidae) from South Africa. Zootaxa 4899(1): 259–279. doi:10.11646/zootaxa.4899.1.14
414. Ono H, Marusik YM, Logunov DV (1990) Spiders of the family Thomisidae from Sakhalin and the Kurile Islands. Acta Arachnologica 39(1): 7–19. doi: 10.2476/asjaa.39.7
415. Ovtsharenko VI, Eskov KY, Marusik YM (1990) International Arachnological Congress in Finland. Proceeding of the Zoological Institute of the Academy of Sciences of USSR, Leningrad, 226: 138–141. [In Russian]
416. Ovtsharenko VI, Marusik YM (1988) Spiders of the family Gnaphosidae (Aranei) of the north-east of the USSR (the Magadan Province). Entomologicheskoe Obozrenie 67(4): 204–217. [In Russian]
417. Ovtsharenko VI, Marusik YM (1990) Taxonomical notes on spiders (Aranei), described by A. Grube. Proceeding of the Zoological Institute of the Academy of Sciences of USSR, Leningrad, 226: 70–73. [publ. in 1992] [In Russian]
418. Ovtsharenko VI, Marusik YM 1991 (1995) Additional data on the spiders of the family Gnaphosidae (Aranei) of the north-east Asia. In: Entomological Studies in the North-East of the USSR. USSR Academy of Sciences, Institute of Biological Problems of the North, Vladivostok 2: 114–130. (dated "1991," first distributed March, 1996) [In Russian]
419. Ovtsharenko VI, Platnick NI, Marusik YM (1995) A review of the Holarctic ground spider genus *Parasyrisca* (Araneae, Gnaphosidae). American Museum Novitates 3147: 1–55.
420. Özkütük RS, Elverici M, Marusik YM, Kunt KB (2015) A new species

of *Harpactea* Bristowe, 1939 from Turkey (Araneae: Dysderidae). Biodiversity Data Journal 3(e4419): 1–11. doi:10.3897/BDJ.3e4419

421. Özkütük RS, Marusik YM, Danışman T, Kunt, KB, Yağmur EA, Elverici M (2013). Genus *Scytodes* Latreille, 1804 in Turkey (Araneae, Scytodidae). Hacettepe Journal of Biology and Chemistry 41(1): 9–20.
422. Özkütük RS, Marusik YM, Elverici M, Kunt KB (2016). A new species of *Lathys* from Turkey (Araneae, Dictynidae). ZooKeys 632: 35–45. doi:10.3897/zookeys.632.10130
423. Özkütük RS, Marusik YM, Kunt KB, Danışman T (2011) New records for spider (Araneae) fauna of Turkey: *Paratrachelas maculatus* (Thorell, 1875) [Corinnidae], *Sintula retroversus* (O. P.-Cambridge, 1875) [Linyphiidae] and *Agroeca proxima* (O. P.-Cambridge, 1871) [Liocranidae]. Biological Diversity and Conservation 4(2): 224–232.
424. Özkütük RS, Marusik YM, Kunt, KB, Elverici M (2017) Taxonomic notes on two sibling species of *Metellina* from Asia (Araneae, Tetragnathidae). ZooKeys 695: 75–88. doi:10.3897/zookeys.695.13611
425. Penney D, Dunlop JA, Marusik YM (2012). Summary statistics for fossil spider species taxonomy. ZooKeys 192: 1–13. doi: 10.3897/zookeys.192.3093
426. Penney D, Green DI, McNeil A, Bradley R, Marusik YM, Withers PJ, Preziosi RF (2012) A new species of *Craspedisia* (Araneae: Theridiidae) in Miocene Dominican amber, imaged using X-ray computed tomography. Paleontological Journal 46: 583–588. doi: 10.1134/S0031030112060093
427. Penney D, Marusik YM, Wheater CP, Langan AM (2009) First Gambian Ricinulei and the northernmost African record for the order (Arachnida: Ricinulei: Ricinoididae). Zootaxa 2021: 66–68. doi: 10.11646/zootaxa.2021.1.5
428. Penney D, McNeil A, Green DI, Bradley R, Marusik YM, Withers PJ, Preziosi RF (2011) A new species of anapid spider (Araneae: Araneoidea, Anapidae) in Eocene Baltic amber, imaged using phase contrast X-ray computed micro-tomography. Zootaxa 2742: 60–66. doi: 10.11646/zootaxa.2742.1.4
429. Perkovsky EE, Eskov KY, Marusik YM (2017) First record of Atypidae (Araneae)

in Rovno Amber. Acta Arachnologica 67(1): 13–17. doi: 10.2476/asjaa.67.13

430. Potapov M, Marusik YM (2000) New and little known *Folsomia* (Collembola; Isotomidae) from South Kuriles. Russian Entomological Journal 9(2): 99–102.
431. Rossolimo TE, Marusik YM (1989) Cold resistance in some species of spiders of Magadan Region. Zoologicheskiĭ Zhurnal 68(3): 136–139. [In Russian]
432. Rybalov LB, Marusik YM, Tanasevitch AV, Vorobyeva IG, Koponen S (2002) Spiders (Aranei) of the Yenisei River middle flow and environs of "Mirnoye" field station. Study of biological diversity along Yenisei Ecological transect. Animal world. Moscow: IEEP: 70–95. [In Russian]
433. Saaristo MI, Marusik YM (2004) *Ferchestina*, a new genus of oonopid spiders from Russian Far East (Aranei, Oonopidae). Arthropoda Selecta 13(1): 51–54.
434. Saaristo MI, Marusik YM (2004) Revision of the Holarctic spider genus *Oreoneta* Kulczyński, 1894 (Arachnida: Araneae: Linyphiidae). Arthropoda Selecta 12(3–4, 2003): 185–220. [publ. July 16, 2004]
435. Saaristo MI, Marusik YM (2004). Two new petrophilous micronetine genera, *Agyphantes* gen. n. and *Lidia* gen. n. (Araneae, Linyphiidae, Micronetinae), from the eastern Palearctic with descriptions of two new species. Bulletin of the British Arachnological Society 13(3): 76–82.
436. Saaristo MI, Marusik YM (2008) A survey of African *Opopaea* Simon, 1891 (Arachnida, Aranei, Oonopidae). Arthropoda Selecta 17(1–2): 17–53.
437. Saaristo, M. I., Marusik YM (2009). A new genus and species of oonopid spider (Araneae, Oonopidae) from Ukraine. ZooKeys 24: 63–74. doi:10.3897/zookeys.24.278
438. Šestáková A, Marusik YM, Omelko MM (2014) A revision of the Holarctic genus *Larinioides* Caporiacco, 1934 (Araneae: Araneidae). Zootaxa 3894(1): 61–82. doi:10.11646/zootaxa.3894.1.6
439. Shafaie S, Mirshamsi O, Aliabadian M, Moradmand M, Marusik YM (2018) A new *Pardosa* species from northern Iran (Araneae, Lycosidae). Zootaxa 4387(2): 350–364. doi:10.11646/zootaxa.4387.2.6
440. Shafaie S, Mirshamsi O, Aliabadian M, Moradmand M, Marusik YM (2018)

Pardosa colchica Mcheidze, 1946: a first report from West Azerbaijan Province, Iran (Araneae: Lycosidae). Iranian Journal of Animal Biosystematics 13(2, 2017): 199–206. doi:10.22067/ijab.v13i2.70021 [publ. in July 2018]

441. Sket B, Stoev P, Deltshev C, Marusik Y, Deharveng L (2019). Cave fauna. In: Welch G & Stoev P (Eds.). A report of RSPB-supported scientific research at Koytendag State Nature Reserve, East Turkmenistan. Avdanced Books. Pensoft Publishers, Sofia, 39-43. https://doi.org/10.3897/ab.e47154 [In Russian]
442. Sket B, Stoev P, Deltshev C, Marusik YM, Deharveng L (2019) Cave fauna. In: A report of RSPB-supported scientific research at Koytendag State Nature Reserve, East Turkmenistan. Sofia: Pensoft Publishers, 37–41.
443. Snegovaya NY, Marusik YM (2012) New species and collections of Opiliones (Arachnida) from Turkey. Acta Arachnologica 61(2): 59–70. doi: 10.2476/asjaa.61.59
444. Song YJ, Li SQ, Marusik YM (2006) Redescription on *Dicymbium facetum* (L. Koch) (Araneae, Linyphiidae) with first report on its male. Acta Zootaxonomica Sinica 31(2): 330–334.
445. Stoev P, Deltshev C, Marusik YM, Fet V, Kovařík F, Guéorguiev B, Dedov I, Ljubomirov T, Chobanov D, Menliev S (2019) Surface-dwelling invertebrates. In: A report of RSPB-supported scientific research at Koytendag State Nature Reserve, East Turkmenistan (Vol. 1). Pensoft Publishers, Sofia, 31–36.
446. Stoev P, Deltshev C, Marusik YM, Fet V, Kovařík F, Guéorguiev B, Dedov I, Ljubomirov T, Chobanov D, Menliev S (2019) Surface-dwelling invertebrates. In: Welch G & Stoev P (Eds.). A report of RSPB-supported scientific research at Koytendag State Nature Reserve, East Turkmenistan. Avdanced Books. Pensoft Publishers, Sofia, 31-36. https://doi.org/10.3897/ab.e37858 [In Russian]
447. Sun N, Marusik YM, Tu LH (2014) *Acanoides* gen. n., a new spider genus from China with a note on the taxonomic status of *Acanthoneta* Eskov & Marusik, 1992 (Araneae, Linyphiidae, Micronetinae). ZooKeys 375: 75–99. doi:10.3897/zookeys.375.6116
448. Takagi S, Marusik YM, Ohara M, Urbain B (1997) Records of *Arctorthezia*

cataphracta from the Middle Kurile Islands and SEM observations on wax-secreting organs (Homoptera: Coccoidea: Ortheziidae). Bulletin of the Otaru Museum 10: 1–7.

449. Tanasevitch AV, Marusik YM (2019) A new *Gongylidioides* Oi, 1960 from Taiwan (Aranei: Linyphiidae). Arthropoda Selecta 28(1): 152–156. doi:10.15298/arthsel.28.1.14
450. Tanasevitch AV, Marusik YM (2020) A new species of the genus *Carniella* Thaler & Steinberger from the Nepal Himalayas (Araneae: Theridiidae). Pakistan Journal of Zoology 52(6): 2275–2280. doi:10.17582/journal.pjz/20180402170422
451. Tang J, Xu X, Yin HQ, Marusik YM, Huang Z (2020) Redescription of holotypes of four *Alopecosa* species (Araneae, Lycosidae) from China. ZooKeys 945, 85–97. doi: 10.3897/zookeys.945.52287
452. Tyshchenko VP, Marusik YM (1985) Catching webs of orb-weaving spiders. 3. Geographic variations of webs in *Araneus marmoreus* (Aranei, Araneidae). Zoologicheskiĭ Zhurnal 64(12): 1816–1822. [In Russian]
453. Tyshchenko VP, Marusik YM, Tarabayev CK (1985) Catching webs of orb-weaving spiders. 2. Comparative study of the webs in the genus *Nuctenea* (Aranei, Araneidae). Zoologicheskiĭ Zhurnal 64(6): 827–833. [In Russian]
454. Wang LY, Marusik YM, Zhang ZS (2012) Notes on three poorly known *Arctosa* species from China (Araneae: Lycosidae). Zootaxa 3404: 53–68. doi:10.11646/zootaxa.3404.1.2
455. Wang Y, Wan N, Tong YF, Marusik YM (2021) On the northernmost *Orchestina* species in China, with a first description of the female of *O. zhiwui* (Araneae, Oonopidae). ZooKeys 1022: 1–11. doi:10.3897/zookeys.1022.62387
456. Wesołowska W, Marusik YM (1990) Notes on *Heliophanus camtschadalicus* Kulczyński, 1885 (Aranei, Salticidae) and the related species. Korean Arachnology 6(1): 91–100.
457. Yoshizawa K, Marusik YM, Yao I, Lienhard C (2020) Systematic Position of the Enigmatic Psocid Family Lesneiidae (Insecta: Psocodea: Psocomorpha), With

Description of Two New Species. Insect Systematics and Diversity 4(6):1–6. doi: 10.1093/isd/ixaa019

458. Zamani A, Chatzaki M, Esyunin SL, Marusik YM (2021) One new genus and nineteen new species of ground spiders (Araneae: Gnaphosidae) from Iran, with other taxonomic considerations. European Journal of Taxonomy 751: 68–114. doi:10.5852/ejt.2021.751.1381
459. Zamani A, Dimitrov D, Weiss I, Alimohammadi S, Rafiei-Jahed R, Esyunin SL, Moradmand M, Chatzaki M, Marusik YM (2020) New data on the spider fauna of Iran (Arachnida: Araneae), part VII. Arachnology 18(6): 569–591. doi:10.13156/arac.2020.18.6.569
460. Zamani A, Marusik YM (2016) A new species and new distribution records of *Zaitunia* from Iran (Araneae: Filistatidae). Zoology in the Middle East 62(4): 373–376. doi: 10.1080/09397140.2016.1250712
461. Zamani A, Marusik YM (2017) Six new species of spiders (Arachnida: Araneae) from Iran. Oriental Insects 51(4): 313–329. doi:10.1080/00305316.2017.1282386
462. Zamani A, Marusik YM (2018) A new species of the hersiliid spiders (Aranei: Hersiliidae) from Iran. Eurasian Entomological Journal 17(4), 273–275. doi: 10.15298/euroasentj.17.4.05
463. Zamani A, Marusik YM (2018). New species and records of Filistatidae (Arachnida: Aranei) from Iran. Arthropoda Selecta 27(2): 121–128. doi:10.15298/arthsel.27.2.03
464. Zamani A, Marusik YM (2018) The first report on the spider fauna (Arachnida: Araneae) of the Lut Desert, Iran. Acta Arachnologica 67(2), 67–75. doi: 10.2476/asjaa.67.67
465. Zonstein SL, Marusik YM (2019) A revision of the spider genus *Filistata* (Araneae: Filistatidae). Arachnology 18(2): 53-93. doi:10.13156/arac.2018.18.2.53
466. Zamani A, Marusik YM (2019) The spider genera *Azerithonica* and *Tegenaria* (Aranei: Agelenidae: Tegenariini) in Iran. Arthropoda Selecta 28(2): 291–303. doi:10.15298/ arthsel.28.2.12
467. Zamani A, Marusik YM (2020) A new and easternmost species

of *Loureedia* (Aranei: Eresidae) from Iran. Arthropoda Selecta 29(2): 239–243. doi:10.15298/arthsel.29.2.09

468. Zamani A, Marusik YM (2020) A review of Agelenini (Araneae: Agelenidae: Ageleninae) of Iran and Tajikistan, with descriptions of four new genera. Arachnology 18(4): 368–386. doi:10.13156/arac.2020.18.4.368
469. Zamani A, Marusik YM (2020) A survey of Phrurolithidae (Arachnida: Araneae) in southern Caucasus, Iran and Central Asia. Zootaxa 4758(2): 311–329. doi:10.11646/zootaxa.4758.2.6
470. Zamani A, Marusik YM (2020) New species of Filistatidae, Palpimanidae and Scytodidae (Arachnida: Araneae) from southern Iran. Acta Arachnologica 69(2): 121–126. doi:10.2476/asjaa.69.121
471. Zamani A, Marusik YM (2020) Two new species of *Araniella* (Aranei: Araneidae) from Western Himalaya, with notes on species reported from India. Arthropoda Selecta 29(3): 361–366. doi:10.15298/arthsel.29.3.09
472. Zamani A, Marusik YM (2021) A new genus and ten new species of spiders (Arachnida, Araneae) from Iran. ZooKeys 1054: 95–126. doi:10.3897/zookeys.1054.70408
473. Zamani A, Marusik YM (2021) New taxa of six families of spiders (Arachnida: Araneae) from Iran. Zoology in the Middle East 67(1): 81–91. doi:10.1080/09397140.2021.1877382
474. Zamani A, Marusik YM (2021) Revision of the spider family Zodariidae (Arachnida, Araneae) in Iran and Turkmenistan, with seventeen new species. ZooKeys 1035: 145–193. doi:10.3897/zookeys.1035.65767
475. Zamani A, Marusik YM (2021) Three new species of spiders (Aranei) from Iran. Caucasian Entomological Bulletin 17(2): 451–458. doi: 10.23885/181433262021172-451458
476. Zamani A, Marusik YM (2021) Two new species of Liocranidae (Arachnida: Aranei) from the Caucasus and northern Iran. Arthropoda Selecta 30(4): 557–564. doi:10.15298/arthsel.30.4.12
477. Zamani A, Marusik YM (2021) Two new species of Theridiidae from Iran, and the

revalidation of *Enoplognatha submargarita* Yaginuma & Zhu, 1992 (Arachnida: Araneae). Arachnology 18(8): 957–964. doi:10.13156/arac.2021.18.8.9570

478. Zamani A, Marusik YM, Berry JW (2016) A new species of *Paratheuma* (Araneae: Dictynidae) from Southwestern Asia and transfer of the genus. Zoology in the Middle East 62(2): 177–183. doi:10.1080/09397140.2016.1173927
479. Zamani A, Marusik YM, Koponen S (2017) First description of the male of the easternmost *Harpactea* species, *H. parthica* (Araneae: Dysderidae). Zootaxa 4238(2): 258–262. doi:10.11646/zootaxa.4238.2.6
480. Zamani A, Marusik YM, Malek-Hosseini MJ (2018) A new species of *Tegenaria* Latreille, 1804 (Araneae: Agelenidae) from western Iran. Zootaxa 4444(1): 95–97. doi:10.11646/zootaxa.4444.1.7
481. Zamani A, Marusik YM, Šestáková A (2020) On *Araniella* and *Neoscona* (Araneae, Araneidae) of the Caucasus, Middle East and Central Asia. ZooKeys 906: 13–40. doi:10.3897/zookeys.906.47978
482. Zamani A, Marusik YM, Soofi M, Koponen S, Caleb JTD., Šestáková A (2019). First record of *Poltys nagpurensis* (Araneae: Araneidae) from Iran. Arachnologische Mitteilungen 57(1): 4–7. doi:10.30963/aramit5702
483. Zamani A, Mirshamsi O, Dolejš P, Marusik YM, Esyunin SL, Hula V, Ponel P (2017) New data on the spider fauna of Iran (Arachnida: Araneae), part IV. Acta Arachnologica 66(2): 55–71. doi:10.2476/asjaa.66.55
484. Zamani A, Mirshamsi O, Jannesar B, Marusik YM, Esyunin SL (2015) New data on spider fauna of Iran (Arachnida: Araneae), Part II. Zoology and Ecology 25(4): 339–346. doi:10.1080/21658005.2015.1068508
485. Zamani A, Mirshamsi O, Marusik YM (2017) Description of a new species of *Hersiliola* and the male of *Duninia rheimsae* Marusik & Fet, 2009 from Iran (Araneae: Hersiliidae). Turkish Journal of Zoology 41(4): 624–629. doi:10.3906/zoo–1609–53
486. Zamani A, Mirshamsi O, Marusik Y (2021) 'Burning Violin': The Medically Important Spider Genus *Loxosceles* (Araneae: Sicariidae) in Iran, Turkmenistan, and Afghanistan, With Two New Species. Journal of Medical Entomology 58(2),

666–675. doi: 10.1093/jme/tjaa257

487. Zamani A, Mirshamsi O, Marusik YM, Hatami M, Maddahi H (2017). The spider genus *Oecobius* in Iran, with description of two new species (Araneae: Oecobiidae). Oriental Insects 51(4): 330–337. doi:10.1080/00305316.2017.1283257
488. Zamani A, Mirshamsi O, Rashidi P, Marusik YM, Moradmand M, Bolzern A (2016) New data on the spider fauna of Iran (Arachnida: Aranei), part III. Arthropoda Selecta 25(1): 99–114. doi:10.15298/arthsel.25.1.10
489. Zamani A, Nadolny AA, Esyunin SL, Marusik YM (2021) New data on the spider fauna of Iran (Arachnida: Araneae), part VIII. Zoosystematica Rossica 30(2): 279–297. doi:10.31610/zsr/2021.30.2.279
490. Zamani A, Seiedy M, Saboori A, Marusik YM (2018) The spider genus *Pterotricha* Kulczyński, 1903 (Araneae: Gnaphosidae) in Iran, with the description of a new genus. Zookeys 777: 17–41. doi: 10.3897/zookeys.777.26745
491. Zamani A, Tanasevitch AV, Nadolny AA, Esyunin SL, Marusik YM (2019) New data on the spider fauna of Iran (Arachnida: Aranei). Part VI. Euroasian Entomological Journal 18(4), 233–243. doi: 10.15298/euroasentj.18.4.01
492. Zhang JS, Marusik YM, Oketch DA, Kioko EN, Yu H, Li SQ (2021) Resurrection of the spider genus *Bucliona* Benoit, 1977, with a description of a new species from Kenya (Araneae, Clubionidae). Zootaxa 5006(1): 195–207. doi:10.11646/zootaxa.5006.1.21
493. Zhang XQ, Marusik YM (2016) A survey of *Pireneitega* from Tajikistan (Agelenidae, Coelotinae). ZooKeys 635: 89–107. doi:10.3897/zookeys.635.10487
494. Zheng G, Marusik YM, Li SQ (2009) Discovery of Stenochilidae Thorell, 1873 (Araneae) in China, with description of a new species from Yunnan. Revue Suisse de Zoologie 116(2): 303–311. doi:10.5962/bhl.part.79498
495. Zonstein SL, Marusik YM (2010) *Raveniola niedermeyeri* from Iran: redescription and new data on distribution (Araneae, Nemesiidae). ZooKeys 57: 51–57. doi:10.3897/zookeys.57.497
496. Zonstein SL, Marusik YM (2012) A review of the genus *Raveniola* (Araneae,

Nemesiidae) in China, with notes on allied genera and description of four new species from Yunnan. ZooKeys 211: 71–99. doi:10.3897/zookeys.211.3060
497. Zonstein SL, Marusik YM (2013) Checklist of the spiders (Araneae) of Israel. Zootaxa 3671: 1–127. doi: 10.11646/zootaxa.3671.1.1
498. Zonstein, SL, Marusik YM (2013) On *Levymanus*, a remarkable new spider genus from Israel, with notes on the Chediminae (Araneae, Palpimanidae). ZooKeys 326: 27–45. doi:10.3897/zookeys.326.5344
499. Zonstein SL, Marusik YM (2014) A redescription of *Damarchus cavernicola* Abraham, 1924, with notes on *Damarchus* Thorell, 1891 and *Atmetochilus* Simon, 1887 (Aranei: Nemesiidae). Arthropoda Selecta 23(3): 273–278. doi:10.15298/arthsel.23.3.06
500. Zonstein SL, Marusik YM (2015) The first record of *Andoharano* Lehtinen, 1967 (Araneae: Filistatidae) from mainland Africa. African Invertebrates 56(2): 483–489. doi:10.5733/afin.056.0217
501. Zonstein SL, Marusik YM (2016) A review of the spider genus *Atmetochilus* of Sumatra, Indonesia, with first analysis of male characters and description of three new species (Araneae, Nemesiidae). Zoological Studies 55(10): 1–17. doi:10.6620/ZS.2016.55–10
502. Zonstein SL, Marusik YM (2016) A revision of the spider genus *Zaitunia* (Araneae, Filistatidae). European Journal of Taxonomy 214: 1–97. doi:10.5852/ejt.2016.214
503. Zonstein SL, Marusik YM (2017) A redescription of *Chedima purpurea* Simon, 1873, with notes on the unique copulative stopper mechanism in females (Aranei: Palpimanidae). Arthropoda Selecta 26(3): 225–232. doi:10.15298/arthsel.26.3.03
504. Zonstein SL, Marusik YM (2017) Descriptions of the two-eyed African spider genera *Chedimanops* gen. n. and *Hybosidella* gen. n. (Araneae, Palpimanidae, Chediminae). African Invertebrates 58(1): 23–47. doi:10.3897/AfrInvertebr.58.11448
505. Zonstein SL, Marusik YM (2019) On the revisited types of four poorly known African species of *Palpimanus* (Araneae, Palpimanidae). African Invertebrates 60(1): 83–95. doi:10.3897/AfrInvertebr.60.34229

506. Zonstein SL, Marusik YM (2020) A review of the spider genus *Boagrius* Simon, 1893 (Araneae: Palpimanidae). Raffles Bulletin of Zoology 68: 91–102. doi:10.26107/RBZ–2020–0010
507. Zonstein SL, Marusik YM (2020) Two new species of *Diaphorocellus* Simon, 1893 from Madagascar (Araneae, Palpimanidae). African Invertebrates 61(1): 1–15. doi:10.3897/AfrInvertebr.61.47048
508. Zonstein SL, Marusik YM (2021) The first Western Palearctic record of *Euprosthenops* Pocock (Araneae, Pisauridae), with description of a new species from Israel. ZooKeys 1065: 13–27. doi: 10.3897/zookeys.1065.74119
509. Zonstein SL, Marusik YM, Grabolle A (2018) A remarkably small new species of *Filistata* (Aranei: Filistatidae) from Portugal. Arthropoda Selecta 27(1): 49–52. doi:10.15298/arthsel.27.1.06
510. Zonstein SL, Marusik YM, Koponen S (2013) Redescription of three species of Filistatidae (Araneae) described by C.F. Roewer from Afghanistan. Zootaxa 3745(1): 64–72. doi:10.11646/zootaxa.3745.1.5
511. Zonstein SL, Marusik YM, Koponen S (2018) A redescription of *Ancylotrypa elongata* Purcell, 1908, with first description of the male (Aranei: Cyrtaucheniidae). Arthropoda Selecta 27(3): 227–231. doi:10.15298/arthsel.27.3.05
512. Zonstein SL, Marusik YM, Kovblyuk MM (2017) New data on the spider genus *Levymanus* (Araneae: Palpimanidae). Oriental Insects 51(3): 221–226. doi:10.1080/00305316.2016.1275989
513. Zonstein SL, Marusik YM, Magalhaes ILF (2017) *Labahitha* nom.n., a replacement name for *Mystes* Bristowe, 1938, with a redescription of the type species (Aranei: Filistatidae). Arthropoda Selecta 26(4): 303–309. doi:10.15298/arthsel.26.4.04
514. Zonstein SL, Marusik YM, Omelko MM (2015) A survey of spider taxa new to Israel (Arachnida: Araneae). Zoology in the Middle East 61(4): 372–385. doi:10.1080/09397140.2015.1095525
515. Zonstein SL, Marusik YM, Omelko MM (2016) A redescription of the type species

of *Tricalamus* Wang 1987 (Aranei, Filistatidae). Zoologicheskiĭ Zhurnal 95(5): 540–544. doi:10.7868/S0044513416050159

516. Zonstein SL, Marusik YM, Omelko MM (2016). Redescription of the type species of *Diaphorocellus* Simon, 1893 (Araneae, Palpimanidae, Chediminae). African Invertebrates 57(2): 93–103. doi:10.3897/AfrInvertebr.57.9988
517. Zonstein SL, Marusik YM, Omelko MM (2018) Redescription of the monotypic genus *Scelidomachus* Pocock, 1899 (Aranei: Palpimanidae) and its type species. Arthropoda Selecta 27(1): 53–56. doi: 10.15298/arthsel.27.1.07
518. Zyuzin AA, Marusik YM (1988) A new species of spiders of the genus *Acantholycosa* (Aranei, Lycosidae) from the east Siberia. Zoologicheskiĭ Zhurnal 67(7): 1083–1085. [In Russian]
519. Zyuzin AA, Marusik YM (1989) A new species of spiders of the genus *Pardosa* C. L. Koch (Araneae, Lycosidae) from Magadan Province. Entomologicheskoe Obozrenie 68(2): 432–434. [In Russian]

List 1B: Anniversaries and celebrations (arranged in chronological order):

1. Marusik YM (2004) Pavel Iustinovich Marikovskiy – 75 years on service of science and community. Arthropoda Selecta 12(3–4): 255–258. [in Russian]
2. Marusik YM (2004) Towards the 70th birthday of P.T. Lehtinen, prominent arachnologist and revolutionary of spider taxonomy. Arthropoda Selecta 13(1–2): 87–92. [in Russian]
3. Marusik YM (2004) Towards the 60th birthday of Seppo Koponen, the greater friend of Russian arachnology. Arthropoda Selecta 13(3): 171–177. [in Russian]
4. Marusik YM (2007) Arachnologist, paleontologist, writer, publicist Kirill Yurievich Eskov – towards the 50th birthday. Arthropoda Selecta 15(3): 181–187. [in Russian]
5. Marusik YM (2007) Towards the 50th birthday of Andrei Viktorovich Tanasevitch, the most productive researcher of Linyphiidae in the World. Arthropoda Selecta 15(3): 188–192. [in Russian]

6. Marusik YM (2010) [Names in Russian arachnology] Adolph Eduard Grube 1812–1880. Arthropoda Selecta 19(2): 109–111. [in Russian]
7. Marusik YM, Logunov DV (2011) Our colleague Kirill Mikhailov, arachnologist and publisher – towards the 50th birthday. Arthropoda Selecta 20(3): 241–248. [in Russian]
8. Mikhailov KG, Marusik YM (2011) Dmitry Viktorovich Logunov – towards the 50th birthday. Arthropoda Selecta 21(2): 183–186. [in Russian]
9. Marusik YM, Alfimov AV (2012) 75 years to Daniil Iosifovich Berman. Zoologica Journal 91(9): 1147–1149. [in Russian]
10. Marusik YM, Alfimov AV (2012) 75 years to professor Daniil Iosifovich Berman. Eurasian Entomological Journal 11 (suppl. 1): 3–10 [in Russian]
11. Logunov DV, Marusik YM (2012) Famous Swedish arachnologist Torbjorn Kronestedt – towards the 70th birthday. Arthropoda Selecta 21(4): 379–385. [in Russian]
12. Marusik YM, Koponen S (2014) Pekka T. Lehtinen: A biography to mark his 80th birthday. Arthropoda Selecta 23(2): 89–96.
13. Marusik YM, Fet V (2014). Honouring Seppo Koponen on the occasion of this 70th Birthday. Zootaxa 3894 (1): 5–9. doi.org/10.11646/zootaxa.3894.1.3
14. Marusik YM (2014) Jörg Wunderlich wird 75 Jahre alt. Arachnologische Mitteilungen 48: iii-vii.

List 1C: Obituaries (arranged in chronological order):

1. Marusik YM, Golovatch SI (1993) Boris Pimenovich Chevrizov (1951-1993). Arthropoda Selecta 2(3): 77–78. [in Russian]
2. Marusik YM (1994) Pontus Palmgren (27.04.1907–26.11.1993). Arthropoda Selecta 3(1–2): 133–134. [in Russian]
3. Marusik YM (1996) Takeo Yaginuma 1916–1995. Arthropoda Selecta 5(1–2): 150. [in Russian]

4. Marusik YM, Otto S (2008) 70 years of Arachnology in Georgia: Tamara S. Mkheidze (1915-2007). Newsletter of the British Arachnological Society 112: 6–8.
5. Marusik YM (2008) Tamara Severianovna Mkheidze 1915–2007. Arthropoda Selecta 16(3): 191–194. [in Russian]
6. Marusik YM (2008) Michael Ilmari Saaristo (1938–2008). Arthropoda Selecta 16(4): 251–257. [in Russian]
7. Marusik YM & Кoponen S (2008) Michael Ilmari Saaristo (1938–2008). Arthropoda Selecta 17(1–2): 4–16. [in Russian]
8. Marusik YM (2008) Daxiang Song (=Tahsiang Sung) 9.05.1935–25.01.2008. Arthropoda Selecta 17(3–4): 211–213. [in Russian]
9. Marusik YM & Кoponen S (2008) Michael Ilmari Saaristo (1938–2008). Arachnologische Mitteilungen 36(4): 37–40.
10. Marusik YM (2009) Gershom Levy (1937–2009). Arthropoda Selecta 18(3–4): 189–191. [in Russian]
11. Marusik YM (2016) Robin Ernest Leech, 1.02.1937–17.06.2016. Arthropoda Selecta 25(4): 427–430. [in Russian]
12. Marusik YM (2020) Norman Ira Platnick (1951–2020). Arthropoda Selecta 29(2): 293–296. [in Russian]

List 2 List of the taxa described by Yuri M. Marusik

CLASS ARACHNIDA LAMARCK, 1801

ORDER ARANEAE CLERCK, 1757

SUBORDER OPISTHOTHELAE POCOCK, 1892

INFRAORDER ARANEOMORPHAE POCOCK, 1892

Family Agelenidae C. L. Koch, 1837

Asiascape Zamani & Marusik, 2020 [type species: *Asiascape parthica* Zamani & Marusik, 2020]

Asiascape parthica Zamani & Marusik, 2020

Azerithonica Guseinov, Marusik & Koponen, 2005 [type species: *Azerithonica hyrcanica* Guseinov, Marusik & Koponen, 2005]

Azerithonica hyrcanica Guseinov, Marusik & Koponen, 2005

Azerithonica sagartia Zamani & Marusik, 2019

Draconarius latellai Marusik & Ballarin, 2011

Gorbiscape Zamani & Marusik, 2020 [type species: *Gorbiscape gorbachevi* Zamani & Marusik, 2020]

Gorbiscape gorbachevi Zamani & Marusik, 2020

Lycosoides lehtineni Marusik & Guseinov, 2003

Persilena Zamani & Marusik, 2020 [type species: *Persilena sengleti* Zamani & Marusik, 2020]

Persilena sengleti Zamani & Marusik, 2020

Persiscape Zamani & Marusik, 2020 [type species: *Agelescape levyi* Guseinov, Marusik & Koponen, 2005 = *Persiscape levyi* (Guseinov, Marusik & Koponen, 2005)]

Persiscape caspica Zamani & Marusik, 2020

Persiscape caucasica (Guseinov, Marusik & Koponen, 2005) [= *Agelescape caucasica* Guseinov, Marusik & Koponen, 2005]

Persiscape dunini (Guseinov, Marusik & Koponen, 2005) [= *Agelescape dunini* Guseinov, Marusik & Koponen, 2005 synonym of *Persiscape gideoni* (Levy, 1996)]

Persiscape ecbatana Zamani & Marusik, 2020

Persiscape levyi (Guseinov, Marusik & Koponen, 2005) = [*Agelescape levyi* Guseinov, Marusik & Koponen, 2005]

Persiscape nassirkhanii Zamani & Marusik, 2020

Persiscape talyshica (Guseinov, Marusik & Koponen, 2005) [= *Agelescape talyshica* Guseinov, Marusik & Koponen, 2005 synonym of *Persiscape levyi* (Guseinov, Marusik & Koponen, 2005)]

Persiscape zagrosensis Zamani & Marusik, 2020

Pireneitega kovblyuki Zhang & Marusik, 2016

Pireneitega muratovi Zhang & Marusik, 2016

Pireneitega ovtchinnikovi Kovblyuk, Kastrygina, Marusik & Ponomarev, 2013

Pireneitega ramitensis Zhang & Marusik, 2016

Pireneitega sakhalinensis (Marusik & Logunov, 1991) [= *Coelotes sakhalinensis* Marusik & Logunov, 1991 synonym of *Pireneitega luctuosa* (L. Koch, 1878)]

Pireneitega tyurai Zhang & Marusik, 2016

Pireneitega zonsteini Zhang & Marusik, 2016

Tegenaria adomestica Guseinov, Marusik & Koponen, 2005

Tegenaria alamto Zamani, Marusik & Malek-Hosseini, 2018

Tegenaria arsacia Zamani & Marusik, 2019

Tegenaria bayrami Kaya, Kunt, Marusik & Uğurtaş, 2010

Tegenaria daylamanica Zamani & Marusik, 2019

Tegenaria eros Zamani & Marusik, 2019

Tegenaria guseinovi Zamani & Marusik, 2019

Tegenaria halidi Guseinov, Marusik & Koponen, 2005

Tegenaria ismaillensis Guseinov, Marusik & Koponen, 2005

Tegenaria lehtineni (Guseinov, Marusik & Koponen, 2005) [= *Malthonica lehtineni* Guseinov, Marusik & Koponen, 2005]

Tegenaria lenkoranica (Guseinov, Marusik & Koponen, 2005) [= *Malthonica lenkoranica* Guseinov, Marusik & Koponen, 2005]

Tegenaria nakhchivanica (Guseinov, Marusik & Koponen, 2005) [= *Malthonica nakhchivanica* Guseinov, Marusik & Koponen, 2005]

Tegenaria pseudolyncea (Guseinov, Marusik & Koponen, 2005) [= *Malthonica pseudolyncea* Guseinov, Marusik & Koponen, 2005]

Tegenaria rahnamayi Zamani & Marusik, 2019

Tegenaria shirin Zamani & Marusik, 2019

Tegenaria talyshica Guseinov, Marusik & Koponen, 2005

Tegenaria zagatalensis Guseinov, Marusik & Koponen, 2005

Tegenaria zamanii Marusik & Omelko, 2014

Family Amaurobiidae Thorell,1870

Amaurobius antipovae Marusik & Kovblyuk, 2004

Amaurobius caucasicus Marusik, Otto & Japoshvili, 2020

Amaurobius koponeni Marusik, Ballarin & Omelko, 2012 [synonym of *Amaurobius jugorum* L. Koch, 1868]

Ecurobius Zamani & Marusik, 2021 [type species: *Ecurobius parthicus* Zamani & Marusik, 2021]

Ecurobius parthicus Zamani & Marusik, 2021

Ovtchinnikoviinae Marusik, Kovblyuk & Ponomarev, 2010 [subfamily, type genus:*Ovtchinnikovia* Marusik, Kovblyuk & Ponomarev, 2010]

Ovtchinnikovia Marusik, Kovblyuk & Ponomarev, 2010 [type species: *Ovtchinnikovia caucasica* Marusik, Kovblyuk & Ponomarev, 2010]

Ovtchinnikovia caucasica Marusik, Kovblyuk & Ponomarev, 2010

Family Anapidae Simon, 1895

Balticoroma wheateri Penney & Marusik, 2011 [fossil]

Family Araneidae Clerck, 1757

Araniella levii Zamani & Marusik, 2020

Araniella maasdorpi Zamani & Marusik, 2020

Araniella mithra Zamani, Marusik & Šestáková, 2020

Araniella villanii Zamani, Marusik & Šestáková, 2020

Cercidia levii Marusik, 1985

Larinia bossae Marusik, 1987

Larinia jeskovi Marusik, 1987

Larinia nenilini Marusik, 1986 [synonym of *Larinia phthisica* (L. Koch, 1871)]

Neoscona isatis Zamani, Marusik & Šestáková, 2020

Family Cheiracanthiidae Wagner, 1887

Cheiracanthium vankhedei Marusik & Fomichev, 2016

Family Clubionidae Wagner, 1887

Clubiona kularensis Marusik & Koponen, 2002

Porrhoclubiona bosmansi Marusik & Omelko, 2018

Porrhoclubiona moradmandi Marusik & Omelko, 2018

Family Ctenidae Keyserling, 1877

Sinoctenus Marusik, Zhang & Omelko, 2012 [type species: *Sinoctenus zhui* Marusik, Zhang & Omelko, 2012]

Sinoctenus zhui Marusik, Zhang & Omelko, 2012

Family Cybaeidae Banks, 1892

Cybaeota wesolowskae Marusik, Omelko & Koponen, 2020

Cybaeus bam Marusik & Logunov, 1991

Cybaeus basarukini Marusik & Logunov, 1991

Cybaeus kunashirensis Marusik & Logunov, 1991

Cybaeus mikhailovi Marusik & Omelko, 2021

Paracedicus feti Marusik & Guseinov, 2003

Paracedicus kasatkini Zamani & Marusik, 2017

Family Dictynidae O. Pickard-Cambridge, 1871

Ajmonia lehtineni Marusik & Koponen, 1998

Ajmonia rajaeii Zamani & Marusik, 2017

Brigittea avicenna Zamani & Marusik, 2021

Devade mongolica Esyunin & Marusik, 2001

Devade naderii Zamani & Marusik, 2017

Dictyna obydovi Marusik & Koponen, 1998

Dictyna ottoi Marusik & Koponen, 2017

Dictyna palmgreni Marusik & Fritzén, 2011

Dictyna tyshchenkoi Marusik, 1988

Dictyna tyshchenkoi wrangeliana Marusik, 1988

Dictyna ubsunurica Marusik & Koponen, 1998

Dictyna uvs Marusik & Koponen, 1998

Dictynomorpha daemonis Marusik, Esyunin & Tuneva, 2015

Emblyna budarini Marusik, 1988

Emblyna kaszabi Marusik & Koponen, 1998

Emblyna logunovi Marusik & Koponen, 1998 [synonym of *Emblyna wangi* (Song & Zhou, 1986)]

Emblyna mongolica Marusik & Koponen, 1998

Emblyna zherikhini (Marusik, 1988) [= *Dictyna zherikhini* Marusik, 1988]

Lathys ankaraensis Özkütük, Marusik, Elverici & Kunt, 2016

Lathys bin Marusik & Logunov, 1991

Paratheuma enigmatica Zamani, Marusik & Berry, 2016

Tricholathys ovtchinnikovi Marusik, Omelko & Ponomarev, 2017

Family Dysderidae C. L. Koch, 1837

Dysdera mikhailovi Fomichev & Marusik, 2021

Harpactea alanyana Özkütük, Elverici, Marusik & Kunt, 2015

Harpactea karaschkhan Kunt, Özkütük, Elverici, Marusik & Karakaş, 2016

Hygrocrates kovblyuki Kunt & Marusik, 2013

Family Eresidae C. L. Koch, 1845

Eresus lishizheni Lin, Marusik & Li, 2021

Loureedia phoenixi Zamani & Marusik, 2020

Family Filistatidae Simon, 1864

Andoharano ansieae Zonstein & Marusik, 2015

Filistata albens Zonstein & Marusik, 2019

Filistata balouchi Zamani & Marusik, 2020

Filistata lehtineni Marusik & Zonstein, 2014

Filistata lubinae Zonstein & Marusik, 2019

Filistata maguirei Marusik & Zamani, 2015

Filistata pygmaea Zonstein, Marusik & Grabolle, 2018

Filistata wunderlichi Zonstein & Marusik, 2019

Labahitha Zonstein, Marusik & Magalhaes, 2017 [type species:*Mystes oonopiformis* Bristowe, 1938 = *Labahitha oonopiformis* (Bristowe, 1938)]

Microfilistata magalhaesi Zamani & Marusik, 2018

Pritha garfieldi Marusik & Zamani, 2015

Sahastata amethystina Marusik & Zamani, 2016

Sahastata bosmansi Zonstein & Marusik, 2019

Sahastata sinuspersica Marusik, Zamani & Mirshamsi, 2014

Sahastata wesolowskae Magalhaes, Stockmann, Marusik & Zonstein, 2020

Sahastata wunderlichi Magalhaes, Stockmann, Marusik & Zonstein, 2020

Zaitunia akhanii Marusik & Zamani, 2015

Zaitunia brignoliana Zonstein & Marusik, 2016

Zaitunia darreshurii Zamani & Marusik, 2018

Zaitunia ferghanensis Zonstein & Marusik, 2016

Zaitunia feti Zonstein & Marusik, 2016

Zaitunia halepensis Zonstein & Marusik, 2016

Zaitunia huberi Zonstein & Marusik, 2016

Zaitunia kunti Zonstein & Marusik, 2016

Zaitunia logunovi Zonstein & Marusik, 2016

Zaitunia minoica Zonstein & Marusik, 2016

Zaitunia minuta Zonstein & Marusik, 2016

Zaitunia psammodroma Zonstein & Marusik, 2016

Zaitunia spinimana Zonstein & Marusik, 2016

Zaitunia vahabzadehi Zamani & Marusik, 2016

Zaitunia wunderlichi Zonstein & Marusik, 2016

Zaitunia zagrosica Zamani & Marusik, 2018

Zaitunia zonsteini Fomichev & Marusik, 2013

Family Gnaphosidae Banks, 1892

Berinda hoerwegi Zamani, Chatzaki, Esyunin & Marusik, 2021

Berlandina artaxerxes Zamani, Chatzaki, Esyunin & Marusik, 2021

Berlandina ilika Fomichev & Marusik, 2019

Berlandina koponeni Marusik, Fomichev & Omelko, 2014

Berlandina litvinovi Fomichev & Marusik, 2017

Berlandina mishenini Marusik, Fomichev & Omelko, 2014

Berlandina nakonechnyi Marusik, Fomichev & Omelko, 2014

Berlandina ovtsharenkoi Marusik, Fomichev & Omelko, 2014

Berlandina schenkeli Marusik & Logunov, 1995

Berlandina ubsunurica Marusik & Logunov, 1995

Berlandina yakovlevi Marusik, Fomichev & Omelko, 2014

Callipelis Zamani & Marusik, 2017 [type species: *Callipelis deserticola* Zamani & Marusik, 2017]

Callipelis deserticola Zamani & Marusik, 2017

Cryptodrassus iranicus Zamani, Chatzaki, Esyunin & Marusik, 2021

Drassodes katunensis Marusik, Hippa & Koponen, 1996

Drassodes longispinus Marusik & Logunov, 1995

Drassodes persianus Zamani, Chatzaki, Esyunin & Marusik, 2021

Drassodex tajikistanicus Fomichev & Marusik, 2021

Echemus caspicus Zamani, Chatzaki, Esyunin & Marusik, 2021

Echemus sibiricus Marusik & Logunov, 1995

Fedotovia feti Fomichev & Marusik, 2015

Fedotovia mikhailovi Fomichev & Marusik, 2015

Fedotovia mongolica Marusik, 1993

Gnaphosa banini Marusik & Koponen, 2001

Gnaphosa chola Ovtsharenko & Marusik, 1988

Gnaphosa esyunini Marusik, Fomichev & Omelko, 2014

Gnaphosa khovdensis Marusik, Fomichev & Omelko, 2014

Gnaphosa koponeni Marusik & Omelko, 2014

Gnaphosa qamsarica Zamani, Chatzaki, Esyunin & Marusik, 2021

Gnaphosa rasnitsyni Marusik, 1993

Gnaphosa serzonshteini Fomichev & Marusik, 2017

Gnaphosa tunevae Marusik & Omelko, 2014

Gnaphosa tuvinica Marusik & Logunov, 1992 [replacement name for *Gnaphosa zonsteini*]

Gnaphosa ustyuzhanini Fomichev, Marusik & Omelko, 2013 [synonym of *Gnaphosa rasnitsyni* Marusik, 1993]

Gnaphosa zonsteini Fomichev & Marusik, 2017 [preoccupied, homonym replaced with *Gnaphosa serzonshteini*]

Haplodrassus belgeri Ovtsharenko & Marusik, 1988

Haplodrassus medes Zamani, Chatzaki, Esyunin & Marusik, 2021

Haplodrassus pseudosignifer Marusik, Hippa & Koponen, 1996

Haplodrassus qashqai Zamani, Chatzaki, Esyunin & Marusik, 2021

Iranotricha Zamani & Marusik, 2018 [type species: *Iranotricha lutensis* Zamani & Marusik, 2018]

Iranotricha lutensis Zamani & Marusik, 2018

Marinarozelotes achaemenes Zamani, Chatzaki, Esyunin & Marusik, 2021

Marjanus isfahanicus Zamani, Chatzaki, Esyunin & Marusik, 2021

Micaria atropatene Zamani & Marusik, 2021

Micaria yeniseica Marusik & Koponen, 2002

Nomisia ameretatae Zamani, Chatzaki, Esyunin & Marusik, 2021

Parasyrisca alai Ovtsharenko, Platnick & Marusik, 1995

Parasyrisca alexeevi Ovtsharenko, Platnick & Marusik, 1995

Parasyrisca altaica Ovtsharenko, Platnick & Marusik, 1995

Parasyrisca andarbag Ovtsharenko, Platnick & Marusik, 1995

Parasyrisca andreevae Ovtsharenko, Platnick & Marusik, 1995

Parasyrisca anzobica Ovtsharenko, Platnick & Marusik, 1995

Parasyrisca asiatica Ovtsharenko, Platnick & Marusik, 1995

Parasyrisca balcarica Ovtsharenko, Platnick & Marusik, 1995

Parasyrisca belengish Ovtsharenko, Platnick & Marusik, 1995

Parasyrisca belukha Ovtsharenko, Platnick & Marusik, 1995

Parasyrisca birikchul Ovtsharenko, Platnick & Marusik, 1995

Parasyrisca bucklei Marusik & Fomichev, 2010

Parasyrisca caucasica Ovtsharenko, Platnick & Marusik, 1995

Parasyrisca chikatunovi Ovtsharenko, Platnick & Marusik, 1995

Parasyrisca gissarika Ovtsharenko, Platnick & Marusik, 1995

Parasyrisca golyakovi Marusik & Fomichev, 2016

Parasyrisca guzeripli Ovtsharenko, Platnick & Marusik, 1995

Parasyrisca heimeri Ovtsharenko, Platnick & Marusik, 1995

Parasyrisca hippai Ovtsharenko, Platnick & Marusik, 1995

Parasyrisca holmi Ovtsharenko, Platnick & Marusik, 1995

Parasyrisca iskander Ovtsharenko, Platnick & Marusik, 1995

Parasyrisca khubsugul Ovtsharenko, Platnick & Marusik, 1995

Parasyrisca koksu Ovtsharenko, Platnick & Marusik, 1995

Parasyrisca kosachevi Fomichev, Marusik & Sidorov, 2018

Parasyrisca kurgan Ovtsharenko, Platnick & Marusik, 1995

Parasyrisca kyzylart Ovtsharenko, Platnick & Marusik, 1995

Parasyrisca logunovi Ovtsharenko, Platnick & Marusik, 1995

Parasyrisca mikhailovi Ovtsharenko, Platnick & Marusik, 1995

Parasyrisca narynica Ovtsharenko, Platnick & Marusik, 1995

Parasyrisca otmek Ovtsharenko, Platnick & Marusik, 1995

Parasyrisca paironica Ovtsharenko, Platnick & Marusik, 1995

Parasyrisca pamirica Ovtsharenko, Platnick & Marusik, 1995

Parasyrisca platnicki Marusik, Fomichev & Omelko, 2019

Parasyrisca polchaninovae Marusik, Fomichev & Omelko, 2019

Parasyrisca pshartica Ovtsharenko, Platnick & Marusik, 1995

Parasyrisca schenkeli Ovtsharenko & Marusik, 1988

Parasyrisca shakhristanica Ovtsharenko, Platnick & Marusik, 1995

Parasyrisca songi Marusik & Fritzén, 2009

Parasyrisca sulaki Fomichev, Marusik & Sidorov, 2018

Parasyrisca susamyr Ovtsharenko, Platnick & Marusik, 1995

Parasyrisca szinetari Marusik, Fomichev & Omelko, 2019

Parasyrisca terskei Ovtsharenko, Platnick & Marusik, 1995

Parasyrisca tronovorum Fomichev, Marusik & Sidorov, 2018

Parasyrisca turkenica Ovtsharenko, Platnick & Marusik, 1995

Parasyrisca tyshchenkoi Ovtsharenko, Platnick & Marusik, 1995

Parasyrisca ulykpani Ovtsharenko, Platnick & Marusik, 1995

Parasyrisca vakhanski Ovtsharenko, Platnick & Marusik, 1995

Parasyrisca vorobica Ovtsharenko, Platnick & Marusik, 1995

Pterotricha arzhantsevi Fomichev, Marusik & Koponen, 2018

Pterotricha kovblyuki Zamani & Marusik, 2018

Pterotricha montana Zamani & Marusik, 2018

Scotophaeus anahita Zamani, Chatzaki, Esyunin & Marusik, 2021

Scotophaeus elburzensis Zamani, Chatzaki, Esyunin & Marusik, 2021

Shaitan Kovblyuk, Kastrygina & Marusik, 2013 [type species: *Shaitan elchini* Kovblyuk, Kastrygina & Marusik, 2013]

Shaitan angramainyu Zamani & Marusik, 2021

Shaitan elchini Kovblyuk, Kastrygina & Marusik, 2013

Sosticus montanus Zamani, Chatzaki, Esyunin & Marusik, 2021

Synaphosus dubius Marusik & Omelko, 2018

Synaphosus jaegeri Marusik & Omelko, 2018

Synaphosus lehtineni Marusik & Omelko, 2018

Synaphosus martinezi Zamani, Chatzaki, Esyunin & Marusik, 2021

Synaphosus mongolicus Marusik & Fomichev, 2016

Synaphosus ovtsharenkoi Marusik & Fomichev, 2016

Synaphosus saidovi Marusik & Fomichev, 2016

Synaphosus shmakovi Marusik & Fomichev, 2016

Tuvadrassus Marusik & Logunov, 1995 [type species: *Drassodes tegulatus* Schenkel, 1963 = *Haplodrassus tegulatus* (Schenkel, 1963); synonym of *Haplodrassus* Chamberlin, 1922]

Zagrotes Zamani, Chatzaki, Esyunin & Marusik, 2021 [type species: *Zagrotes apophysalis* Zamani, Chatzaki, Esyunin & Marusik, 2021]

Zagrotes apophysalis Zamani, Chatzaki, Esyunin & Marusik, 2021

Zagrotes bifurcatus (Zamani, Chatzaki, Esyunin & Marusik, 2021) [= *Berinda bifurcata* Zamani, Chatzaki, Esyunin & Marusik, 2021]

Zagrotes borna Zamani & Marusik, 2021

Zagrotes parla Zamani & Marusik, 2021

Zelotes anatolyi Fomichev & Marusik, 2021

Zelotes hyrcanus Zamani, Chatzaki, Esyunin & Marusik, 2021

Zelotes khatlonicus Fomichev & Marusik, 2021

Zelotes mikhailovi Marusik, 1995

Family Hahniidae Bertkau, 1878

Hahnia deiocesi Zamani & Marusik, 2021

Hahnia larseni Marusik, 2017

Hahnia sibirica Marusik, Hippa & Koponen, 1996

Pacifantistea Marusik, 2011 [type species:*Pacifantistea ovtchinnikovi* Marusik, 2011]

Pacifantistea ovtchinnikovi Marusik, 2011

Family Hersiliidae Thorell, 1869

Bastanius Mirshamsi, Zamani & Marusik, 2016 [type species: *Bastanius kermanensis* Mirshamsi, Zamani & Marusik, 2016]

Bastanius foordi (Marusik & Fet, 2009) [= *Hersiliola foordi* Marusik & Fet, 2009]

Bastanius kermanensis Mirshamsi, Zamani & Marusik, 2016

Deltshevia Marusik & Fet, 2009 [type species: *Deltshevia danovi* Marusik & Fet, 2009]

Deltshevia danovi Marusik & Fet, 2009

Deltshevia gromovi Marusik & Fet, 2009

Deltshevia taftanensis Zamani & Marusik, 2021

Duninia Marusik & Fet, 2009 [type species: *Duninia baehrae* Marusik & Fet, 2009]

Duninia baehrae Marusik & Fet, 2009

Duninia darvishi Mirshamsi & Marusik, 2013

Duninia grodnitskyi Zamani & Marusik, 2018

Duninia rheimsae Marusik & Fet, 2009

Hersilia talebii Mirshamsi, Zamani & Marusik, 2016

Hersiliola artemisiae Zamani, Mirshamsi & Marusik, 2017

Hersiliola esyunini Marusik & Fet, 2009

Hersiliola lindbergi Marusik & Fet, 2009

Hersiliola sternbergsi Marusik & Fet, 2009

Hersiliola turcica Marusik, Kunt & Yağmur, 2010

Ovtsharenkoia Marusik & Fet, 2009 [type species: Hersiliola pallida Kroneberg, 1875 = *Ovtsharenkoia pallida* (Kroneberg, 1875)]

Family Linyphiidae Blackwall, 1859

Acanoides Sun, Marusik & Tu, 2014 [type species: *Acanoides beijingensis* Sun, Marusik & Tu, 2014]

Acanoides beijingensis Sun, Marusik & Tu, 2014

Acanthoneta Eskov & Marusik, 1992 [type species: *Lepthyphantes aggressus* Chamberlin & Ivie, 1943 = *Acanthoneta aggressa* (Chamberlin & Ivie, 1943)]

Acanthoneta dokutchaevi (Eskov & Marusik, 1994) [= *Poeciloneta dokutchaevi*

Eskov & Marusik, 1994]

Agyneta parasaxatilis Marusik, Hippa & Koponen, 1996 [synonym of *Agyneta pseudosaxatilis Tanasevitch, 1984*]

Agyneta yakutsaxatilis Marusik & Koponen, 2002 [synonym of *Agyneta amersaxatilis* Saaristo & Koponen, 1998]

Agyphantes Saaristo & Marusik, 2004 [type species: *Agyphantes sakhalinensis* Saaristo & Marusik, 2004]

Agyphantes sajanensis (Eskov & Marusik, 1994) [= *Lepthyphantes sajanensis* Eskov & Marusik, 1994]

Agyphantes sakhalinensis Saaristo & Marusik, 2004

Arcterigone Eskov & Marusik, 1994 [type species: *Erigone pilifrons* L. Koch, 1879 = *Arcterigone pilifrons* (L. Koch, 1879)]

Arcuphantes curvomarginatus Ma, Marusik & Tu, 2016

Arcuphantes dentatus Ma, Marusik & Tu, 2016

Bifurcia oligerae Marusik, Omelko & Koponen, 2016

Bifurcia tanasevitchi Marusik, Omelko & Koponen, 2016

Centromerus amurensis Eskov & Marusik, 1992

Centromerus pacificus Eskov & Marusik, 1992

Centromerus ussuricus Eskov & Marusik, 1992

Ceratinella kurenshchikovi Marusik & Gnelitsa, 2009

Concavocephalus eskovi Marusik & Tanasevitch, 2003

Conothorax Eskov & Marusik, 1992 [preoccupied, homonym replaced with *Connithorax,* type species: *Conithorax barbatus* Eskov, 1988 = *Connithorax barbatus* (Eskov, 1988)]

Dicymbium yaginumai Eskov & Marusik, 1994

Epibellowia pacifica (Eskov & Marusik, 1992) [= *Wubanoides pacificus* Eskov & Marusik, 1992]

Erigonoplus sibiricus Eskov & Marusik, 1997

Eskovia Marusik & Saaristo, 1999 [type species: *Minicia exarmata* Eskov, 1989 = *Eskovia exarmata* (Eskov, 1989)]

Eskovia mongolica Marusik & Saaristo, 1999

Flagelliphantes sterneri (Eskov & Marusik, 1994) [= *Lepthyphantes sterneri* Eskov & Marusik, 1994]

Gongylidioides protegulus Tanasevitch & Marusik, 2019

Hilaira banini Marusik & Tanasevitch, 2003

Hilaira ryabukhini Eskov & Marusik, 1991 [synonym of *Hilaira canaliculata* (Emerton, 1915)]

Hybauchenidium holmi Marusik, 1988 [synonym of *Hybauchenidium aquilonare* (L. Koch, 1879)]

Hypselistes basarukini Marusik & Leech, 1993

Hypselistes kolymensis Marusik & Leech, 1993

Lasiargus zhui Eskov & Marusik, 1994

Lidia Saaristo & Marusik, 2004 [type species: *Lidia tarabaevi* Saaristo & Marusik, 2004]

Lidia tarabaevi Saaristo & Marusik, 2004

Masikia bizini Nekhaeva, Marusik & Buckle, 2019

Mughiphantes chuktshorum (Marusik, 1991) [= *Lepthyphantes chuktshorum* Marusik, 1991, synonym of *Mughiphantes sobrius* (Thorell, 1871)]

Neriene subarctica Marusik, 1991

Obscuriphantes pseudoobscurus (Marusik, Hippa & Koponen, 1996) [= *Lepthyphantes pseudoobscurus* Marusik, Hippa & Koponen, 1996]

Oreoneta banffkluane Saaristo & Marusik, 2004

Oreoneta beringiana Saaristo & Marusik, 2004

Oreoneta eskimopoint Saaristo & Marusik, 2004

Oreoneta eskovi Saaristo & Marusik, 2004

Oreoneta fennica Saaristo & Marusik, 2004

Oreoneta fortyukon Saaristo & Marusik, 2004

Oreoneta herschel Saaristo & Marusik, 2004

Oreoneta kurile Saaristo & Marusik, 2004

Oreoneta logunovi Saaristo & Marusik, 2004

Oreoneta magaputo Saaristo & Marusik, 2004

Oreoneta mineevi Saaristo & Marusik, 2004

Oreoneta repeater Saaristo & Marusik, 2004

Oreoneta sepe Saaristo & Marusik, 2004

Oreoneta tienshangensis Saaristo & Marusik, 2004

Oreoneta tuva Saaristo & Marusik, 2004

Oreoneta uralensis Saaristo & Marusik, 2004

Oreoneta vogelae Saaristo & Marusik, 2004

Oreoneta wyomingia Saaristo & Marusik, 2004

Oryphantes tes (Marusik, Hippa & Koponen, 1996) [= *Lepthyphantes tes* Marusik, Hippa & Koponen, 1996, synonym of *Oryphantes geminus* (Tanasevitch, 1982)]

Pacifiphantes Eskov & Marusik, 1994 [type species: *Pacifiphantes zakharovi* Eskov & Marusik, 1994]

Pacifiphantes zakharovi Eskov & Marusik, 1994

Panamomops depilis Eskov & Marusik, 1994

Paratmeticus Marusik & Koponen, 2010 [type species: *Oedothorax bipunctis* Bösenberg & Strand, 1906 = *Paratmeticus bipunctis* (Bösenberg & Strand, 1906)]

Parawubanoides Eskov & Marusik, 1992 [type species: *Linyphia unicornis* O. Pickard-Cambridge, 1873 = *Parawubanoides unicornis* (O. Pickard-Cambridge, 1873)]

Pelecopsis baicalensis Marusik, Koponen & Danilov, 2001 [synonym of *Pelecopsis parallela* (Wider, 1834)]

Pelecopsis palmgreni Marusik & Esyunin, 1998

Perlongipalpus Eskov & Marusik, 1991 [type species: *Perlongipalpus pinipumilis* Eskov & Marusik, 1991]

Perlongipalpus mannilai Eskov & Marusik, 1991

Perlongipalpus mongolicus Marusik & Koponen, 2008

Perlongipalpus pinipumilis Eskov & Marusik, 1991

Perlongipalpus saaristoi Marusik & Koponen, 2008

Perro tshuktshorum (Eskov & Marusik, 1991) [= *Pero tshuktshorum* Eskov &

Marusik, 1991]

Poeciloneta tanasevitchi Marusik, 1991

Poeciloneta yanensis Marusik & Koponen, 2002 [synonym of *Poeciloneta variegata* (Blackwall, 1841)]

Praestigia eskovi Marusik, Gnelitsa & Koponen, 2008

Praestigia makarovae Marusik, Gnelitsa & Koponen, 2008

Praestigia sibirica Marusik, Gnelitsa & Koponen, 2008

Praestigia uralensis Marusik, Gnelitsa & Koponen, 2008

Procerocymbium buryaticum Marusik & Koponen, 2001

Procerocymbium dondalei Marusik & Koponen, 2001

Procerocymbium jeniseicum Marusik & Koponen, 2001

Pseudowubana Eskov & Marusik, 1992 [type species: *Erigone wagae* O. Pickard-Cambridge, 1873 = *Pseudowubana wagae* (O. Pickard-Cambridge, 1873)]

Saloca ryvkini Eskov & Marusik, 1994

Savignia eskovi Marusik, Koponen & Danilov, 2001

Savignia nenilini Marusik, 1988 [synonym of *Savignia birostra* (Chamberlin & Ivie, 1947)]

Scotinotylus altaicus Marusik, Hippa & Koponen, 1996

Scotinotylus amurensis Eskov & Marusik, 1994

Scotinotylus kimjoopili Eskov & Marusik, 1994

Scotinotylus kolymensis Eskov & Marusik, 1994

Scotinotylus levii Marusik, 1988

Scutpelecopsis Marusik & Gnelitsa, 2009 [type species: *Scutpelecopsis wunderlichi* Marusik & Gnelitsa, 2009]

Scutpelecopsis wunderlichi Marusik & Gnelitsa, 2009

Sibirocyba Eskov & Marusik, 1994 [type species: *Tapinocyba incerta* Kulczyński, 1916 = *Sibirocyba incerta* (Kulczyński, 1916)]

Silometopoides koponeni (Eskov & Marusik, 1994) [= *Silometopus koponeni* Eskov & Marusik, 1994]

Silometopoides mongolensis Eskov & Marusik, 1992

Silometopoides sphagnicola Eskov & Marusik, 1992

Silometopus sachalinensis (Eskov & Marusik, 1994) [= *Silometopoides sachalinensis* Eskov & Marusik, 1994]

Stemonyphantes mikhailovi Omelko & Marusik, 2021

Stemonyphantes verkana Zamani & Marusik, 2021

Styloctetor lehtineni Marusik & Tanasevitch, 1998

Styloctetor logunovi (Eskov & Marusik, 1994) [= *Ceratinopsis logunovi* Eskov & Marusik, 1994]

Styloctetor tuvinensis Marusik & Tanasevitch, 1998

Tanasevitchia Marusik & Saaristo, 1999 [type species: *Minicia uralensis* Tanasevitch, 1983 = *Tanasevitchia uralensis* (Tanasevitch, 1983)]

Thaleria alnetorum Eskov & Marusik, 1992

Thaleria evenkiensis Eskov & Marusik, 1992

Thaleria leechi Eskov & Marusik, 1992

Thaleria sajanensis Eskov & Marusik, 1992

Thaleria sukatchevae Eskov & Marusik, 1992

Theoneta Eskov & Marusik, 1991 [type species: *Microneta saaristoi* Eskov & Marusik, 1991 = *Theoneta saaristoi* (Eskov & Marusik, 1991)]

Theoneta aterrima (Eskov & Marusik, 1991) [= *Microneta aterrima* Eskov & Marusik, 1991]

Theoneta saaristoi (Eskov & Marusik, 1991) [= *Microneta saaristoi* Eskov & Marusik, 1991]

Tibioploides Eskov & Marusik, 1991 [type species: *Tibioploides pacificus* Eskov & Marusik, 1991]

Tibioploides kurenstchikovi Eskov & Marusik, 1991

Tibioploides pacificus Eskov & Marusik, 1991

Uusitaloia Marusik, Koponen & Danilov, 2001 [type species: *Uusitaloia transbaicalica* Marusik, Koponen & Danilov, 2001]

Uusitaloia transbaicalica Marusik, Koponen & Danilov, 2001

Uusitaloia wrangeliana Marusik & Koponen, 2009

Walckenaeria basarukini Eskov & Marusik, 1994

Walckenaeria golovatchi Eskov & Marusik, 1994

Walckenaeria katanda Marusik, Hippa & Koponen, 1996

Walckenaeria palmgreni Eskov & Marusik, 1994

Walckenaeria tystchenkoi Eskov & Marusik, 1994

Zerogone Eskov & Marusik, 1994[type species: *Oedothorax submissellus* Strand, 1907 = *Zerogone submissella* (Strand, 1907)]

Zornella orientalis Marusik, Buckle & Koponen, 2007 [synonym of *Zornella cultrigera* (L. Koch, 1879)]

Family Liocranidae Simon, 1897

Agroeca angirasu Zamani & Marusik, 2021

Mesiotelus caucasicus Zamani & Marusik, 2021

Mesiotelus patricki Zamani & Marusik, 2021

Paratinae Marusik, Zheng & Li, 2009 [subfamily, type genus: *Paratus* Simon, 1898]

Paratus indicus Marusik, Zheng & Li, 2008

Paratus sinensis Marusik, Zheng & Li, 2008

Platnick Marusik & Fomichev, 2020 [type species: *Platnick shablyai* Marusik & Fomichev, 2020]

Platnick astana Marusik & Fomichev, 2020

Platnick sanglok Marusik & Fomichev, 2020

Platnick shablyai Marusik & Fomichev, 2020

Sestakovaia Zamani & Marusik, 2021 [type species: *Sestakovaia hyrcania* Zamani & Marusik, 2021]

Sestakovaia hyrcania Zamani & Marusik, 2021

Family Lycosidae Sundevall, 1833

Acantholycosa aborigenica Zyuzin & Marusik, 1988

Acantholycosa altaiensis Marusik, Azarkina & Koponen, 2004 |

Acantholycosa azarkinae Marusik & Omelko, 2011

Acantholycosa azyuzini Marusik, Hippa & Koponen, 1996

Acantholycosa dudkoromani Marusik, Azarkina & Koponen, 2004

Acantholycosa dudkorum Marusik, Azarkina & Koponen, 2004

Acantholycosa katunensis Marusik, Azarkina & Koponen, 2004

Acantholycosa khakassica Marusik, Azarkina & Koponen, 2004

Acantholycosa kronestedti Fomichev & Marusik, 2018

Acantholycosa kurchumensis Marusik, Azarkina & Koponen, 2004 [synonym of *Acantholycosa katunensis* Marusik, Azarkina & Koponen, 2004]

Acantholycosa levinae Marusik, Azarkina & Koponen, 2004

Acantholycosa logunovi Marusik, Azarkina & Koponen, 2004

Acantholycosa mordkovitchi Marusik, Azarkina & Koponen, 2004

Acantholycosa oligerae Marusik, Azarkina & Koponen, 2004

Acantholycosa paraplumalis Marusik, Azarkina & Koponen, 2004

Acantholycosa petrophila Marusik, Azarkina & Koponen, 2004

Acantholycosa plumalis Marusik, Azarkina & Koponen, 2004

Acantholycosa sayanensis Marusik, Azarkina & Koponen, 2004

Acantholycosa spinembolus Marusik, Azarkina & Koponen, 2004

Acantholycosa sterneri (Marusik, 1993) [= *Pardosa sterneri* Marusik, 1993]

Acantholycosa sundukovi Marusik, Azarkina & Koponen, 2004

Acantholycosa tarbagataica Marusik & Logunov, 2011

Acantholycosa vahterae Fomichev & Marusik, 2018

Acantholycosa valriae Omelko, Komisarenko & Marusik, 2016

Acantholycosa zinchenkoi Marusik, Azarkina & Koponen, 2004

Acantholycosa zonsteini Marusik & Omelko, 2017

Alopecosa akkolka Marusik, 1995

Alopecosa mikhailovi Omelko, Marusik & Koponen, 2013

Alopecosa osa Marusik, Hippa & Koponen, 1996

Alopecosa saurica Marusik, 1995

Alopecosa sokhondoensis Logunov & Marusik, 1995

Alopecosa tanakai Omelko & Marusik, 2008

Alopecosa zyuzini Logunov & Marusik, 1995

Dzhungarocosa Fomichev & Marusik, 2017 [type species: *Dzhungarocosa omelkoi* Fomichev & Marusik, 2017]

Dzhungarocosa ballarini Fomichev & Marusik, 2017

Dzhungarocosa omelkoi Fomichev & Marusik, 2017

Dzhungarocosa zhishengi Fomichev & Marusik, 2017

Evippa caucasica Marusik, Guseinov & Koponen, 2003

Evippa sibirica Marusik, 1995

Gulocosa Marusik, Omelko & Koponen, 2015 [type species: *Gulocosa eskovi* Marusik, Omelko & Koponen, 2015]

Gulocosa eskovi Marusik, Omelko & Koponen, 2015

Halocosa apsheronica (Marusik, Guseinov & Koponen, 2003) [= *Evippa apsheronica* Marusik, Guseinov & Koponen, 2003, synonym of *Halocosa cereipes* (L. Koch, 1878)]

Melecosa Marusik, Omelko & Koponen, 2015 [type species: *Sibirocosa alpina* Marusik, Azarkina & Koponen, 2004 = *Melecosa alpina* (Marusik, Azarkina & Koponen, 2004)]

Melecosa alpina (Marusik, Azarkina & Koponen, 2004) [= *Sibirocosa alpina* Marusik, Azarkina & Koponen, 2004]

Mongolicosa Marusik, Azarkina & Koponen, 2004 [type species: *Mongolicosa glupovi* Marusik, Azarkina & Koponen, 2004]

Mongolicosa azarkinae Fomichev & Marusik, 2018

Mongolicosa buryatica Marusik, Azarkina & Koponen, 2004

Mongolicosa cherepanovi Fomichev & Marusik, 2018

Mongolicosa glupovi Marusik, Azarkina & Koponen, 2004

Mongolicosa gobiensis Marusik, Azarkina & Koponen, 2004

Mongolicosa mongolensis Marusik, Azarkina & Koponen, 2004

Mongolicosa ozkutuki Fomichev & Marusik, 2018

Mongolicosa przhewalskii Fomichev & Marusik, 2017

Mongolicosa songi Marusik, Azarkina & Koponen, 2004

Mongolicosa uncia Fomichev & Marusik, 2017

Pardosa azerifalcata Marusik, Guseinov & Koponen, 2003

Pardosa baraan Logunov & Marusik, 1995

Pardosa bukukun Logunov & Marusik, 1995 [synonym of *Pardosa hanrasanensis* Jo & Paik, 1984]

Pardosa dzheminey Marusik, 1995

Pardosa eskovi Kronestedt & Marusik, 2011

Pardosa fengi Marusik, Nadolny & Omelko, 2013

Pardosa fomichevi Kronestedt, Marusik & Omelko, 2014

Pardosa fritzeni Ballarin, Marusik, Omelko & Koponen, 2012

Pardosa gromovi Ballarin, Marusik, Omelko & Koponen, 2012

Pardosa gusarensis Marusik, Guseinov & Koponen, 2003

Pardosa jeniseica Eskov & Marusik, 1995

Pardosa koponeni Nadolny, Omelko, Marusik & Blagoev, 2016

Pardosa lii Marusik, Nadolny & Omelko, 2013

Pardosa logunovi Kronestedt & Marusik, 2011

Pardosa mikhailovi Ballarin, Marusik, Omelko & Koponen, 2012

Pardosa mirzakhaniae Shafaie, Mirshamsi, Aliabadian, Moradmand & Marusik, 2018

Pardosa nenilini Marusik, 1995

Pardosa oksalai Marusik, Hippa & Koponen, 1996

Pardosa ovtchinnikovi Ballarin, Marusik, Omelko & Koponen, 2012

Pardosa pantinii Ballarin, Marusik, Omelko & Koponen, 2012

Pardosa persiana Marusik & Nadolny, 2020

Pardosa persica Marusik, Ballarin & Omelko, 2012 [preoccupied, homonym replaced with *Pardosa persiana*]

Pardosa pseudolapponica Marusik, 1995

Pardosa pseudomixta Marusik & Fritzén, 2009

Pardosa svatoni Marusik, Nadolny & Omelko, 2013

Pardosa trottai Ballarin, Marusik, Omelko & Koponen, 2012

Pardosa tyshchenkoi Zyuzin & Marusik, 1989

Pardosa zonsteini Ballarin, Marusik, Omelko & Koponen, 2012

Pardosa zyuzini Kronestedt & Marusik, 2011

Passiena bayi Omelko & Marusik, 2020

Piratula logunovi Omelko, Marusik & Koponen, 2011

Piratula raika Zamani & Marusik, 2021

Pyrenecosa Marusik, Azarkina & Koponen, 2004 [type species: *Lycosa rupicola* Dufour, 1821 = *Pyrenecosa rupicola* (Dufour, 1821)]

Sibirocosa Marusik, Azarkina & Koponen, 2004 [type species: *Sibirocosa kolymensis* Marusik, Azarkina & Koponen, 2004]

Sibirocosa kolymensis Marusik, Azarkina & Koponen, 2004

Sibirocosa koponeni Omelko & Marusik, 2013

Sibirocosa manchurica Marusik, Azarkina & Koponen, 2004

Sibirocosa nadolnyi Omelko & Marusik, 2013

Sibirocosa trilikauskasi Omelko & Marusik, 2013

Family Miturgidae Simon, 1886

Zora huseynovi Zamani & Marusik, 2017

Family Nesticidae Simon, 1894

Aituaria iranica Zamani & Marusik, 2021

Carpathonesticus eriashvilii Marusik, 1987

Carpathonesticus mamajevae Marusik, 1987

Eopopino budrysi Eskov & Marusik, 1992 [fossil]

Eopopino palanga Eskov & Marusik, 1992 [fossil]

Nesticella kerzhneri (Marusik, 1987) [= *Howaia kerzhneri* Marusik, 1987]

Family Oecobiidae Blackwall, 1862

Oecobius fahimii Zamani & Marusik, 2018

Oecobius ferdowsii Mirshamsi, Zamani & Marusik, 2017

Oecobius ilamensis Zamani, Mirshamsi & Marusik, 2017

Family Oonopidae Simon, 1890

Ferchestina Saaristo & Marusik, 2004 [type species: *Ferchestina storozhenkoi* Saaristo & Marusik, 2004 = *Orchestina storozhenkoi* (Saaristo & Marusik, 2004); synonym of *Orchestina* Simon, 1882]

Nale Saaristo & Marusik, 2008 [type species: *Opopaea lena* Suman, 1965 = *Opopaea apicalis* (Simon, 1893); synonym of *Opopaea* Simon, 1892]

Opopaea alje Saaristo & Marusik, 2008

Opopaea botswana Saaristo & Marusik, 2008

Opopaea gabon Saaristo & Marusik, 2008

Opopaea gaborone Saaristo & Marusik, 2008

Opopaea sudan Saaristo & Marusik, 2008

Orchestina storozhenkoi (Saaristo & Marusik, 2004) [= *Ferchestina storozhenkoi* Saaristo & Marusik, 2004]

Spinestis Saaristo & Marusik, 2009 [type species: *Spinestis nikita* Saaristo & Marusik, 2009]

Spinestis nikita Saaristo & Marusik, 2009

Family Palpimanidae Thorell, 1870

Boagrius simoni Zonstein & Marusik, 2020

Chedimanops Zonstein & Marusik, 2017 [type species: *Chedimanops eskovi* Zonstein & Marusik, 2017]

Chedimanops eskovi Zonstein & Marusik, 2017

Chedimanops rwenzorensis Zonstein & Marusik, 2017

Diaphorocellus isalo Zonstein & Marusik, 2020

Diaphorocellus jocquei Zonstein & Marusik, 2020

Hybosidella Zonstein & Marusik, 2017 [type species: *Hybosidella etinde* Zonstein & Marusik, 2017]

Hybosidella etinde Zonstein & Marusik, 2017

Levymanus Zonstein & Marusik, 2013 [type species: *Levymanus gershomi* Zonstein &

Marusik, 2013]

Levymanus dezfulensis Zamani & Marusik, 2020

Levymanus gershomi Zonstein & Marusik, 2013

Levymanus ras Zonstein, Marusik & Kovblyuk, 2017

Palpimanus carmania Zamani & Marusik, 2021

Palpimanus persicus Zamani & Marusik, 2021

Family Philodromidae Thorell, 1870

Apollophanes lenensis Marusik, 1991 [synonym of *Apollophanes macropalpus* (Paik, 1979)]

Philodromus aryy Marusik, 1991

Philodromus utotchkini Marusik, 1991

Philodromus vinokurovi Marusik, 1991

Rhysodromus genoensis Zamani & Marusik, 2021

Rhysodromus medes Zamani & Marusik, 2021

Thanatus kolymensis Marusik, 1991 [synonym of *Thanatus arcticus* Thorell, 1872]

Family Phrurolithidae Banks, 1892

Bosselaerius Zamani & Marusik, 2020 [type species: *Bosselaerius hyrcanicus* Zamani & Marusik, 2020]

Bosselaerius hyrcanicus Zamani & Marusik, 2020

Bosselaerius tajikistanicus Zamani & Marusik, 2020

Labialithus lindemanni (Marusik, Omelko & Koponen, 2020) [= *Phrurolithus lindemanni* Marusik, Omelko & Koponen, 2020]

Phrurolithus azarkinae Zamani & Marusik, 2020

Family Pisauridae Simon, 1890

Dolomedes bukhkaloi Marusik, 1988

Euprosthenops insperatus Zonstein & Marusik, 2021

Family Prodidomidae Simon, 1884

Prodidomus inexpectatus Zamani, Chatzaki, Esyunin & Marusik, 2021

Family Salticidae Blackwall, 1841

Aelurillus laniger Logunov & Marusik, 2000

Asianellus kuraicus Logunov & Marusik, 2000

Chalcovietnamicus Marusik, 1991 [type species: *Chalcoscirtus vietnamensis* Żabka, 1985]

Chalcoscirtus bortolgois Logunov & Marusik, 1999

Chalcoscirtus charynensis Logunov & Marusik, 1999

Chalcoscirtus glacialis sibiricus Marusik, 1991

Chalcoscirtus grishkanae Marusik, 1988

Chalcoscirtus hosseinieorum Logunov, Marusik & Mozaffarian, 2002

Chalcoscirtus hyperboreus Marusik, 1991

Chalcoscirtus iranicus Logunov & Marusik, 1999

Chalcoscirtus kamchik Marusik, 1991

Chalcoscirtus karakurt Marusik, 1991

Chalcoscirtus kirghisicus Marusik, 1991

Chalcoscirtus koponeni Logunov & Marusik, 1999

Chalcoscirtus michailovi Logunov & Marusik, 1999

Chalcoscirtus minutus Marusik, 1990

Chalcoscirtus molo Marusik, 1991

Chalcoscirtus nenilini Marusik, 1990

Chalcoscirtus paraansobicus Marusik, 1990

Chalcoscirtus parvulus Marusik, 1991

Chalcoscirtus pavuk Marusik, 1991 [synonym of *Chalcoscirtus parvulus* Marusik, 1991]

Chalcoscirtus platnicki Marusik, 1995

Chalcoscirtus sinevi Marusik, Fomichev & Vahtera, 2018

Chalcoscirtus talturaensis Logunov & Marusik, 2000

Chalcoscirtus tanasevichi Marusik, 1991

Chalcoscirtus tanyae Logunov & Marusik, 1999

Chalcoscirtus zyuzini Marusik, 1991

Dendryphantes ovchinnikovi Logunov & Marusik, 1994

Euophrys friedmani Marusik, 2019

Euophrys proszynskii Logunov, Cutler & Marusik, 1993

Euophrys uralensis Logunov, Cutler & Marusik, 1993

Eupoa daklak Logunov & Marusik, 2014

Eupoa lehtineni Logunov & Marusik, 2014

Eupoa lobli Logunov & Marusik, 2014

Eupoa pappi Logunov & Marusik, 2014

Eupoa pulchella Logunov & Marusik, 2014

Eupoa schwendingeri Logunov & Marusik, 2014

Eupoa thailandica Logunov & Marusik, 2014

Evarcha proszynskii Marusik & Logunov, 1998

Helicius chikunii (Logunov & Marusik, 1999) [= *Pseudicius chikunii* Logunov & Marusik, 1999]

Lyssomanes ecuadoricus Logunov & Marusik, 2003

Lyssomanes janauari Logunov & Marusik, 2003

Lyssomanes onkonensis Logunov & Marusik, 2003

Lyssomanes trinidadus Logunov & Marusik, 2003

Lyssomanes waorani Logunov & Marusik, 2003

Marusyllus aralicus (Logunov & Marusik, 2003) [= *Yllenus aralicus* Logunov & Marusik, 2003]

Marusyllus kalkamanicus (Logunov & Marusik, 2000) [= *Yllenus kalkamanicus* Logunov & Marusik, 2000]

Marusyllus karnai (Logunov & Marusik, 2003) [= *Yllenus karnai* Logunov & Marusik, 2003]

Marusyllus kotchevnik (Logunov & Marusik, 2003) [= *Yllenus kotchevnik* Logunov & Marusik, 2003]

Marusyllus murgabicus (Logunov & Marusik, 2003) [= *Yllenus murgabicus* Logunov & Marusik, 2003]

Marusyllus pamiricus (Logunov & Marusik, 2003) [= *Yllenus pamiricus* Logunov & Marusik, 2003]

Marusyllus pseudobajan (Logunov & Marusik, 2003) [= *Yllenus pseudobajan* Logunov & Marusik, 2003]

Marusyllus tuvinicus (Logunov & Marusik, 2000) [= *Yllenus tuvinicus* Logunov & Marusik, 2000]

Marusyllus uzbekistanicus (Logunov & Marusik, 2003) [= *Yllenus uzbekistanicus* Logunov & Marusik, 2003]

Pelpaucus Logunov, Marusik, Rakov, 1999 [subgenus of *Pellenes*, type species *P. limbatus* Kulczyński, 1895]

Pelmultus Logunov, Marusik, Rakov, 1999 [subgenus of *Pellenes*, type species *Attus geniculatus* Simon, 1868 = *Pellenes geniculatus* (Simon, 1868)]

Pelmirus Logunov, Marusik, Rakov, 1999 [subgenus of *Pellenes*, type species *P. dilutus* Logunov, 1995]

Pellenes amazonka Logunov, Marusik & Rakov, 1999

Pellenes badkhyzicus Logunov, Marusik & Rakov, 1999

Pellenes bonus Logunov, Marusik & Rakov, 1999

Pellenes borisi Logunov, Marusik & Rakov, 1999

Pellenes karakumensis Logunov, Marusik & Rakov, 1999

Pellenes logunovi Marusik, Hippa & Koponen, 1996

Pellenes pamiricus Logunov, Marusik & Rakov, 1999

Pellenes pseudobrevis Logunov, Marusik & Rakov, 1999

Pellenes sibiricus Logunov & Marusik, 1994

Pellenes sytchevskayae Logunov, Marusik & Rakov, 1999

Pellenes turkmenicus Logunov, Marusik & Rakov, 1999

Pseudomogrus algarvensis (Logunov & Marusik, 2003) [= *Yllenus algarvensis* Logunov & Marusik, 2003]

Pseudomogrus bakanas (Logunov & Marusik, 2003) [= *Yllenus bakanas* Logunov &

Marusik, 2003]

Pseudomogrus bucharaensis (Logunov & Marusik, 2003) [= *Yllenus bucharaensis* Logunov & Marusik, 2003]

Pseudomogrus dalaensis (Logunov & Marusik, 2003) [= *Yllenus dalaensis* Logunov & Marusik, 2003]

Pseudomogrus gavdos (Logunov & Marusik, 2003) [= *Yllenus gavdos* Logunov & Marusik, 2003]

Pseudomogrus guseinovi (Logunov & Marusik, 2003) [= *Yllenus guseinovi* Logunov & Marusik, 2003]

Pseudomogrus halugim (Logunov & Marusik, 2003) [= *Yllenus halugim* Logunov & Marusik, 2003]

Pseudomogrus mirabilis (Logunov & Marusik, 2003) [= *Yllenus mirabilis* Logunov & Marusik, 2003]

Pseudomogrus nigritarsis (Logunov & Marusik, 2003) [= *Yllenus nigritarsis* Logunov & Marusik, 2003]

Pseudomogrus nurataus (Logunov & Marusik, 2003) [= *Yllenus nurataus* Logunov & Marusik, 2003]

Pseudomogrus pavlenkoae (Logunov & Marusik, 2003) [= *Yllenus pavlenkoae* Logunov & Marusik, 2003]

Pseudomogrus pseudovalidus (Logunov & Marusik, 2003) [= *Yllenus pseudovalidus* Logunov & Marusik, 2003]

Pseudomogrus shakhsenem (Logunov & Marusik, 2003) [= *Yllenus shakhsenem* Logunov & Marusik, 2003]

Pseudomogrus tamdybulak (Logunov & Marusik, 2003) [= *Yllenus tamdybulak* Logunov & Marusik, 2003]

Pseudomogrus zhilgaensis (Logunov & Marusik, 2003) [= *Yllenus zhilgaensis* Logunov & Marusik, 2003]

Synageles morsei Logunov & Marusik, 1999

Yllenus charynensis Logunov & Marusik, 2003

Yllenus dunini Logunov & Marusik, 2003

Yllenus erzinensis Logunov & Marusik, 2003

Yllenus gajdosi Logunov & Marusik, 2000

Yllenus karakumensis Logunov & Marusik, 2003

Yllenus kononenkoi Logunov & Marusik, 2003

Yllenus lyachovi Logunov & Marusik, 2000

Yllenus rotundiorificus Logunov & Marusik, 2000

Yllenus turkestanicus Logunov & Marusik, 2003

Yllenus uiguricus Logunov & Marusik, 2003

Yllenus zyuzini Logunov & Marusik, 2003

Family Scytodidae Blackwall, 1864

Scytodes kumonga Zamani & Marusik, 2020

Family Segestriidae Simon, 1893

Segestria fengi Fomichev & Marusik, 2020

Segestria mirshamsii Marusik & Omelko, 2014

Segestria nekhaevae Fomichev & Marusik, 2020

Segestria shtoppelae Fomichev & Marusik, 2020

Family Sicariidae Keyserling, 1880

Loxosceles coheni Zamani, Mirshamsi & Marusik, 2021

Loxosceles turanensis Zamani, Mirshamsi & Marusik, 2021

Family Stenochilidae Thorell, 1873

Colopea lehtineni Zheng, Marusik & Li, 2009

Family Synaphridae Wunderlich, 1986

Synaphris lehtineni Marusik, Gnelitsa & Kovblyuk, 2005

Synaphris orientalis Marusik & Lehtinen, 2003

Synaphris wunderlichi Marusik & Zonstein, 2011

Family Tetragnathidae Menge, 1866

Diphya foordi Omelko, Marusik & Lyle, 2020

Diphya leroyorum Omelko, Marusik & Lyle, 2020

Diphya tanikawai Marusik, 2017 [synonym of *Diphya simoni* Kauri, 1950]

Diphya vanderwaltae Omelko, Marusik & Lyle, 2020

Diphya wesolowskae Omelko, Marusik & Lyle, 2020

Meta americana Marusik & Koponen, 1992 [synonym of *Meta ovalis* (Gertsch, 1933)]

Meta manchurica Marusik & Koponen, 1992

Metellina haddadi Marusik & Larsen, 2018

Nanometa hippai (Marusik & Omelko, 2017) [= *Nediphya hippai* Marusik & Omelko, 2017]

Nanometa lehtineni (Marusik & Omelko, 2017) [= *Nediphya lehtineni* Marusik & Omelko, 2017]

Nanometa lyleae (Marusik & Omelko, 2017) [= *Nediphya lyleae* Marusik & Omelko, 2017]

Nanometa padillai (Marusik & Omelko, 2017) [= *Nediphya padillai* Marusik & Omelko, 2017]

Nediphya Marusik & Omelko, 2017 [type species: *Nediphya lehtineni* Marusik & Omelko, 2017 = *Nanometa lehtineni* (Marusik & Omelko, 2017); synonym of *Nanometa* Simon, 1908]

Tetragnatha kovblyuki Marusik, 2010 [synonym of *Tetragnatha shoshone* Levi, 1981]

Family Theridiidae Sundevall, 1833

Carniella nepalensis Tanasevitch & Marusik, 2020

Craspedisia yapchoontecki Penney & Marusik, 2012 [fossil]

Enoplognatha monstrabilis Marusik & Logunov, 2002

Episinus balticus Marusik & Penney, 2004 [fossil]

Episinus eskovi Marusik & Penney, 2004 [fossil]

Episinus mikhailovi Zamani & Marusik, 2021

Euryopis schwendingeri Zamani & Marusik, 2021

Glebych Eskov & Marusik, 2021[type species: *Glebych minutissimus* Eskov & Marusik, 2021]

Glebych minutissimus Eskov & Marusik, 2021

Lasaeola baltica (Marusik & Penney, 2004) [= *Euryopis baltica* Marusik & Penney, 2004, fossil]

Lasaeola dbari Kovblyuk, Marusik & Omelko, 2012

Latrodectus tadzhicus Marusik & Tarabaev, 1995 [synonym of *Latrodectus dahli* Levi, 1959]

Nanomysmena palanga Marusik & Penney, 2004 [fossil]

Nanomysmena petrunkevitchi Marusik & Penney, 2004 [fossil]

Nanomysmena pseudogracilis Marusik & Penney, 2004 [fossil]

Ohlertidion thaleri (Marusik, 1988) [= *Theridion thaleri* Marusik, 1988]

Phoroncidia borea Logunov & Marusik, 1992 [synonym of *Phoroncidia pilula* (Karsch, 1879)]

Rhomphaea hyrcana (Logunov & Marusik, 1990) [= *Argyrodes hyrcana* Logunov & Marusik, 1990]

Tekellina yoshidai Marusik & Omelko, 2017

Theridion arsia Zamani & Marusik, 2021

Theridion berendti Marusik & Penney, 2004 [fossil]

Theridion sibiricum Marusik, 1988

Theridion sulawesiense Marusik & Penney, 2004

Yunohamella palmgreni (Marusik & Tsellarius, 1986) [= *Theridion palmgreni* Marusik & Tsellarius, 1986]

Family Thomisidae Sundevall, 1833

Bassaniodes ovadan (Marusik & Logunov, 1995) [= *Xysticus ovadan* Marusik & Logunov, 1995]

Bassaniodes ovcharenkoi (Marusik & Logunov, 1990) [= *Xysticus ovcharenkoi*

Marusik & Logunov, 1990]

Bassaniodes turlan (Marusik & Logunov, 1990) [= *Xysticus turlan* Marusik & Logunov, 1990]

Bassaniodes ulkan (Marusik & Logunov, 1990) [= *Xysticus ulkan* Marusik & Logunov, 1990]

Cozyptila Lehtinen & Marusik, 2005 [type species: *Ozyptila blackwalli* Simon, 1875 = *Cozyptila blackwalli* (Simon, 1875)]

Cozyptila guseinovorum Marusik & Kovblyuk, 2005

Cozyptila thaleri Marusik & Kovblyuk, 2005 [synonym of *Cozyptila nigristernum* (Dalmas, 1922)]

Diaea osmanii Zamani & Marusik, 2017

Ozyptila kaszabi Marusik & Logunov, 2002

Ozyptila sakhalinensis Ono, Marusik & Logunov, 1990

Ozyptila utotchkini Marusik, 1990

Psammitis abramovi (Marusik & Logunov, 1995) [= *Xysticus abramovi* Marusik & Logunov, 1995]

Psammitis courti (Marusik & Omelko, 2014) [= *Xysticus courti* Marusik & Omelko, 2014]

Psammitis gobiensis (Marusik & Logunov, 2002) [= *Xysticus gobiensis* Marusik & Logunov, 2002]

Psammitis nenilini (Marusik, 1989) [= *Xysticus nenilini* Marusik, 1989]

Psammitis seserlig (Logunov & Marusik, 1994) [= *Xysticus seserlig* Logunov & Marusik, 1994]

Psammitis tyshchenkoi (Marusik & Logunov, 1995) [= *Xysticus tyshchenkoi* Marusik & Logunov, 1995]

Psammitis zonshteini (Marusik, 1989) [= *Xysticus zonshteini* Marusik, 1989]

Runcinia tarabayevi Marusik & Logunov, 1990

Spiracme dondalei (Marusik, 1988) [= *Xysticus dondalei* Marusik, 1988, synonym of *Spiracme baltistana* (Caporiacco, 1935)]

Spiracme lehtineni (Fomichev, Marusik & Koponen, 2014) [= *Xysticus lehtineni*

Fomichev, Marusik & Koponen, 2014]

Synema utotchkini Marusik & Logunov, 1995

Thomisus zyuzini Marusik & Logunov, 1990

Tmarus gajdosi Marusik & Logunov, 2002

Xysticus austrosibiricus Logunov & Marusik, 1998

Xysticus bakanas Marusik & Logunov, 1990

Xysticus bermani Marusik, 1994

Xysticus kuzgi Marusik & Logunov, 1990

Xysticus mugur Marusik, 1990

Xysticus palpimirabilis Marusik & Chevrizov, 1990

Xysticus pseudoluctuosus Marusik & Logunov, 1995

Xysticus schenkeli Marusik, 1989 [synonym of *Xysticus pseudobliteus* (Simon, 1880)]

Xysticus sharlaa Marusik & Logunov, 2002

Xysticus taukumkurt Marusik & Logunov, 1990

Xysticus turkmenicus Marusik & Logunov, 1995

Xysticus urgumchak Marusik & Logunov, 1990

Xysticus wunderlichi Logunov, Marusik & Trilikauskas, 2001

Family Titanoecidae Lehtinen, 1967

Titanoeca eca Marusik, 1995

Titanoeca minuta Marusik, 1995

Titanoeca zyuzini Marusik, 1995 [synonym of *Titanoeca liaoningensis* Zhu, Gao & Guan, 1993]

Family Trachelidae Simon, 1897

Orthobula mikhailovi Marusik, 2021

Trachelas crewsae Marusik & Fomichev, 2020

Trachelas tanasevitchi Marusik & Kovblyuk, 2010

Family Zodariidae Thorell, 1881

Acanthinozodium armita Zamani & Marusik, 2021

Acanthinozodium atrisa Zamani & Marusik, 2021

Acanthinozodium diara Zamani & Marusik, 2021

Acanthinozodium dorsa Zamani & Marusik, 2021

Acanthinozodium elburzicum Zamani & Marusik, 2021

Acanthinozodium kiana Zamani & Marusik, 2021

Acanthinozodium masa Zamani & Marusik, 2021

Acanthinozodium niusha Zamani & Marusik, 2021

Acanthinozodium ovtchinnikovi Zamani & Marusik, 2021

Acanthinozodium parmida Zamani & Marusik, 2021

Acanthinozodium parysatis Zamani & Marusik, 2021

Acanthinozodium sorani Zamani & Marusik, 2021

Lachesana dyachkovi Fomichev & Marusik, 2019

Lachesana kavirensis Zamani & Marusik, 2021

Lachesana perseus Zamani & Marusik, 2021

Pax ellipita Zamani & Marusik, 2021

Pax leila Zamani & Marusik, 2021

Trygetus gromovi Marusik, 2011

Trygetus jacksoni Marusik & Guseinov, 2003

Trygetus susianus Zamani & Marusik, 2021

Zodariellum mongolicum Marusik & Koponen, 2001

Zodariellum schmidti Marusik & Koponen, 2001

INFRAORDER MYGALOMORPHAE POCOCK, 1892

Family Bemmeridae Simon, 1903

Atmetochilus koponeni Zonstein & Marusik, 2016

Atmetochilus lehtineni Zonstein & Marusik, 2016

Atmetochilus sumatranus Zonstein & Marusik, 2016

Family Cyrtaucheniidae Simon, 1889

Anemesia koponeni Marusik, Zamani & Mirshamsi, 2014

Family Nemesiidae Simon, 1889

Raveniola ambardzumyani Marusik & Zonstein, 2021

Raveniola mazandaranica Marusik, Zamani & Mirshamsi, 2014

Raveniola montana Zonstein & Marusik, 2012

Raveniola shangrila Zonstein & Marusik, 2012

Raveniola songi Zonstein & Marusik, 2012

Raveniola yunnanensis Zonstein & Marusik, 2012

List 3 List of taxa named after Yuri M. Marusik

CLASS ARACHNIDA LAMARCK, 1801

ORDER ARANEAE CLERCK, 1757

Family Clubionidae Wagner, 1887

Clubiona marusiki Mikhailov, 1990 [Locality: Russia (Far East)]

Clubiona yurii Mikhailov, 2011 [Locality: Mongolia]

Family Gnaphosidae Banks, 1892

Drassodes marusiki Esyunin & Zamani, 2019 [Locality: Iran]

Micaria marusiki Zhang, Song & Zhu, 2001 [Locality: China]

Parasyrisca marusiki Kovblyuk, 2003 [Locality: Ukraine]

Family Linyphiidae Blackwall, 1859

Diplocephalus marusiki Eskov, 1988 [Locality: Russia (north-eastern Siberia)]

Hilaira marusiki Eskov, 1987 [Locality: Russia (north-eastern Siberia), Mongolia]

Mughiphantes marusiki (Tanasevitch, 1988) [= *Lepthyphantes marusiki* Tanasevitch, 1988, Locality: Russia (north-eastern Siberia)]

Family Lycosidae Sundevall, 1833

Acantholycosa marusiki Fomichev & Omelko, 2020 [Locality: Russia (Far East)]

Family Nemesiidae Simon, 1889

Raveniola marusiki Zonstein, Kunt & Yağmur, 2018 [Locality: Iran]

Family Philodromidae Thorell, 1870

Philodromus marusiki (Logunov, 1997) [= *Artanes marusiki* Logunov, 1997, Locality: Russia (West and South Siberia), Mongolia]

Family Pholcidae C. L. Koch, 1850

Belisana marusiki Huber, 2005 [Locality: India]

Family Salticidae Blackwall, 1841

Aelurillus marusiki Azarkina, 2002 [Locality: Iran]

Stenaelurillus marusiki Logunov, 2001 [Locality: Iran]

Marusyllus Prószyński, 2016 [type species: *Marusyllus hamifer* (Simon, 1895) = *Yllenus hamifer* Simon, 1876, Locality: Central Asia, Asiatic Russia: Tuva, Korea]

Yllenus marusiki Logunov, 1993 [Locality: Mongolia]

Family Thomisidae Sundevall, 1833

Xysticus marusiki Ono & Martens, 2005 [Locality: Iran]

ORDER SCORPIONES C. L. KOCH, 1837

Family Buthidae C.L. Koch, 1837

Mesobuthus marusiki Kovarik, Fet, Gantenbein, Graham, Yagmur, Stahlavsky, Poverenni & Nouvruzov, 2022 [Locality: Uzbekistan]

CLASS INSECTA LINNAEUS, 1758

ORDER COLEOPTERA LINNAEUS, 1758

Family Carabidae Latreille, 1802

Carabus (Morphocarabus) chaudoiri marusiki Obydov, 1997 [Locality: Russia]

Family Staphylinidae Lameere, 1900

Syntomium marusiki Ryabukhin, 1992 [Locality: Russia]

ORDER DIPTERA LINNAEUS, 1758

Family Syrphidae Latreille, 1802

Paragus marusiki Sorokina, 2002 [Locality: Russia]

ORDER HEMIPTERA LINNAEUS, 1758

Family Delphacidae Leach, 1815

Pinodoxa pinanorum marusiki Anufriev, 1991 [Locality: Russia]

Family Kinnaridae Muir, 1925

Adolenda marusiki Emeljanov, 2021 [Locality: India]

Family Leptopidae Fieber, 1861

Leptopus marusiki Vinokurov, 2012 [Locality: India]

ORDER LEPIDOPTERA LINNAEUS, 1758

Family Elachistidae Bruand, 1851

Elachista marusiki Kaila, 2018 [Locality: Mongolia]

ORDER PSOCODEA HENNIG, 1966

Family Epipsocidae Pearman, 1936

Bertkauia marusiki Mockford, 2003 [Locality: Russia]
